# Information theory inspired optimization algorithm for efficient service orchestration in distributed systems

**DOI:** 10.1371/journal.pone.0242285

**Published:** 2021-01-04

**Authors:** Matheus Sant’Ana Lima

**Affiliations:** Department of Computer Science, Federal University of São Carlos, São Paulo, Brasil; Torrens University Australia, AUSTRALIA

## Abstract

Distributed Systems architectures are becoming the standard computational model for processing and transportation of information, especially for Cloud Computing environments. The increase in demand for application processing and data management from enterprise and end-user workloads continues to move from a single-node client-server architecture to a distributed multitier design where data processing and transmission are segregated. Software development must considerer the orchestration required to provision its core components in order to deploy the services efficiently in many independent, loosely coupled—physically and virtually interconnected—data centers spread geographically, across the globe. This network routing challenge can be modeled as a variation of the Travelling Salesman Problem (TSP). This paper proposes a new optimization algorithm for optimum route selection using Algorithmic Information Theory. The Kelly criterion for a Shannon-Bernoulli process is used to generate a reliable quantitative algorithm to find a near optimal solution tour. The algorithm is then verified by comparing the results with benchmark heuristic solutions in 3 test cases. A statistical analysis is designed to measure the significance of the results between the algorithms and the entropy function can be derived from the distribution. The tested results shown an improvement in the solution quality by producing routes with smaller length and time requirements. The quality of the results proves the flexibility of the proposed algorithm for problems with different complexities without relying in nature-inspired models such as Genetic Algorithms, Ant Colony, Cross Entropy, Neural Networks, 2opt and Simulated Annealing. The proposed algorithm can be used by applications to deploy services across large cluster of nodes by making better decision in the route design. The findings in this paper unifies critical areas in Computer Science, Mathematics and Statistics that many researchers have not explored and provided a new interpretation that advances the understanding of the role of entropy in decision problems encoded in Turing Machines.

## Introduction

Distributed Information Systems (DS) are growing in popularity across the software industry as it provides more computational and data transmission capacity for applications and become an essential infrastructure that is needed to address the increase in demand for data processing.

DS are used as a cost-efficient way to obtain higher levels of performance by using a cluster of low-capacity machines instead of a unique–single point of failure—large node. A DS is more tolerant to individual machine failures and provides more reliability than a monolithic system.

Parallel computation such as Cloud Computing and High-Performance Computing (HPC) are applications of distributed computing [1].

**Fig 1:**
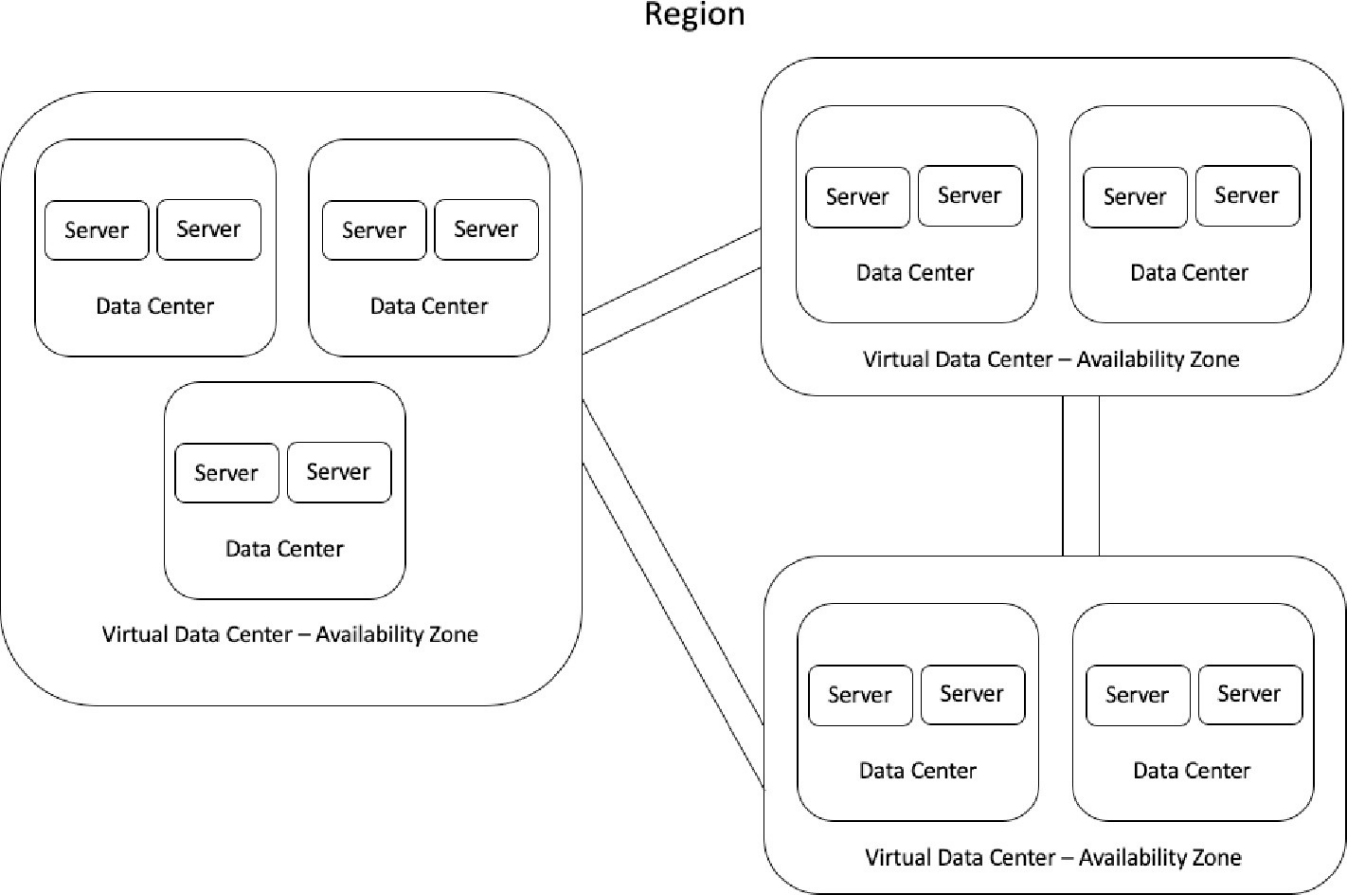
A Virtual Data Center (VDC) has at least 2 physical data centers composed by 2 server nodes. A combination of 3 or more VDC’s is a geographic Region.

The Cloud Computing market is very consolidated as the cost to deploy, expand and operate a global infrastructure and network is very large. As of 2020 there are 3 major companies: Amazon AWS, Microsoft Azure and Google Cloud Platform. Companies can reduce their IT costs by orchestrating efficiently their workloads across different data centers by their respective weight impact, defined as a utility function with the Euclidian distance between nodes and its respective influence on network latency or the financial utilization time-rate cost for a given set of machines. The Fig 1 illustrate the core components of a cloud computing infrastructure service.

The components of a DS are located in many different machines over a network. The communication and orchestration of process are done by sending and receiving messages. The service exposed are defined by the aggregation of components and its interactions provide the software functionality. Systems such as Service-Oriented Architecture (SOA), peer-to-peer(P2P) and Micro-Services are examples of distributed applications.

Deploying and synchronizing components over many distributed cluster of nodes can be very complex due to multiple variables that can affect the quality of the solution such as network latency between data centers at business hours and at on-demand; cost of renting machines from different Computing Providers; shared servers resources utilization (“noisy neighbor effect”- at both virtual machines and bare metal); valorization of the dollar due to macro and micro economics factors; change in processing time due to model of nodes available for a given time period and operating complexity of the technology stack.

There are many algorithms proposed in the literature to solve the routing-scheduling problem such as 2opt, ant colony (AC), greedy algorithm, genetic algorithm (GA), neural networks, Cross Entropy, and simulated annealing (SA) but very limited work is found using Algorithmic Information Theory to find the boundaries of decision problems in Turing Machines. In this paper we propose a variation of the TSP by defining the decision problem for the candidate solution as a Shannon-Bernoulli process that follows a log-normal distribution for the cost distance variable, defined as a dependable utility function for the TSP.

**Fig 2:**
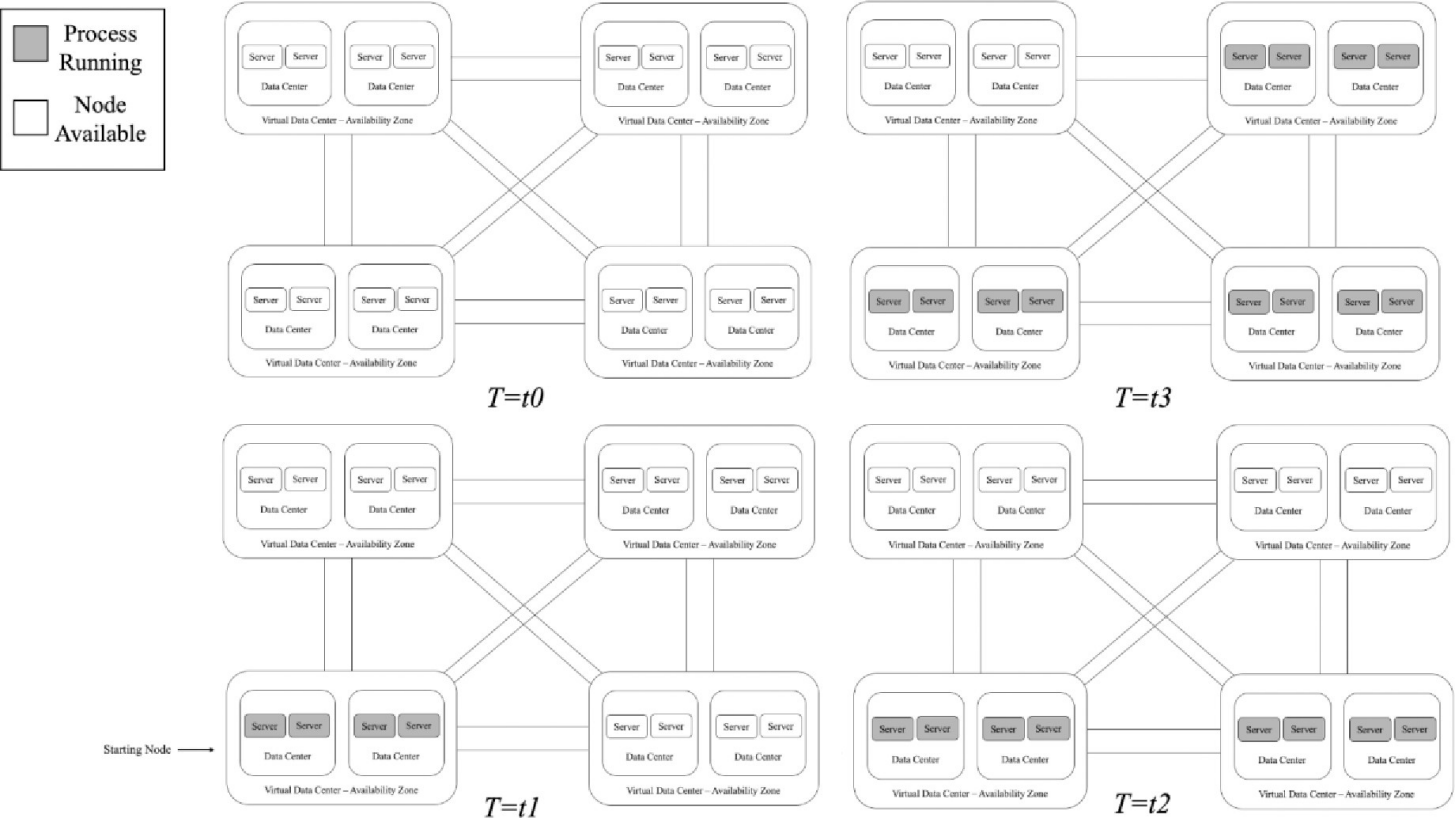
Computing provider’s distributed over a network of machines with the respective published service’s and available nodes.

An orchestration job has to deploy efficiently S types of services, process or tasks in many different computing resources such as a cluster of containers or a pool of (physical or virtual) machine nodes connected over a distributed network with different weights (or costs) between each pair of nodes. This job is a process that needs to run on all M (unique) resources points. The cost to use each node can be defined as the round-trip latency between the nodes in the network or the financial cost associated to the proportional quantitative utilization rate for each resource in a given time period. As more Computational Capacity is added, choosing the shortest route to multiple target nodes will be more computationally complex. Fig 2 illustrate a service orchestration across a set of distributed clusters of machines connected over a common network.

## Materials and methods

### The traveling salesman problem

was introduced by William Hamilton and Thomas Kirkman. It also known as the *messenger problem*. The problem asks given a list of cities and the distance between each pair of nodes what is the shortest route that visits all cities exactly once and returns to the original city at the end. There are several researches dedicated to address this routing problem and it has applications in mathematics, computer science, statistics and logistics.

The computational complexity of an algorithm shows how much resources are required to apply an algorithm such as how much time and memory are required by a Turing machine to complete execution and can be interpreted as a measure of difficulty of computing functions. A measurement of computation complexity is the big *O* notation. It can be defined as: Let two functions *f* and *g* such as *f(n)* is *O(g(n))* if there are positive numbers *c* and N such that

f(n)≤cg(n) Eq 1

and is used to estimate the function growth tax (i.e. asymptotic complexity).

The TSP problem is an important combinatorial optimization problem. As most of the decision problems, it is in the class of NP-hard problems.

**Fig 3:**
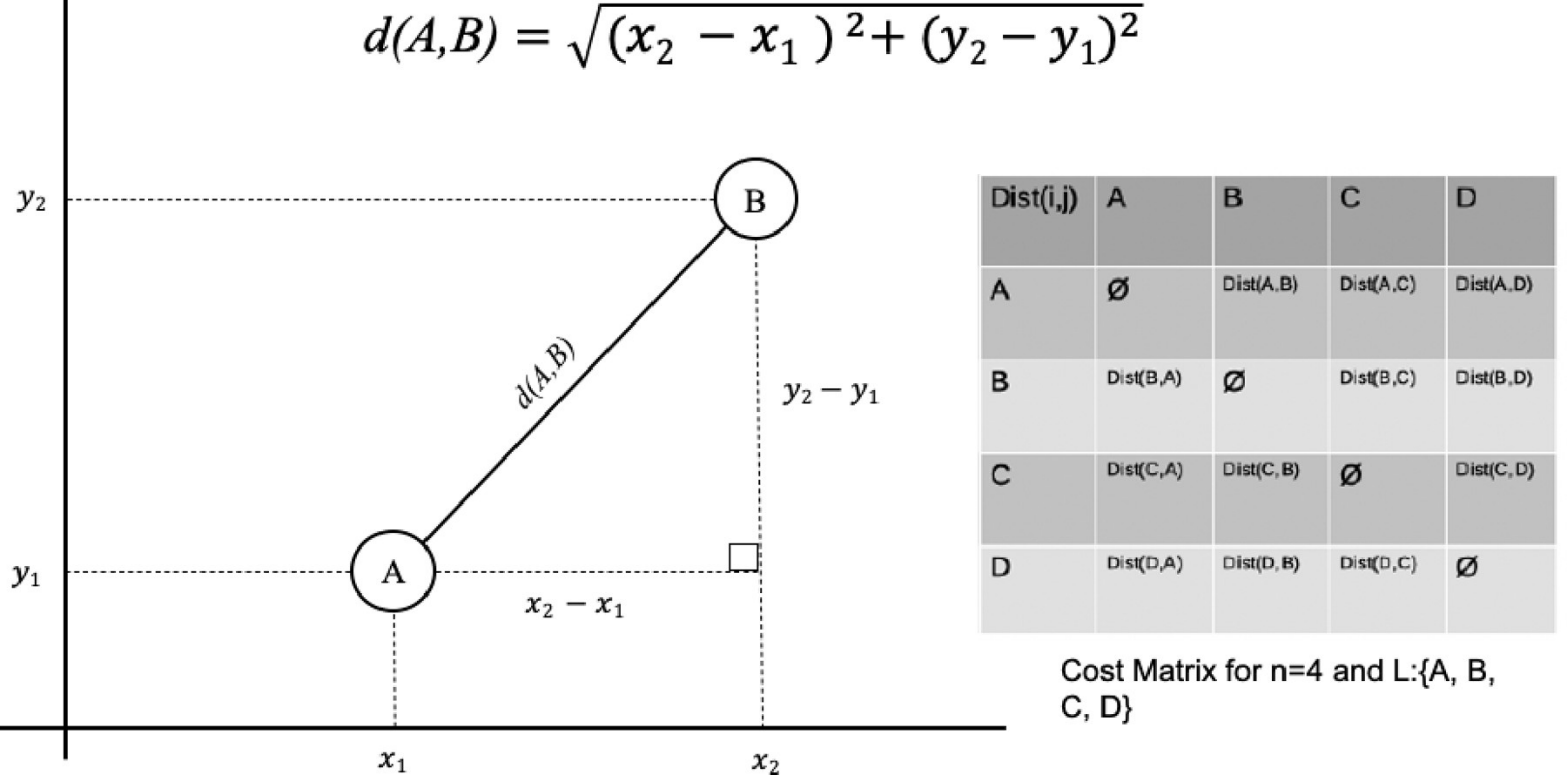
A sample cost matrix for 4 nodes and the Euclidian distance between each pair of nodes.

Consider a salesman traveling from city to city and some of them are connected. His goal is to visit each city exactly once and go back to the first city when he finishes. The salesman can choose any path as long as its valid (i.e. visit each city once and finish at the city it has started the tour) he also wants to minimize his cost by taking the shortest route. This problem can be described as a weighted graph G where each city is a node (or vertex) and is connected by a weighted edge only if the two cities are connected by any kind of road, and this road do not cross any other city. The utility function in the TSP is the Euclidian distance. Fig 3 demonstrate the cost matrix between each pair of nodes of a set defined by valid (non-repeating) permutations in a language L with symbols {A, B, C, D}. The cost/weight between points is calculated as a Euclidian distance in a 2D graph.

Table 1 Demonstrate a sample of valid and invalid strings created from L.

**Table 1:** Solution sample of valid and invalid candidates for a given string schema.

Word *w* in Language *L*	Path Sequence for *L*: *{A*, *B*, *C*, *D*, *E}*	Is Hamiltonian?
Valid	A > B > C > D > E > A	Yes
Valid	D > B > C > A > E > D	Yes
Valid	D > B > A > C > E > D	Yes
Invalid	A > B > A > D > E > A	No

The graph can be represented as a matrix where each cell value is defined as the respective cost (distance) *w* between nodes *v* and *u*. For N nodes the distance matrix is defined as *D = w(v*,*u)* for all (unique) pair of N. The goal of the TSP is to find a permutation π that minimizes the distance between nodes. For symmetric instances the distance between two nodes in the graph is the same in each direction, forming an undirected graph. For asymmetric instances the weights for the edges between nodes can be dynamic or non-existent.

The weigh value of the edge is defined as the distance of the tour (roads) between cities.

For symmetric TSP, as the number of nodes (or cities) increases in the graph G, the number of possible tours growth choice also increase and is factorial. If we consider N nodes, the function of the input size is

f=((N–1)!/2) Eq 2

This is the number of elements (states) an algorithm must evaluate to decide (to halt) the problem and is very large thus requiring considerable time and computational resources even for small instances of the problem. A strategy to address this limitation is to accept near-optimal solutions by setting constrains in the problem using heuristics methods to generate suboptimal approximations. Algorithms such as 2opt, GA, AC and SA define *a*-*priori* knowledge about the distribution of the solution space and then repetitively try to improve the quality. It works by following some heuristic function schema while trying to avoid a local minimum. As the heuristics for TSP and NP problems in general are a best effort strategy to find a good near-optimal solution (by enforcing space and time boundaries), it does not guarantee that the solution found is the best candidate to the problem and therefore an program can never be sure that if by running more time the overall solution cost could be improved, unless the entire solution space to the problem is evaluated. This limitation is set by the definition of NP-hard class.

### Computational complexity theory

Problems in the NP class can be solved by a non-deterministic polynomial algorithm. Any given class of algorithms such as P, NP, coNP, regular etc. must have a lower bound that index the best performance any problem in the class can have. This bound can be described as the total amount of input items (or symbols) a machine must process before halting, and the respective output items produced following a Probability Distribution Function and a given finite Alphabet. A strategy to find a solution to the decision problem is to find a function that reduce or transform a problem from a domain in which there is no know solution to a constrained domain with a known solution.

This allows the algorithm to search the solution space and decide if any solution is a valid (yes-instances) or invalid (no-instances) and its computable by a polynomial-time algorithm.

This strategy allows us to map instances of the Hamiltonian circle problem to a decision version of the Traveling Salesman Problem and can be described as a decision problem to determine if exists a Hamiltonian circuit in a given complete graph with positive integer weights hose length is not greater than a given positive integer m. Each valid (yes-instance) in the TSP problem is mapped to a valid instance in the Hamiltonian problem space and this transformation can be done in polynomial time.

**Fig 4:**
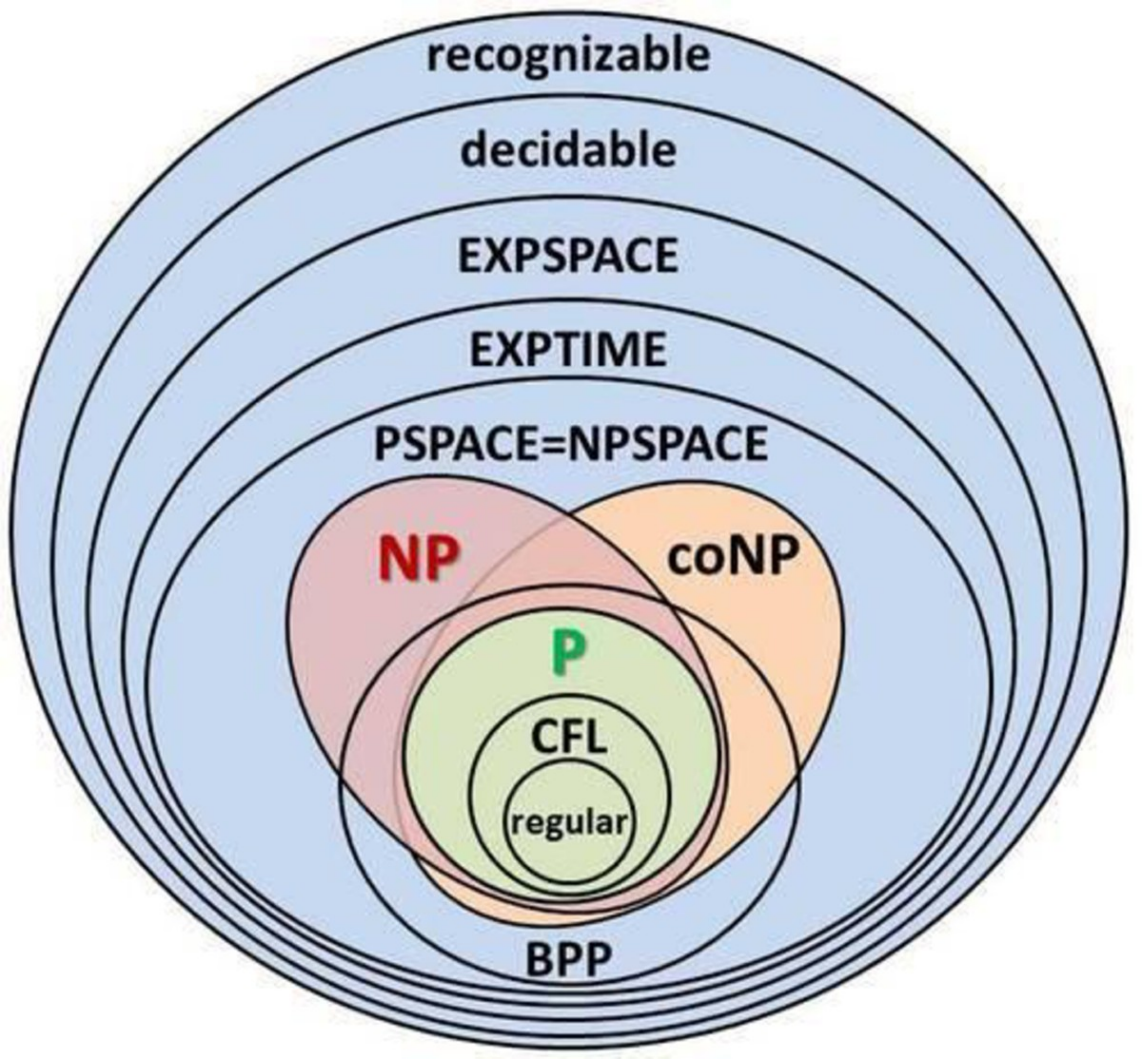
Diagram representation for the many categories of computational complexity.

In Fig 4 reproduced from [2] we have a visual representation of computational complexities categories. The figure demonstrates the groups of Regular, P, coNP, NP, etc class of recognizable problems. In complexity theory, the class P contains all decision problems solvable by a deterministic Turing Machine in polynomial time. The Nondeterministic Polynomial time (NP) class of problems is a category of decision problems that is solvable in polynomial time by a non-deterministic Turing Machine.

The coNP are the class of problems that have a polynomial-time algorithm mapping for “no-instance” solutions which can be used to verify that the proposed solution is valid but there is no such mapping for “yes-instances”. The P class is a subset of both coNP and NP. The bounded-error probabilistic polynomial time (BPP) is a class of problems solved by a probabilistic Turing Machine in polynomial time with an attached probability distribution function with a given error degree. BPP can be interpreted as the complexity class P with a randomness boundary factor.

### Hamiltonian graph

A Hamiltonian cycle (or circuit) can described as a "path" that contains all nodes and the elements in this set are not repeated, with exception to the final vertex. This means that a Hamiltonian cycle in G with start node v has all other nodes exactly once and them finishes at node v. A graph G is Hamiltonian if it has a Hamiltonian cycle. A Hamiltonian cycle with minimum weight is an optimal circuit and therefore is the shortest tour in the TSP Problem.

**Fig 5:**
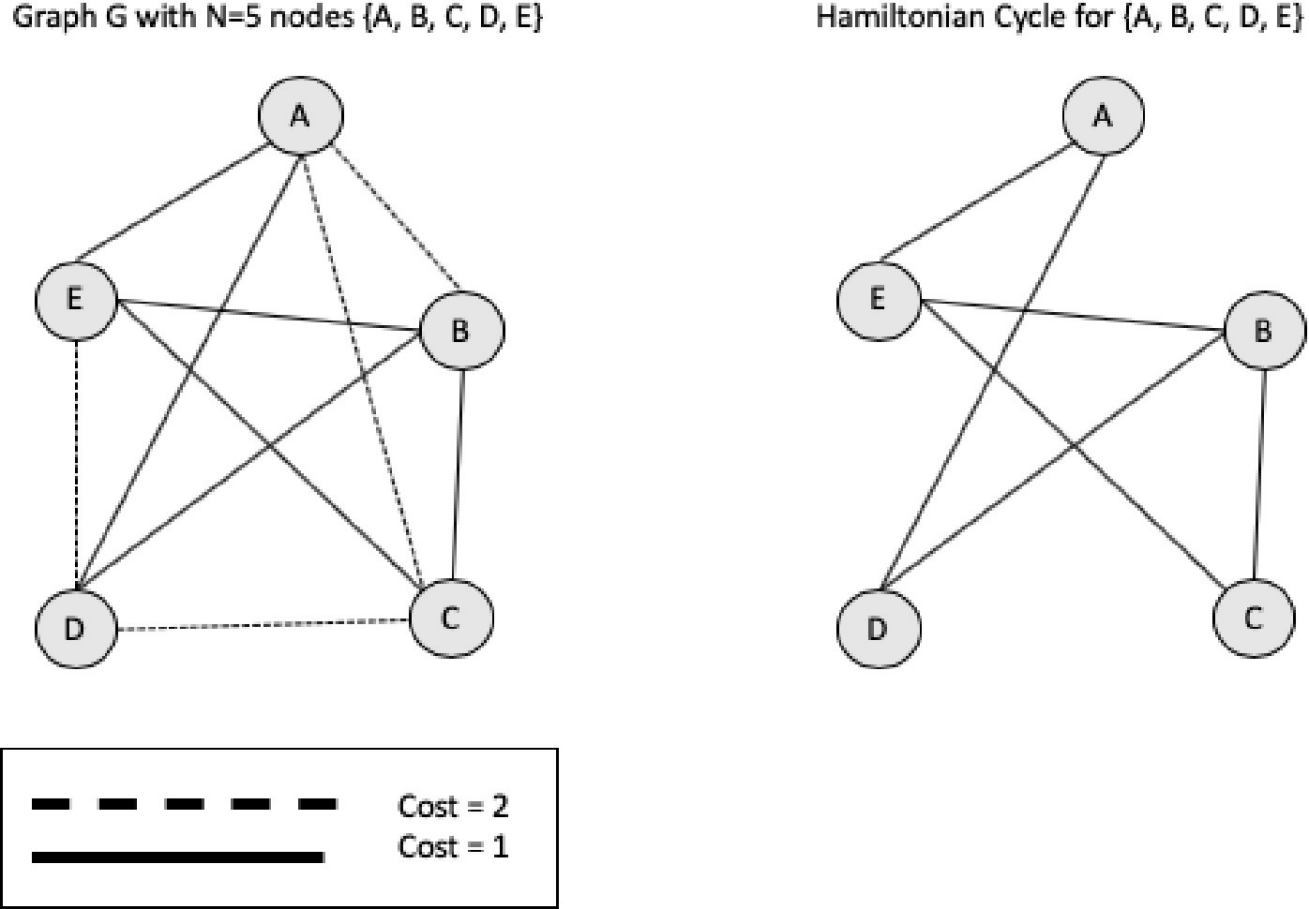
Hamiltonian cycle from a super graph.

The Fig 5 provides an example of Hamiltonian circuit for a Graph G with 5 nodes {A, B, C, D, E}. The Table 2 shows the cost matrix for the super graph G.

**Table 2:** Cost matrix for graph G with 5 nodes.

Cost Matrix	A	B	C	D	E
**A**	0	2	2	1	1
**B**	2	0	1	1	1
**C**	2	1	0	2	1
**D**	1	1	2	0	2
**E**	1	1	1	2	0

Although heuristics methods define special cases for the TSP problem and produces near-optimal solutions with short length (weight) Hamiltonian cycles it does not guarantee that the results are the shortest circuit possible. The algorithms to solve the TSP are grouped in 2 categories: exact (Brute-force, greedy) and approximation algorithms (Heuristics such as Simulated Annealing and Genetic Algorithm).

### Nature inspired models

Researchers have proposed algorithms inspired by natural events and structures like the heating of metals and the growing behavior of biological organisms. Those methods do not iterate over the entire solution space but rather a portion in order to find the local minimum. They start with an initial random solution and tries to improve the solution quality over each interaction until some input Threshold parameter factor T is reached like a maximum number of interactions; maximum number of candidate solutions; no further improvements found after several iterations; the rate of decay in a dependable temperature probabilistic function or a minimum quality threshold is achieved.

Therefore, heuristics approximation methods can be interpreted as a non-deterministic way to address the error rate between the known solutions and the unknown solutions in polynomial time (i.e. Entropy reduction methods). Although such algorithms do not have to traverse the entire solution space it must decide–“outguess” or “bet”—when a random candidate solution with negative gain will be accepted (i.e. candidate with worst solution quality than current know best solution) in the hopes that eventually it would lead to the shortest distance (i.e. a better solution quality).

Nature-inspired models such as Genetic Algorithms (GA), Ant Colony (AC) and Simulated Annealing (SA) use prior information to improve the solution results and thus are biased towards this encoding. Alternatively, by modeling the TSP problem as a communication channel with a probability density function associated with the stochastic process that generates the solutions at random (following a Bernoulli process), thus we can bound the limits of the search space to a log-normal distribution.

The advantage of this method is that by relying on the statistical analysis of the solution space instead of the computational complexity of the problem we can have equal or better quality than the traditional algorithms without relying on computationally complex implementations that have a higher time and space constrains.

Therefore, this paper attempts to provide an algorithm to solve the TSP using for the decision rule the entropy measured for the solution cost distribution H(X) and by maximizing the expected value of the logarithm of cost/weight/distance variable, defined as the utility function g(X). This is equivalent to maximize the expected geometric growth rate.

## Literature review

### Solving hard problems

There are 3 categories of algorithms to solve NP-hard problems such as the TSP:

- **Exact Algorithms**: Fast to converge to a solution only form small instances of the problem.
- **Heuristic Algorithms**: Compromise quality but converge with acceptable time requirements. Produce sub-optimal results.
- **Special cases**: Restrict the boundaries of the solution space domain to a subproblem for which there is an exact (or better) solution quality.

**Exact Algorithms** try all permutations in the domain and verify if each candidate string is the best solution. This algorithm uses a brute-force search method. The running time has a computational complexity of *O(n*!*)*. As the number of nodes increases the complexity (or running time) increases with a factorial growth.

#### Combinatorial problems

Other approaches such as Branch and bound algorithms can be used to optimize combinatorial problems. The branch-and-bound method produces lists of candidate solutions by a search in the state space. The set of states forms a graph. Each sub pair of states is connected if the first and the second state are produced by an operator to transform the first state in the second state.

From **Poole and Mackworth [3]**, there are two categories of state space search algorithms:

1. **Uninformed**: The algorithm does not have any prior information about the state distribution. The Breadth-First Search is an instance of this class.
2. **Heuristic Search**: The algorithm has encoded information about the solution distribution defined by a heuristic function. The A* search algorithm is an instance of this class. The A* is a path finding algorithm and its defined in terms of weighted graphs. It has a running time of *O(b*^*d*^*)*.

### Heuristic algorithms

are method for problem-solving using approximations to find suboptimal solutions, under some predefined degree of freedom. There are many heuristics designed to address the TSP as a combinatorial optimization such as genetic algorithms, simulated annealing, tabu search, ant colony optimization, swarm intelligence and cross-entropy method. There are two class of heuristics: Constructive Methods and Iterative Improvement.

### Constructive heuristics

Starts with an empty solution and expands the current know partial solution at each execution time unit until the target complete solution is found. In the Nearest neighbor (NN) algorithm, the salesman starts at a random city and at each execution time it visits the nearest city (performing a local move) until all nodes have been visited. The method can be optimized by pre-processing (or filtering) the possible candidate solutions to clusters of best-quality arrangements of node distributions in a 2D graph. This optimization method works as a prefix code property. The method has a worst-case performance of *O(N*^*2*^*)*.

#### Nearest Neighbor (NN) and Nearest Insertion (NI)

From Asani, Okeyinka and Adebiyi [4] the Convex-hull and Nearest Neighbor heuristics can be combined. Their results where compared with two benchmark algorithms Nearest Neighbor (NN) and Nearest Insertion (NI). Their experimental results show their approach produces better quality in terms of computation speed and shortest distance.

#### The Christofides and Serdyukov algorithm

Based in graph theory and combines a minimum spanning tree with a minimum-weight perfect matching where the distances between nodes in a super-graph is symmetric and follow the triangle inequality and thus form a metric space. The solution Christofides algorithm has the best worst-case scenario currently know with a quality tour of at most 1.5 the optimal string. The computational complexity is *O(n*^*3*^*)*.

**Fig 6:**
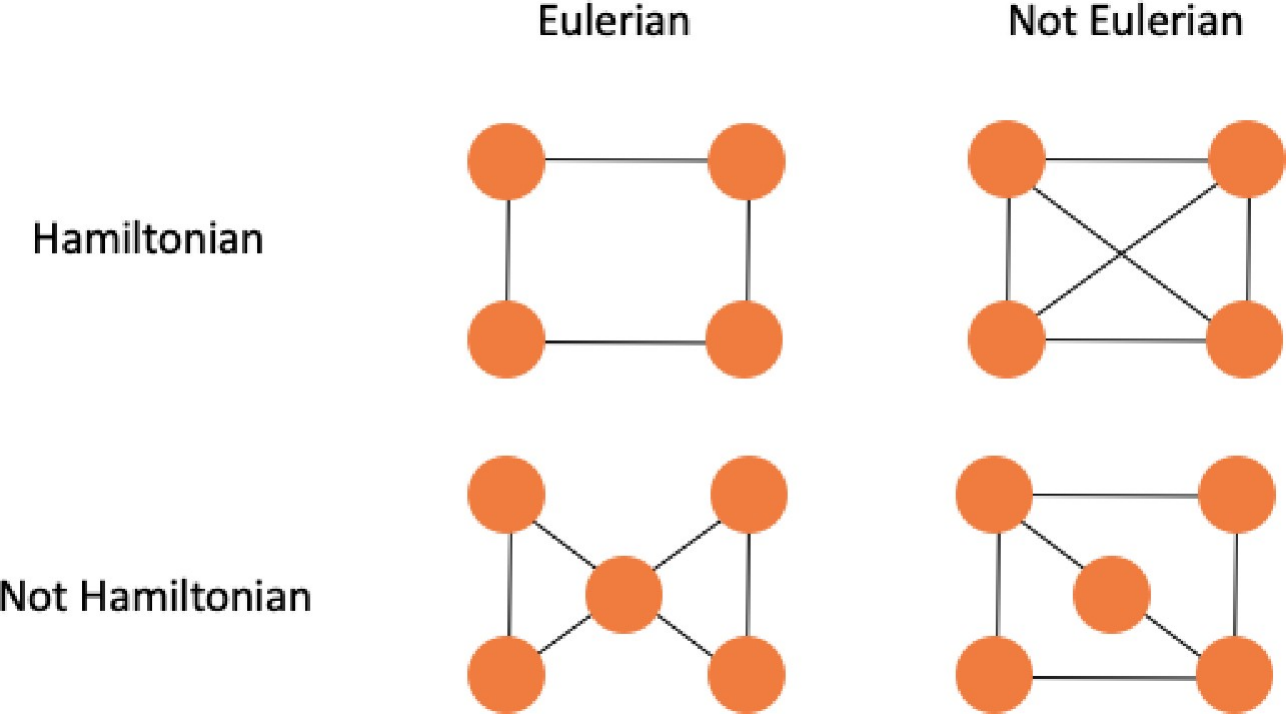
Example of Hamiltonian and Eulerian graphs.

Given a Eulerian path we can find a Eulerian tour in *O(n)* time. The method finds a minimum spanning tree and duplicate all edges to create a Eulerian Graph. A graph *G = (V(G)*, *E(G))* is Eulerian if is both connected and has a closed trail and thus represents a tour with no repeated edges, containing all edges of the graph. In graph theory a Eulerian path is a string in a finite graph that visits every edge exactly once (no repeated edges) and it allows the revisiting of nodes, then returning to the starting vertex. In Fig 6 we have a comparison between Hamiltonian and Eulerian graphs.

The Christofides and Serdyukov algorithm can be described as:

1. Find the minimum spanning tree
2. Create a map for the problem with the set of nodes of odd order
3. Find a Eulerian tour path for the map
4. Convert to the TSP: If a node is visited twice create a shortcut from the node before the current and next node.

### Iterative improvement

Algorithms such as the Pairwise exchange method implemented by the 2-opt algorithm remove two edges at each interaction and reconnected the edges by a new shorter path.

#### k-opt method

The 2-opt and 3-opt are a special case of the k-opt method. The method can be optimized by a preprocessing using the greedy algorithm. For a random input the average running time complexity is *O (n log(n))*.

#### 2opt, k-opt

Croes proposed the 2-opt algorithm [5], a simple local-search heuristic, to solve the optimization problem for the TSP. It works by removing two edges from the tour and reconnects the two paths created. The new path is a valid tour since there is only one way to reconnect the paths. The algorithm continues removing and reconnecting until no further improvements can be found. k-opt implementations are instances of 2-opt function but with *k > 2* and can lead to small improvements in solution quality. However, as *k* increases so does the time to complete execution.

In his work [6] proposed the Tabu Search method and it can be used to improve the performance of several local-search heuristics such as 2opt. As neighborhood searches algorithms like 2opt can sometimes converge to a local optimum, the Tabu search keeps a list of illegal moves to prevent solutions that provide negative gain to be chosen frequently. In 2opt the two edges removed are inserted in the Tabu list. If the same pair of edges are created again by the 2opt move, they are considered Tabu. The pair is kept in the list until its pruned or it improves the best tour. However, using Tabu searches increases computational complexity to *O(n*^*3*^*)*, as additional computation is required to insert and evaluate the elements in the list.

**Fig 7:**
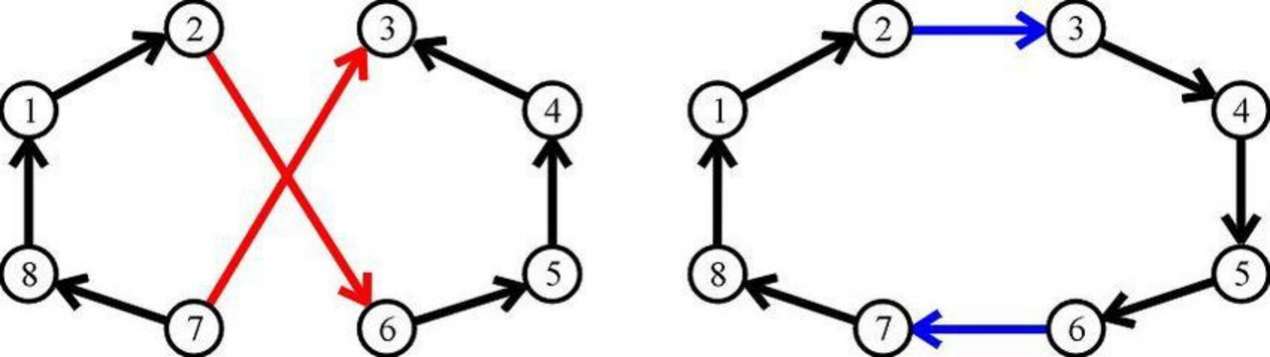
Generating 2-opt moves.

The Fig 7 show the 2-opt moves from [7].

[8] compared several heuristic strategies for the TSP problem such as Greedy, Insertion, SA, GA, etc. He investigated the performance tradeoff between solution quality and computational time. He classifies the heuristics in two class: Tour construction algorithms and Tour Improvement algorithms. All algorithms in the first group stops when a solution is found such as brute-force and Greedy Algorithm. In the second group, after a solution is found by some heuristics, it tries to improve that solution (up to a certain computation and/or time constraints) such as implemented by 2opt, Genetic Algorithm and Simulated Annealing. He concluded by showing that the computational time required is proportional to the desired solution quality.

#### Simulated Annealing (SA)

Simulated Annealing are heuristics with explicit rules to avoid local minimal. It can be described as a local random search that temporarily accepts moves with negative gain (i.e. were produced by solutions with worst quality than current). These methods simulate the behavior of the cooling process of metals into a minimum energy crystalline structure.

**Fig 8:**
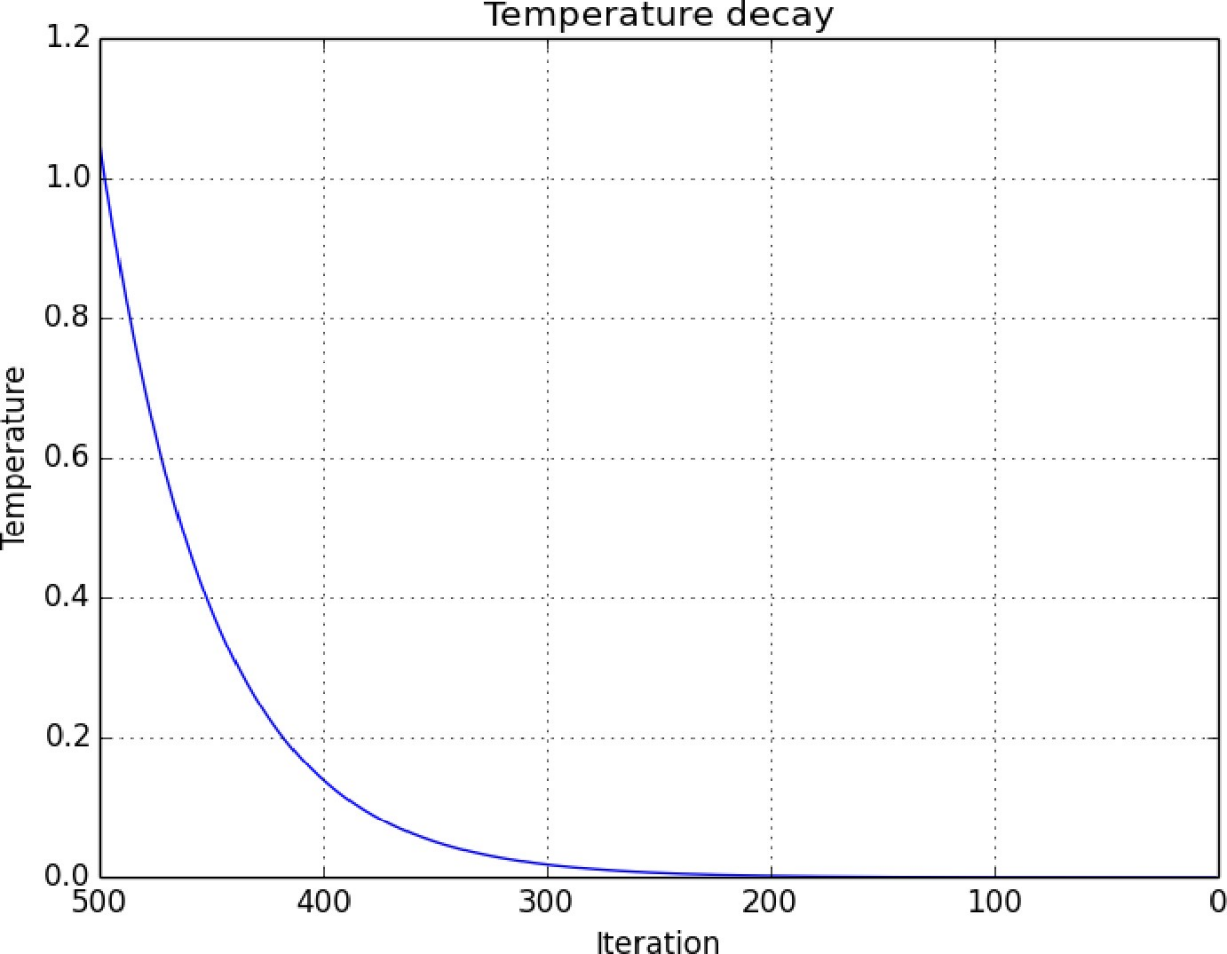
Temperature decay relative to the iterations of the SA algorithm.

This concept is analogous to the search of global maximum and minimum. The probability of accepting a solution is set by a dependable probability function of a temperature parameter variable *t*. As the temperature decreases over time the probability changes accordingly. Fig 8 demonstrates the simulated decay in the temperature function over the number of interactions in an algorithm.

The acceptance probability is defined as p(x) = 1 if f (y) ≤ f (x) and when otherwise

$$p\left(x\right)=e^{-\left(f\left(y\right)–f\left(x\right)\right)/t)}$$ Eq 3

where *t* is the input temperature.

[9] research combines the Simulated Annealing method with the Gene Expression Programing to improve solution diversification and state search. Their results show a better performance that other methods such as ant-colony, naive SA, naive GA, etc.

The SA algorithm specifies the neighborhood structure and the cooling function.

Fig 9 from [9] represents the SA algorithm flowchart.

**Fig 9:**
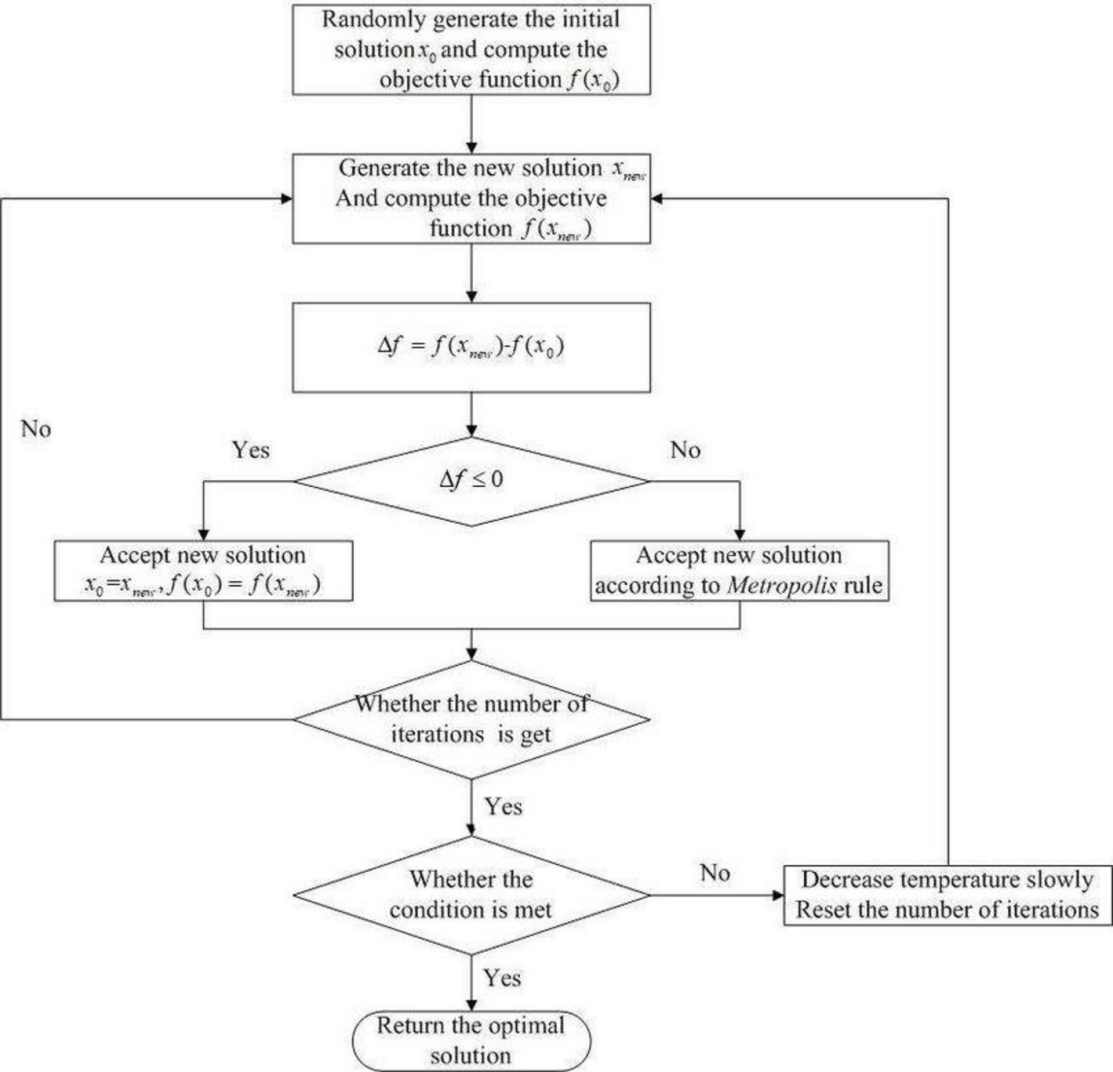
Simulated annealing algorithm flowchart.

[10] research explores the optimization technique in Simulated Annealing and the solution impact by different temperature schedule designs. It concludes that the quality of finite time SA implementations is a result of relaxations dynamics similar to the analogy with the vitrification process of amorphous materials (non-crystalline) or disordered systems (high entropy).

Their results can be generalized in terms of entropy distributions. By the thermodynamics analogy, we know the position of particles in higher temperatures have a higher degree of uncertainty and thus there is more entropy as each particle is bouncing randomly. As the temperature decreases the particles forms bounds between them and the overall state distribution forms a glassy or crystal structure. As there is less uncertainty about the average state value for each particle there is less entropy and thus more information about the optimal state distribution is known.

[11] work provide a numerical analysis of the simulated annealing applied to the TSP. The cost distribution is compared to the control parameter of the cooling method. It concludes that the average-case performance can be defined by assuming the deviation between the final total cost and the optimal solution is distributed by a gamma distribution. This behavior is also observed in our research and this model is explained by the Kolmogorov Complexity for the Bernoulli string that represents a random solution candidate. The entropy for this distribution can be calculated and is the maximum entropy probability distribution. This is the sufficient statistics needed to represent the state set.

#### Metropolis algorithm and heuristic optimizations

Let *f(X)* be a function with output proportional to a given target distribution function *r*. The function *r* is the proposal density. At each iteration of the algorithm it attempts to move around the sample space. For each move it decides sometimes to accept a given random solution or stay in place. The probability of the solution of the new proposed candidate is with respect to the current know best solution. If the proposed solution is more probable than the know existing point, we automatically accept the new move. Else if the new proposed solution is less probable, we will sometimes reject the move and the more the decrease the probability, less likely we will accept the new move. Most of the values returned will be around the *P(X)* but eventually solutions with lower probability will be accepted. This characterizes can be interpreted as a generalization of the methods proposed by Simulated Annealing and Genetic Algorithms.

Other heuristics such as 2-opt, 3-opt, inverse, swap methods can be used to generate candidate solutions. Several researches such as [8] have been made to study the performance of different SA operators to solve the TSP problem [12]. proposed a list-based SA algorithm using a list-based cooling method to dynamically adjust the temperature decreasing rate. This adaptive approach is more robust to changes in the input parameters. Their research provide an improved Simulated Annealing method that uses a dynamic list to simplify the parameter settings. This method is used to control the cooling rate for the decrease temperature used by the Metropolis Rule. The list is updated iteratively following the solution space topology. This cooling schedule can be defined as a special geometric cooling method with variable coefficients. This characteristic gives the algorithm more resistance to different input parameters values while producing good sub-optimal results.

In his work [13] proposed a biological inspired bee system to optimize the routing in Railway systems. They conclude that the average solution results are better or equivalent than the traditional SA and GA methods.

The quality of the solution can be improved by allowing more time for the algorithm to run. [14] observed that the performance of 2-opt and 3-opt algorithms can be improved by keeping an ordered list of the closest neighbors for each city-node and thus reducing the amount of solutions to search but requiring more memory to keep the list of states.

#### Genetic Algorithm (GA)

Genetic Algorithms was first introduced by [15] based on natural selection theory, as a stochastic optimization method in random searches for good (near-optimal) solutions. This approach is analogous to the "survival of the fittest" principle presented by Darwin. This means that individuals that are fitter to the environment are more likely to survive and pass their genetic information features to the next generation.

**Fig 10:**
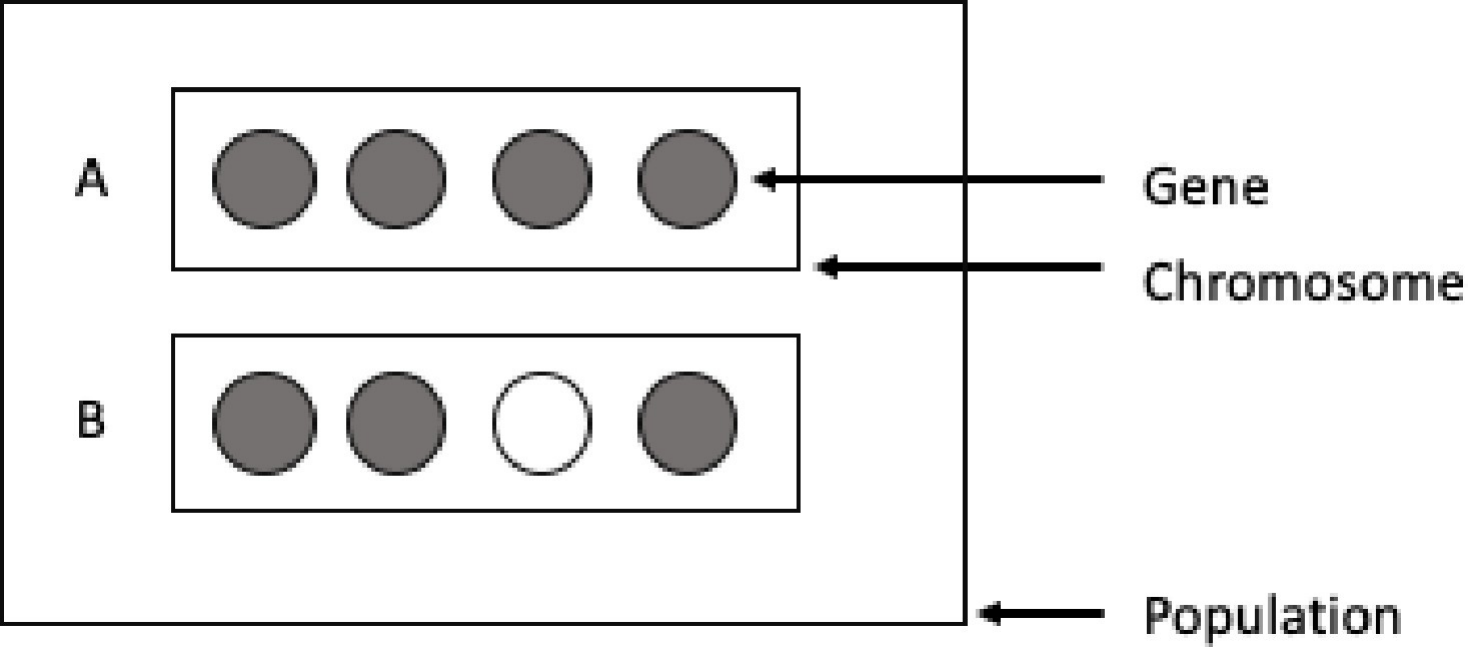
Chromosome for a sample of individual candidate solutions.

In TSP the chromosome that models a solution is represented by a "path" in the graph between cities. GA has three basic operations: Selection, Crossover and Mutation. In the Selection method the candidate individuals are chosen for the production of the next generation by following some fittest function In the TSP This function can be defined as the length (weight) of the candidate solutions tour. In Fig 10 we have a representation of genes and chromosomes.

**Fig 11:**
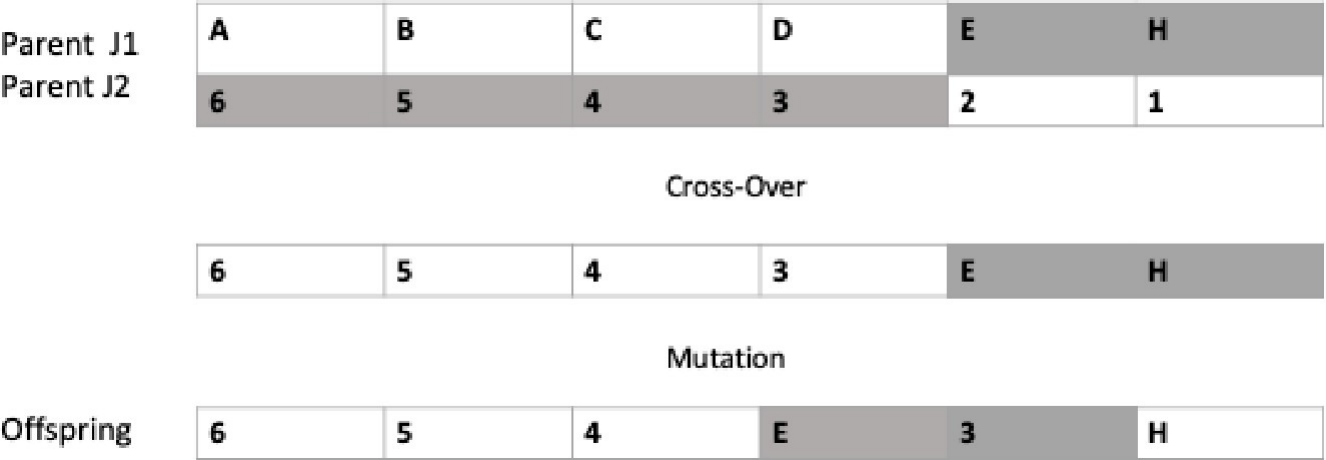
Offspring representation for the genetic algorithm mutation and crossover operators.

In Fig 11 is demonstrated an example of two parents under the Mutation and Crossover operators to generate a new offspring.

Next those individuals are chosen to mate (reproduction) to produce the new offspring. Individuals that produce better solutions are more fit and therefore have more chances of having offspring. However, individuals that produces worst solutions should not be discarded since they have a probability to improve solution in the future. In other words, the heuristic accepts solutions with negative gain hoping that eventually it may lead to a better solution.

Several researches have studied the performance trade off of selection strategy and how the input parameters affect the quality of solution and the computational time [16]. in his work explores different selection strategies to solve the TSP and compare the performances quality and the number of generations required. It concludes that tournament selection is more appropriate for small instance problems and rank-based roulette wheel can be used to solve large size problems.

[17] compared the quality of the solution and the convergence time on many selection methods such as proportional, tournament and ranking. They conclude that ranking and tournament have produced better results that proportional selection, under certain conditions to convergence. In his work [18] explored proportional roulette wheel and tournament method. He concluded tournament selection is more efficient than proportional roulette selection. Their work presents a simple genetic algorithm that combines roulette wheel and tournament selection. Their results suggest that this approach converges faster than roulette wheel selection.

This conclusion is related to the findings in our research and can be explained by Information Theory. The tournament selections mechanism helps to avoid the algorithm to waste execution iterations with solutions that have more noise (i.e. local minimum candidates–suboptimal solutions) and also by providing more useful information by giving a chance for all candidate to eventually produce optimal solutions, through the roulette wheel method.

**Fig 12:**
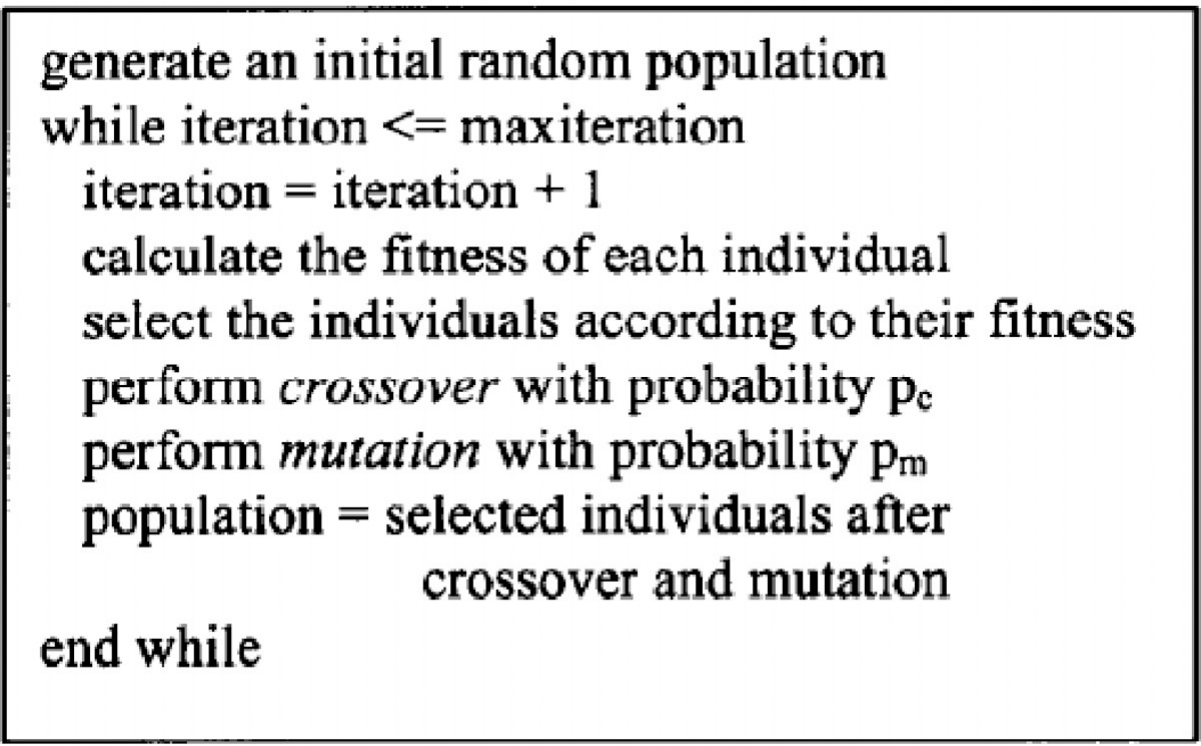
Basic genetic algorithm.

The Fig 12 contains the pseudo-code for a Genetic Algorithm from [19].

#### Ant Colony (AC) optimization

**Ant colony (AC) optimization** is a method that models the behavior of ants to find the shortest route in the nest. The choice for a given path by each ant is defined by the distribution of pheromones left by other ants when in transit.

The method is described as:

1. Initialize: Create initial distribution of pheromone in the region
2. For *i* from *(0*, *n)*
  - For each ant
    - Evaluate the objective function
    - Update know best solution tour
    - Update pheromone intensity distribution
  - Simulate decay pheromone intensity
3. Stop Criterion Achieved?
  - Yes: Local minimum found
  - Continue search until maximum iteration n is reached.

In the work *“Ant supervised by PSO and 2-Opt algorithm*, *AS-PSO-2Opt*, *applied to Traveling Salesman Problem”* from Kefi, Rokbani, Krömer and Alimi [20] there is a optimization of the 2-opt method using a post-processing for the solution paths to help avoid local minimum. Their results perform better than other benchmark algorithms such as Genetic Algorithm and Neural Networks.

**Fig 13:**
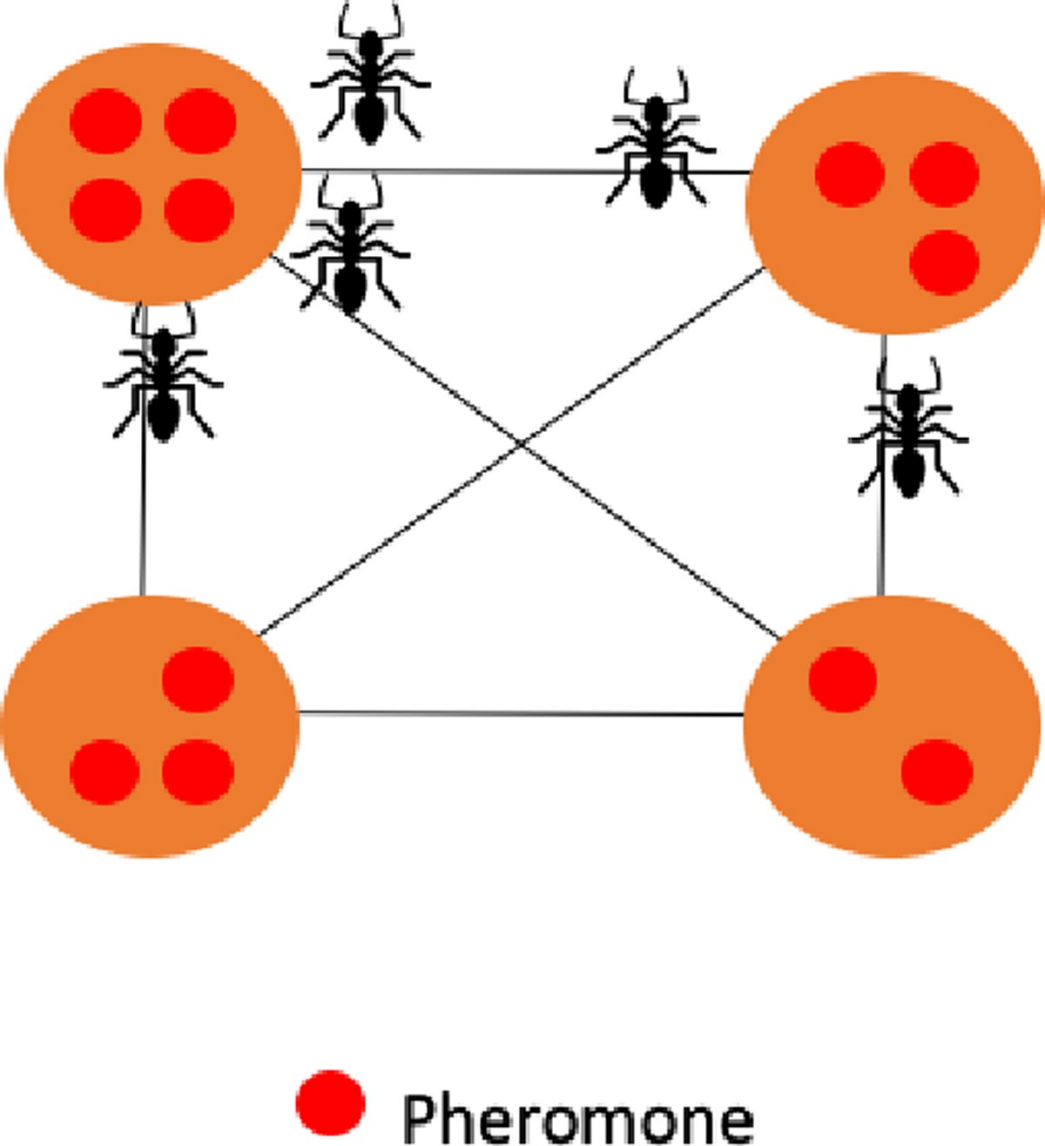
Ant colony pheromone representation.

In Fig 13 we have a representation of ant nest and pheromone distribution across a 2D region.

#### Cross-entropy (CE) method

Is a combinatorial optimization method with noise. The method approximates the optimal utility function estimator with two targets:

1. Create a sample from a probability density function.
2. Minimize the cross-entropy between the distribution and the density function to improve the candidate solution quality for the next iteration.

The CE algorithm can be described as:

1. Init: Set parameter μ as average and σ as the standard deviation
2. For *i* in (0, N)
  - Create random sample S with size *n* using a normal distribution *N (μ*, *σ)* with parameters μ and σ
  - Evaluate the Noise (entropy) distribution for the sample S
  - Select the best Z% candidates of the solution sample to form a new subset T ⊆ S
  - Update μ and σ with the probability distribution function of T, assuming a normal distribution

**Fig 14:**
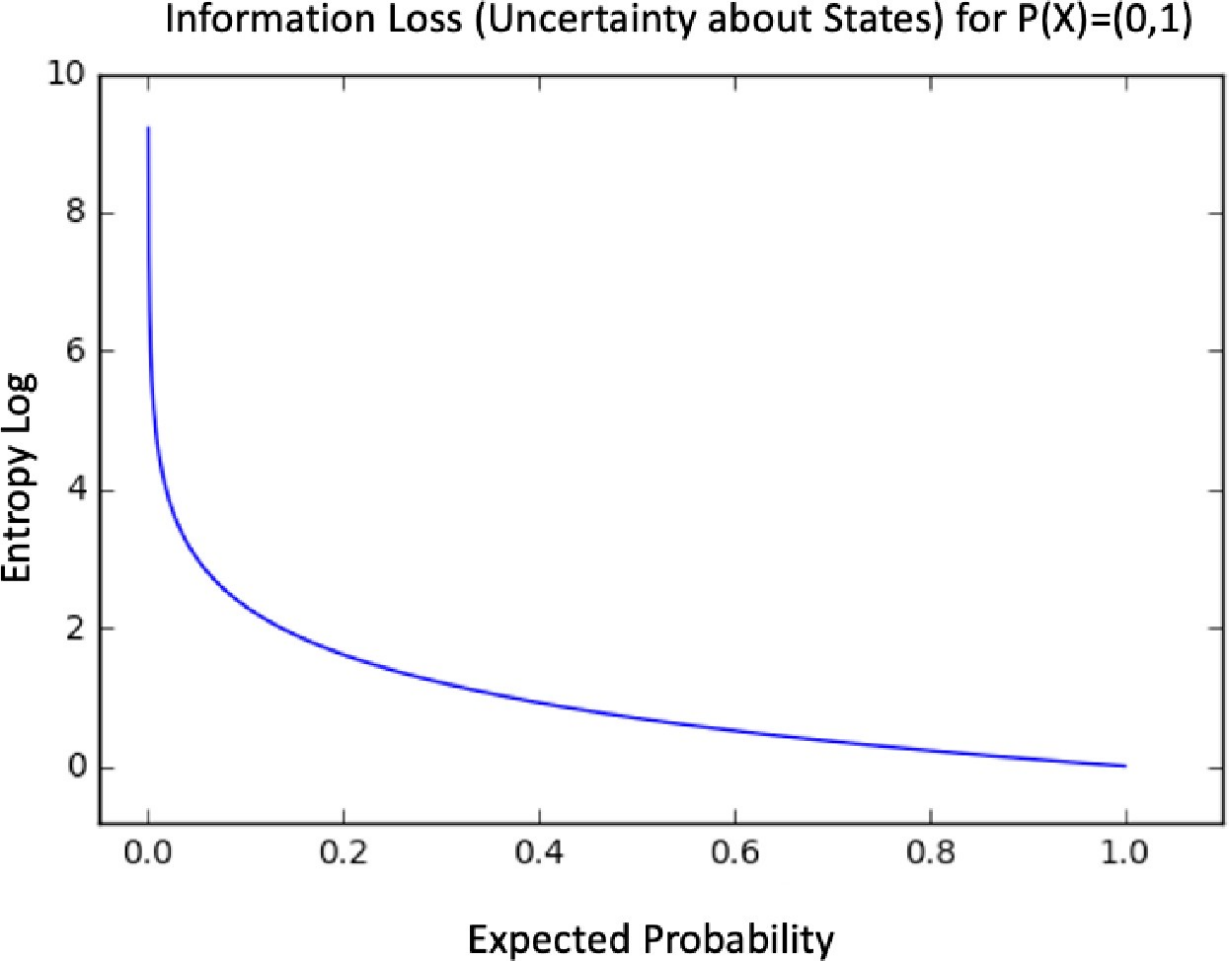
Entropy decrease process.

The algorithm works by reducing the entropy using a maximum like hood estimator *P(X)*. In Fig 14 we have a representation for the entropy function *P(X)*.

In “Solve Constrained Minimum Spanning Tree By cross-entropy (CE) method” [21] propose a parallel, randomized method to find a spanning tree using the lowest total cost relative to the cost weight under constrained weight boundaries.

**Special cases** are a class of algorithms that restricts the limits in the problem to find the optimal solution inside a give boundary. A metric TSP defines the distance between nodes under the triangle inequality and replaces the Real value Euclidian for the Manhattan distance. The Euclidian TSP is a special case for the metric TSP using integer numbers for the Euclidian distance.

The Euclidian TSP has a Euclidian minimum spanning tree associated with the minimum spanning tree of the graph and has the expected running time complexity *O(n log n)*.

## Proposed method

### Background theory review

#### Information Theory (IT)

Quantifies the amount of information in a noisy communication channel and is measured in bits of entropy. IT is based in probability theory and statistical distributions. Entropy quantifies the amount of uncertainty in a random Bernoulli variable created by a Bernoulli process thus information can be interpreted as a reduction in the overall uncertainty about a set of finite states. Mutual information is a measure of common information between two random variables and it can be used to maximize the amount of information shared between encoded (sent) and decoded (received) signals. In Table 3 we have the relationship between Information and Entropy. As we increase our knowledge about the states following a probabilistic function distribution, we reduce entropy, as there is less uncertainty about possible state outcomes.

**Table 3:** Relation between the level of uncertainty and knowledge about possible string outcomes.

Information	Entropy	Word	P (X = 0)	P (X = 1)	E(X)
High	Low	0000	1	0	1*1*1*1 = 1
Medium	Medium	0001	0.75	0.25	0.75*0.75*0.75*0.25 = 0.105
Low	High	0011	0.5	0.5	0.5*0.5*0.5*0.5 = 0.0625

#### Information theory as an approximation method

Information Theory has applications in a range of fields and is used as a mathematical framework for encoding and decoding of information such as in adaptive systems, artificial intelligence, complex systems, network theory, coding theory, etc. IT quantifies the number of bits required to describe a given a set of states using a statistical distribution function for the input data.

#### Entropy of a random sequence

**Fig 15:**
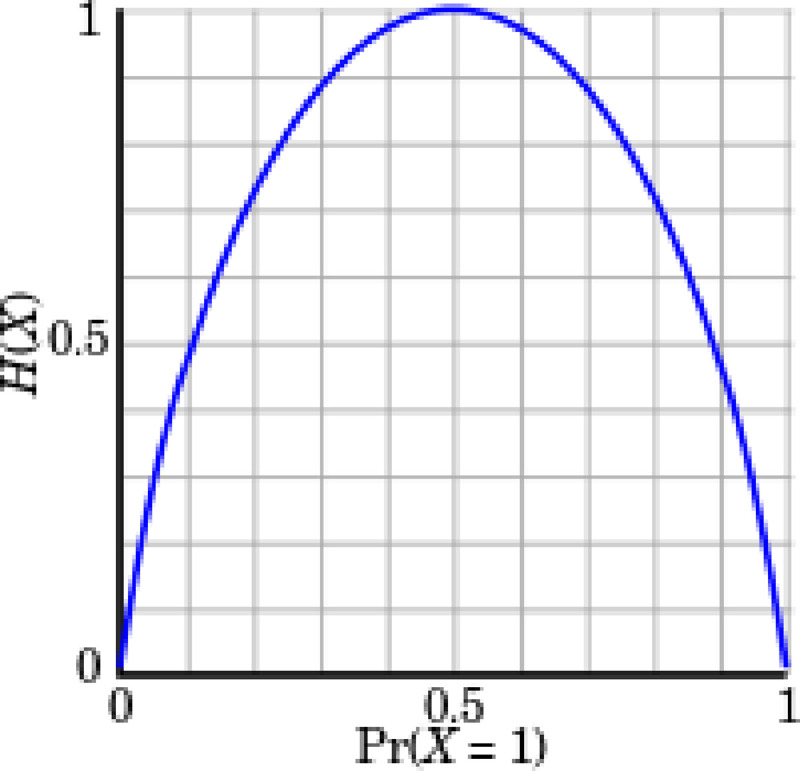
Entropy H(X) vs Probality Pr(X = 1).

Entropy is a measure of uncertainty of a random variable. It is the average rate at which information is produced by a stochastic process [22]. defined the entropy H as a discrete random variable X with possible values as outcomes draw from a probability density function P(X). Fig 15 demonstrate the variation in entropy H(X) vs a Bernoulli distribution. In Eq 4 the entropy function is defined as:

$$H(X)=-\sum_{i=1}^{n}P(x_{i})\log_{b}P(x_{i})$$ Eq 4

#### Random variables and utility function

Let X be an independent random variable with alphabet *L*: *{001*, *010*, *100*–*}*. A utility function *g* is used to model worth or value and is defined by *g*: *{X ⊂ ℝ}* and it represents a preference of relation between states. The utility function *Y = g(X)* of a random variable *X* express the preference of a given order of possible values of *X*. This order can be a logical evaluation of the value against a given threshold or constant parameter. The g(X) is defined by a normal distribution with given mean and variance under some degrees of freedom (i.e. confidence level).

As an example if *g(X1 = 001 = 1) = c1 and g(X2 = 010 = 2) = c2* are the costs of two routes between a set of nodes in a super-graph G*, we can use this function to determine the arithmetical and logical relationship between them and decide if c1 is worst, better, less, greater or equal to c2. Therefore *g(X1) < g(X2)*. The probability density function pdf(Y) can be used to calculate the entropy of the distribution of the cost values. An exponential utility is a special case used to model when uncertainty (or risks) in the outcome between binary states and in this case the expected utility function is maximized depending on the degree of risk preference.

**Fig 16:**
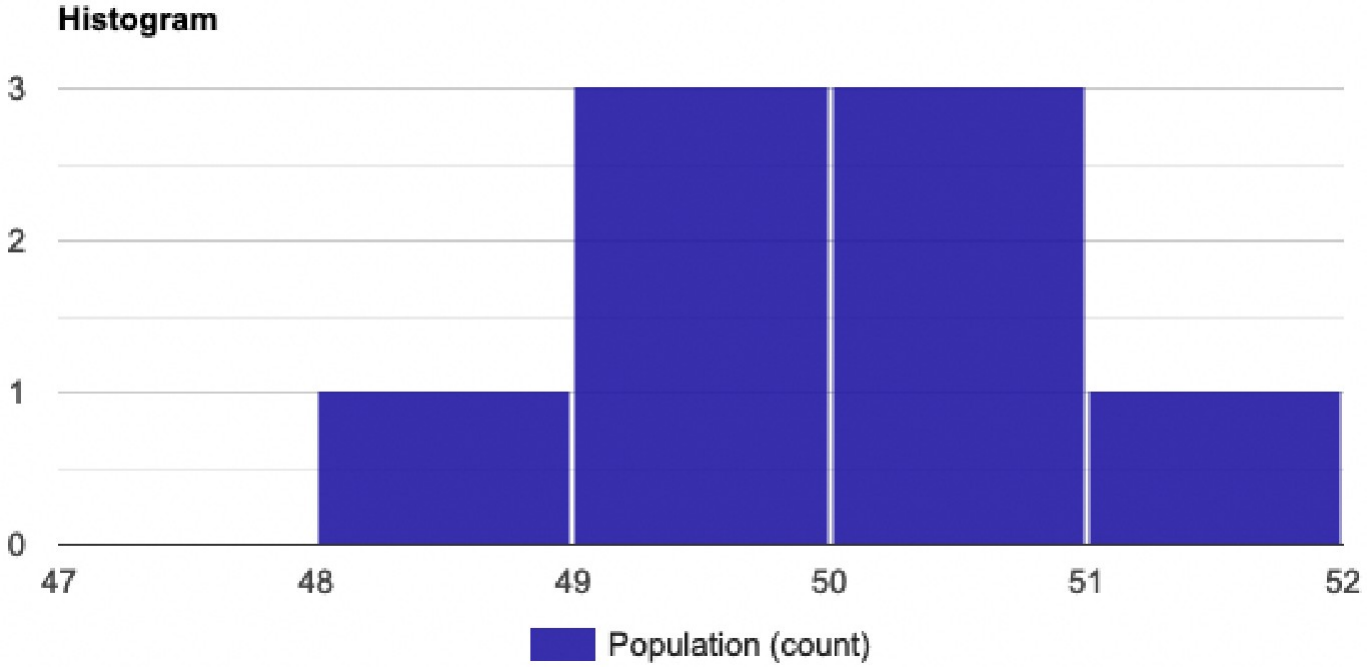
The probability density function for cost function g(X).

**Fig 17:**
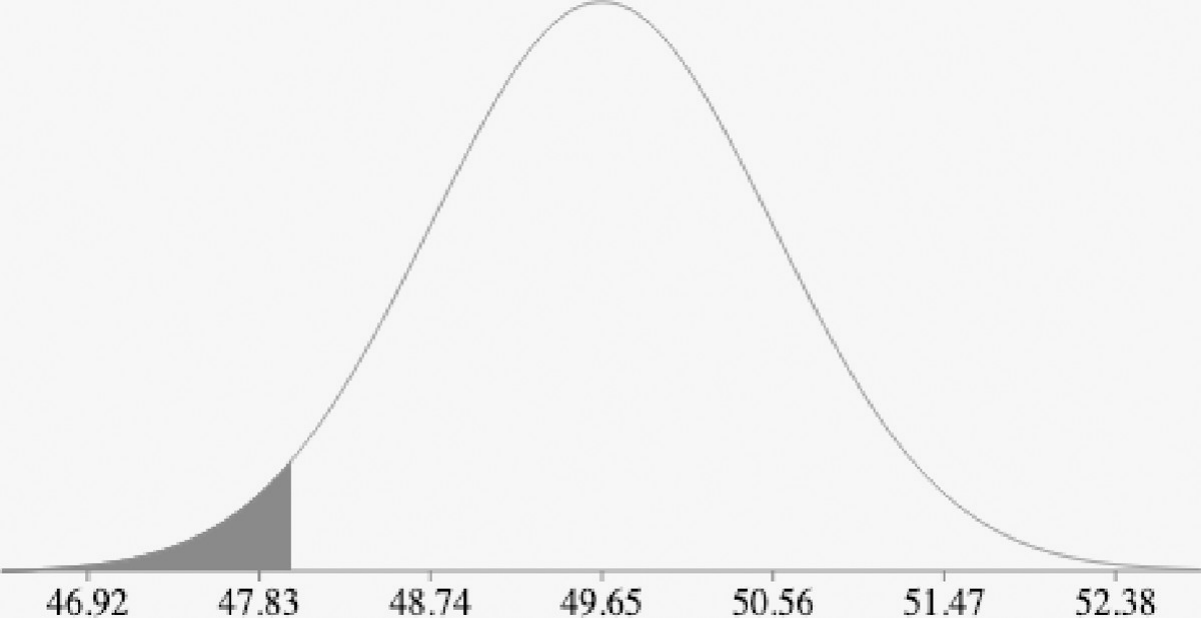
The normal distribution with mean 49.65 and standard deviation 0.91. Then P (X<48; X < min g(X)) = 0.0349.

Fig 17 demonstrate an example of a normal distribution for cost function *g(X) = {50*, *51*, *49*, *49*.*3*, *50*.*5*, *50*.*3*, *49*.*1*, *48}* and the probability *P(X<48)*. Fig 16 shows the histogram for *g(X)*. Table 4 demonstrate the calculation for the mean, standard deviation and variance for *g(X)*, thus we have:

**Table 4:** Statistical metrics for function g(X) distribution.

Parameters	Output
Standard Deviation, σ	0.91241437954473
Count, N	8
Sum, Σx	397.2
Mean, μ	49.65
Variance, σ^2^	0.8325

#### Kolmogorov complexity

The Kolmogorov complexity [23] of a string *w* from language *L* denoted by

$$K_{c}\left(w\right):\;p\left(L\right)=w$$ Eq 5

is the shortest program from alphabet *L* which produces *w* as output and halts. The conditional Kolmogorov complexity of string *x* relative to word *w* is defined by

$$K_{x}\left(x|w\right):\;p\left(x\right)=w$$ Eq 6

and is the length of the shortest program that receives *x* as input and produces *w* as output.

#### Complexity of a string and shortest description length

Let *U* be a Universal computer (i.e. Universal Turing Machine). If description *d(x)* is the minimal string to encode *x*. Kolmogorov complexity *K*_*c*_*(x)* of a string *x* of a computer *U* is

$$K_{c}\left(x\right)=\left|d\left(x\right)\right|$$ Eq 7

It is the minimum length program p that output variable *x* and halts. Assuming the program is defined by

p:U(p)=x Eq 8

It’s the small possible program. Let *C* be another computer. If this complexity is general there is a universal computer *U* that simulates *C* for any string *x* by a constant *c* on computer C. Thus

$$K_{c}{\left(x\right)}_{U\;\leq }K_{c}{\left(x\right)}_{C}+\;c$$ Eq 9

#### Measuring the randomness of a string

Let *K*_*c*_ *(x|y)*: The conditional Kolmogorov complexity of *X*_*n*_ given *Y*. Consider for example we want to find the binary string with higher complexity between three variables *X*_*1*_(010101010101010), *X*_*2*_(0111011000101110) and *Y* (01110110001011). In Table 5 we have the representation and minimal encoding using *X*_*n*_ and *Y*. Therefore, we can see *X*_*1*_ and *X*_*2*_ can be encoded as combinations of *Y* and thus *K*_*c*_*(X*_*1*_*|Y) > Kc (X*_*2*_*| Y)*. This relationship is defined as

$$K_{c}\left(X,\;Y\right)=K_{c}\left(X|Y\right)+K_{c}\left(Y\right)=K_{c}\left(Y|X\right)+K_{c}\left(X\right)$$ Eq 10

**Table 5:** Example of representation of strings X1 and X2 using substring Y.

Variable	Binary Sequence	Minimal String Representation Schema
Y	01110110001011	Substring
X_1_	010101010101010	01 (#7 pairs of bits) + 0 (#1 single bit)
X_2_	011101100010110	Y + 0

In Fig 18 from [24] we can see a comparison between a series of strings and the correspondent automata state machine and the regular expression patterns (i.e. regex schema).

**Fig 18:**
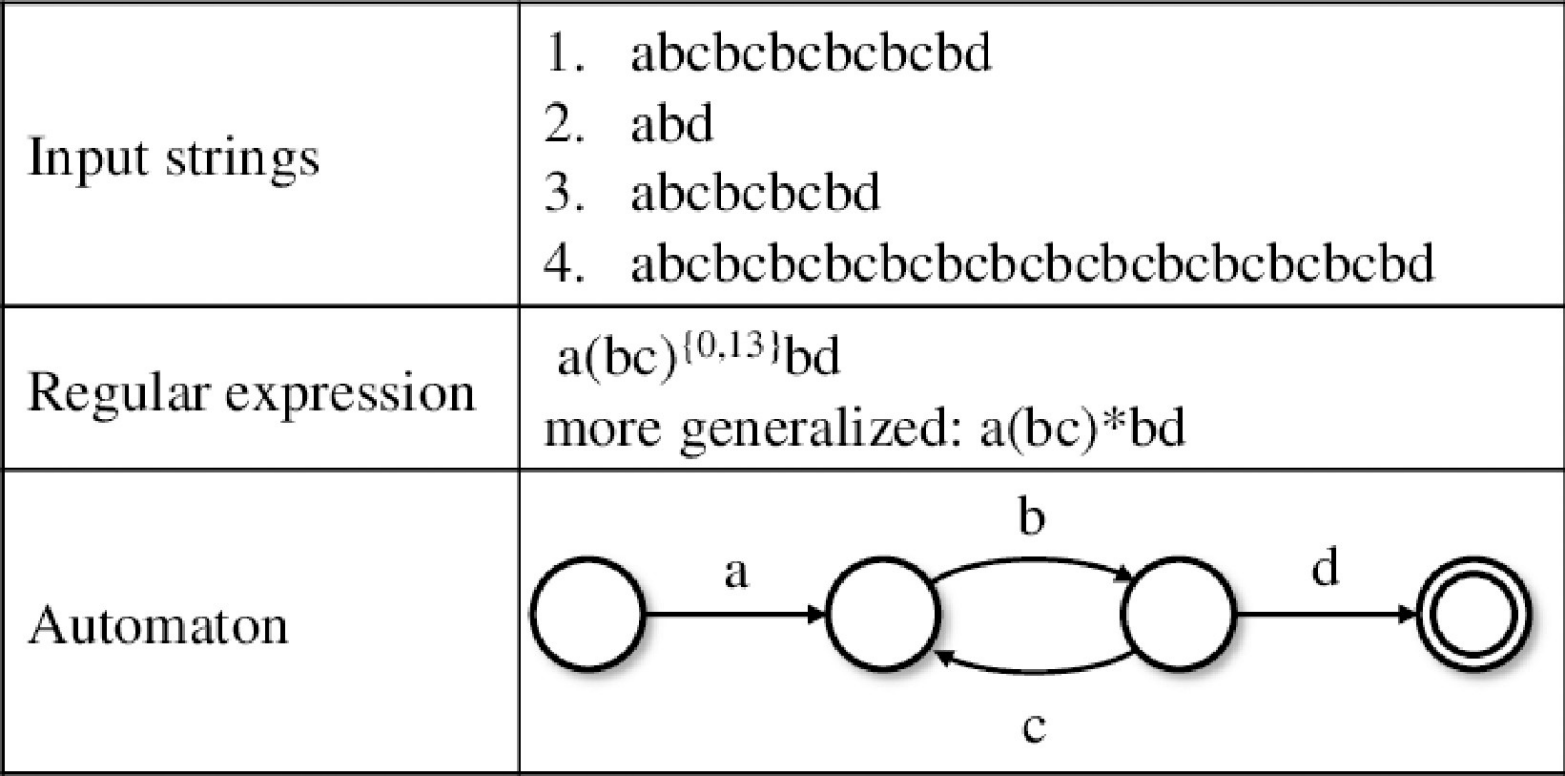
Examples of representations of a given input string set using regex and an automaton.

The expected value of the Kolmogorov complexity of a random sequence is close to the Shannon entropy. From [25] this relationship between complexity and entropy can be described as a stochastic process drawn to a i.i.d on variable *X* following a probability mass function *pdf(x)*. The symbol *x* in variable *X* is defined by a finite alphabet *L*. This expectation is

$$H\left(X\right):\;E\left(1/n\right)\;K_{c}(X^{n}n)$$ Eq 11

#### Kelly criterion and the uncertainty in random outcomes

The Kelly strategy is a function for optimal size of an allocation in a channel. It calculates the percentage of a resource that should be allocated for a given random process. It was created by John Kelly [26] to measure signal noise in a network. The bit can be interpreted as the amount of entropy in an expected event with two possible binary state outcome and even odds. This model maximizes the expectation of the logarithm of total resource value rather than the expected improvement for the utility function from each trial, in each clock unit iteration in a Turing Machine.

The Kelly criterion has applications in gambling and investments in the securities market [27]. In those special cases, the resource (communication) channel is the gambler’s financial capital wealth and the fraction is the optimal bet size. The gambler wants to reduce the risk of ruin and maximize the growth rate of his capital. This value is found by maximizing the expected value of logarithm of wealth which is equal to maximize the expected geometric growth rate.

Similarly, the log-normal Salesman’s can improve his strategy in the long run by quantifying the total of available inside information in the channel (or a tape in the Turing machine) and maximizing the expected value of the logarithm of the value function (defined by Traveled Euclidean distance) for each execution clock. Using this approach, he can reduce his uncertainty (entropy H(X)) while optimizing his rate of distance reduction (and solution quality improvement) at each execution time.

**Fig 19:**
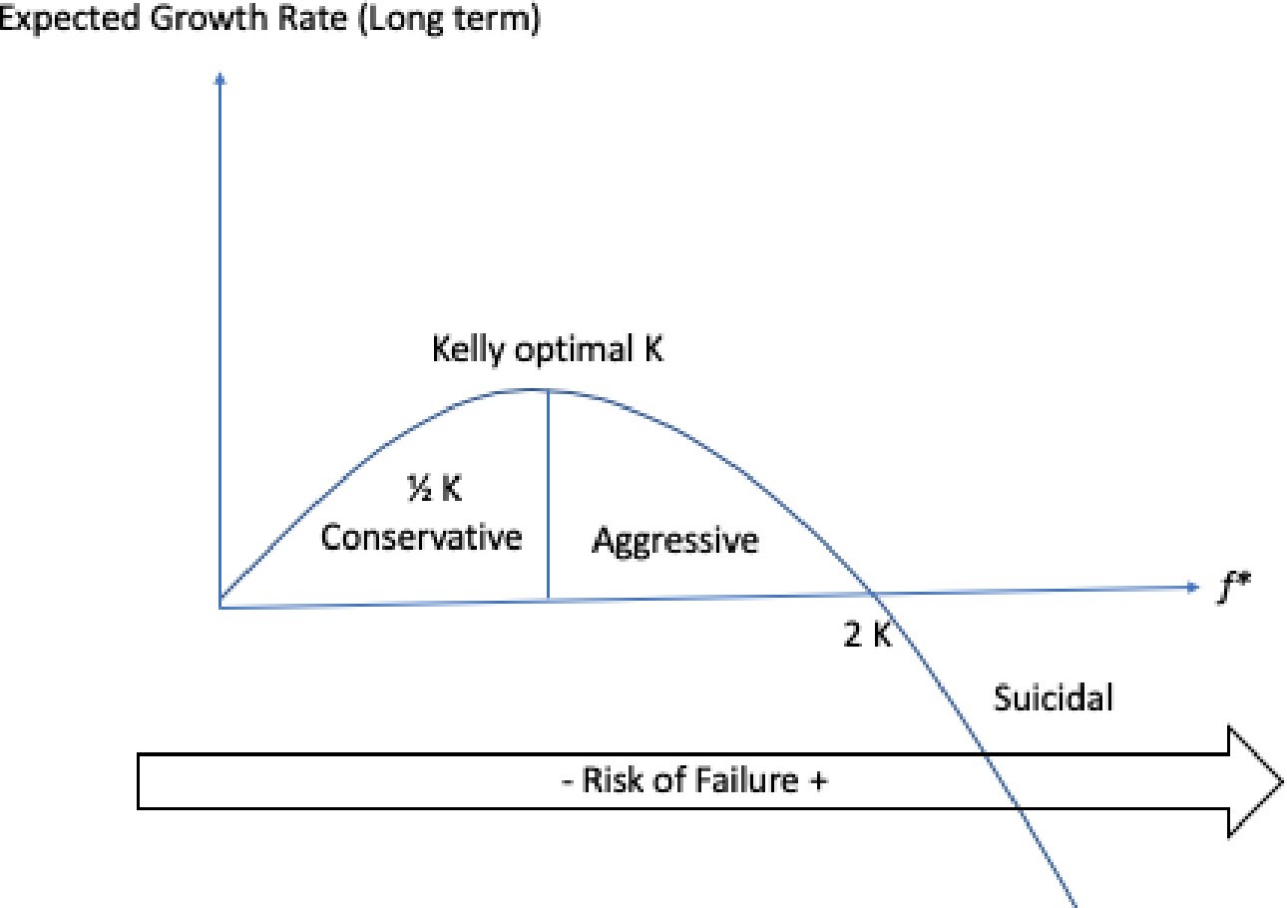
Maximization of entropy in random events.

The Fig 19 demonstrate the Kelly criterion value over the Expected Growth Rate.

#### Kelly uncertainty distribution

Let *E(Y)* be the expected value of random variable *Y*, *H(Y)* be the measured entropy for the *pdf(Y)* distribution and K*(E(Y),H(Y)) = f* be the maximization of the expected value of the logarithm of the entropy of the utility function *Y = g(X)*. This fraction is known as the Kelly criterion and can be understood as the level of uncertainty about a given data distribution of the random variable *X* relative to a probability density function *pdf(Y)* of a measured respective cost distribution found at the sample. It’s a measurement of the amount of useful encoded information.

The value of f* is a fraction of the cost-value of *g(X)* on an outcome that occurs with probability p and odds *b*. Let the probability of finding a value which improves *g(X)* be p and in this case the resulting improvement is equal to 1 cost-unit plus the fraction *f*, (1+*f*). The probability of decreasing quality for *Y* is *(1-p)*.

The maximization of the expected value f* is defined by the Kelly criterion formula in Eq 12

f*=((pb+p–1)/b) Eq 12

Where f* is the optimal fraction, b is the net odds, p is the probability of improving quality (success-win) in Y = *g(X)* and the q is the probability of decreasing (failure-loss) quality q = (1-p).

For example, consider a program with a 60% chance of improving the utility function g(X) thus p = 0.6 (success) and q = 0.4 (failure). Consider the program has a 1-to-1 odds of finding a sequence which improves g(X) and thus b = 1(1 quality-unit increase divided by 1 quality-unit decrease). For these parameters the program has a 20%(f* = 0.20) of certainty that the outcomes produce values that improve the expected value of g(X) over many trials.

Consider another sample case for a fair coin with probability of success (winning) *P (1) = 0*.*5(50%)* and failure(losing) *P (0) = 0*.*5(50%)*. Table 6 demonstrate the amount improved (+) and worsen (-) for each scenario:

**Table 6:** Yield returns from bernoulli process P(1) and P(0).

Probability	Output	Value
Success (1)	Gain (True)	+ 2 units
Failure (0)	Lose (False)	- 1 unit

The Total Expected Outcome, *Edge*, is defined by:

Edge=(50%*2units)+(50%*1unit)=0.5units Eq 13

The Kelly criterion can be calculated alternatively as:

f*=Edge/Odds

LetOdds=AmountReturnedifSuccess

f*=0.5units/2units=0.25=25% Eq 14

Therefore, there are 25% bits of useful information in the noisy channel.

## Proposed algorithm and results

### Quantitative algorithmic information Theory

The algorithm is designed to find the near optimal route in a cluster of machine-nodes before returning to the original point. This problem is a variation of TSP. Tour improvement heuristics algorithms such as 2opt and Simulated Annealing (SA) are used as a benchmark for the proposed Quantitative Algorithm (QA). 3 test cases are used to analyses the solutions generated by each algorithm. The 2opt algorithms produces solutions with smaller total distance but required more time units as the number of nodes increase. SA and QA have a maximum number of allowed interactions, but QA produces better solution quality than SA for the same time period.

The test samples are grouped in 10, 30 and 50 nodes. Each point represents a machine in a data center (i.e. computing and network provider) that can deploy a given service S. The distance cost in this case is the illustrative round-trip network and processing delay. This weight is the length of time to send a signal s*(t_*0*_*)* plus the time to reply acknowledging of that the signal was received with another message s*_ack_ *(t*_*1*_*)*.

To avoid bias and miss interpretation in the research, the first tour loaded in the computer memory is randomly flushed using a statistical function in Python programming language. The function swaps all elements (using a normal distribution) of the initial tour list, created after reading the list of input nodes.

The solutions found from SA and QA heuristics algorithms were analyzed for accuracy and reliability of the output. We have compared the required time and solution quality between the methods. Each algorithm was measured with a trial with sample length N = 60 for 3 test cases.

### Quantitative heuristic

**Fig 20:**
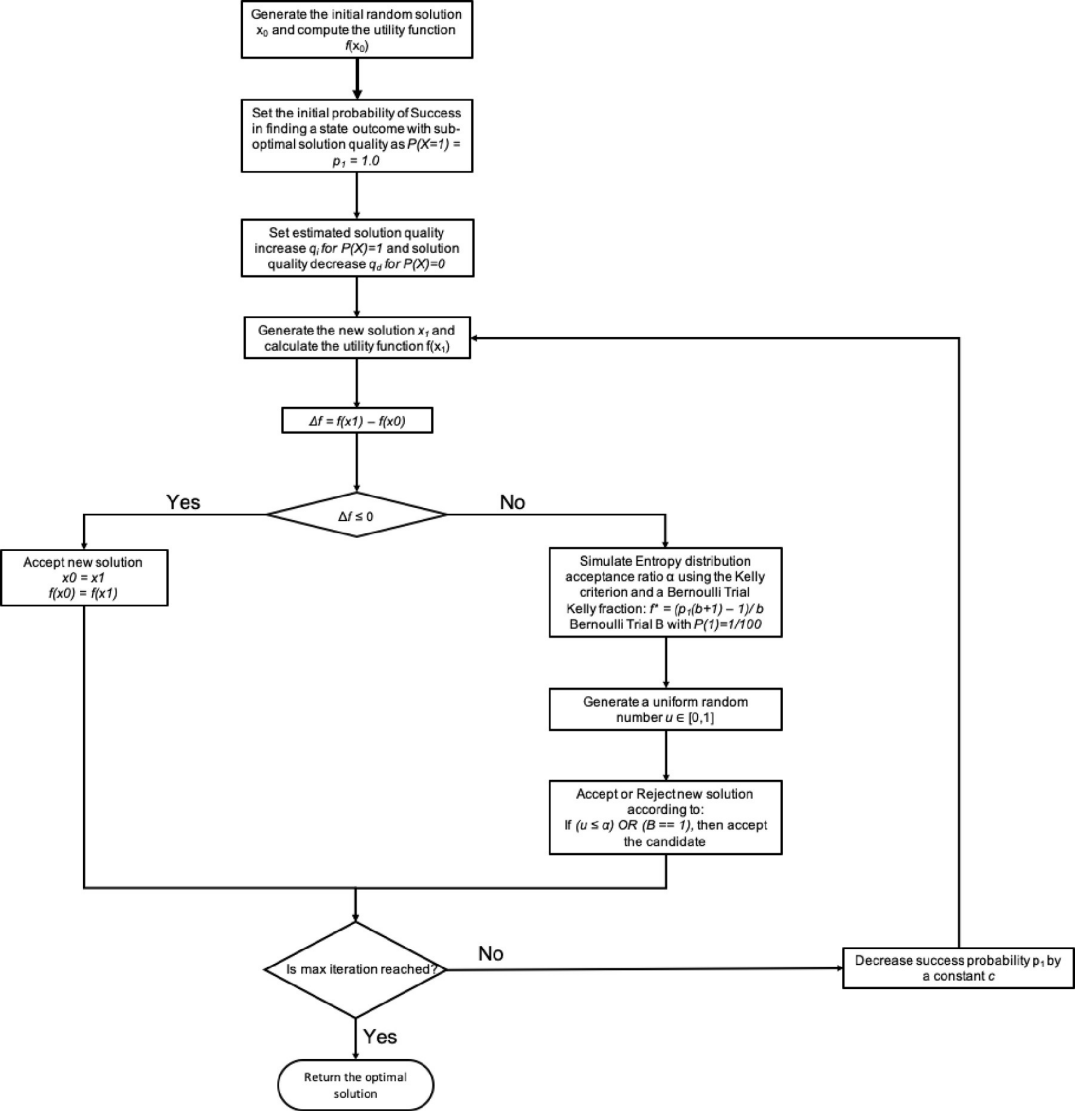
Flowchart for the Quantitative TSP algorithm based in information theory.

**Fig 21:**
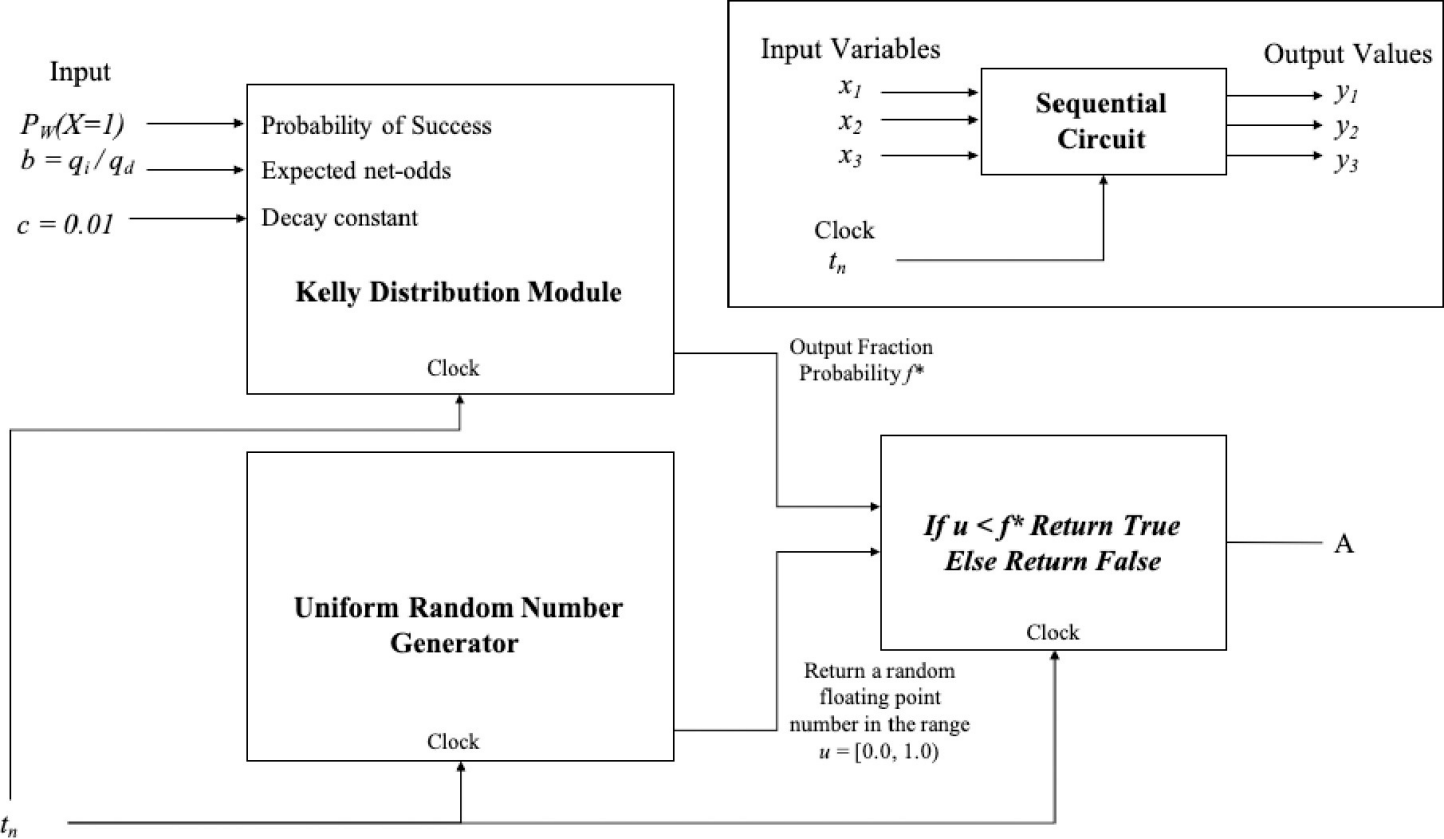
Decision Criterion 1 is implemented as an evaluation of the IF logical statement for the Simulated Kelly Criterion and the Uniform Random Number output value.

In Fig 20 we have the flowchart design for the Quantitative TSP Algorithm (QA). The constraints for the Kelly criterion and the Bernoulli process are presented in Figs 21 and 23. The pseudo-code for the proposed Quantitative Algorithm (QA) is describe in the Table 7.

**Table 7:** Proposed pseudo-code algorithm to solve the TSP.

Quantitative Algorithm for TSP 1. Generate the initial random solution x_0_ and compute the utility function *f*(x_0_) 2. Set the initial probability of Success in finding a state outcome with sub-optimal solution quality as 1. *P(X = 1) = p*_*1*_ *= 1*.*0* 2. and the Failure probability defined as 1. *P(X = 0) = q = p*_*0*_ *= 1—p*_*1*_ 3. Set estimated solution quality increase *q*_*i*_ *for P(X) = 1* 4. Set estimated solution quality decrease *q*_*d*_ *for P(X) = 0* 5. Set the net fractional odds ratio as *b = q*_*i*_ / q_*d*_ 6. For *i* in range (0, M) 1. Generate the new solution x_1_ and calculate the utility function *f*(x_1_) 2. Δ*f = f*(x_1_)–*f*(x_0_) 3. If Δ*f ≤* 0 then 1. Accept new solution 2. x_0_ = x_1_ 3. *f(x*_*0*_*) = f(x*_*1*_*)* 4. Else 1. Simulate Entropy distribution acceptance ratio α using the Kelly criterion and a Bernoulli Trial 1. Kelly fraction α: f* = (p_*1*_*(b+1)– 1)/ b* 2. Bernoulli Trial *B* with *P(1) = 1/100* to work as a random binary switch 2. Generate a uniform random number *u* ∈ [0,1] 3. Accept or Reject new solution according to: 1. If (*u ≤ α) OR (B = = 1)*, then accept the candidate 1. Set x_0_ = x_1_ 2. *f(x*_*0*_*) = f(x*_*1*_*)* 2. Else then reject the candidate solution 3. Decrease success probability *p*_*1*_ by a constant *c* 1. *p*_*1 =*_ *p*_*1*_*–c*

### Algorithmic information model

The two major components are the simulated Kelly fraction f* (describing the overall uncertainty spread) and the Bernoulli process distribution of the underlining random event between states (estimated as the mean and standard variance for the weight function for each solution). The combination of those factors will be evaluated to decide the start of a neighborhood search (following a Probability density function) when the new alternative solution has a negative gain (i.e. new proposed solution is worse than current best-known encoded candidate).

Solutions to the TSP routing problem are explored by algorithms such as 2opt, Simulated Annealing (SA), Greedy and Genetic Algorithm (GA). In this paper we proposed a Quantitative Algorithm (QA) that does not rely on naturally inspired schemas but rather provides a statistical interpretation as a distribution of signals by a stochastic log normal process.

This stochastic process is defined as an ordered list of random variables *{X*_*n*_*}* for a given trial of length *N*. *N* is a set of non-negative integers and *X*_*n*_ is defined as a target measurement for a specific instance of time.

The utility function is used to find the near optimal route that have the minimum traveling distance to multiple target node destination while returning to the starting node at the end. There are 2 constraints to be considered in the model presented in this paper: Simulated Entropy Uncertainty and Bernoulli Process.

#### Method Constraint 1: Entropy simulation and uncertainty measurement

**Simulated Uncertainty.** The first constraint is limited by the entropy. The input parameters for the Kelly function f* are the Wining probability *P*_*W*_ and the expected net-odds *b* for the Bernoulli trial *B*. The value of *P*_*W*_ is decreased by a fixed constant rate of 0.0001 at each interaction. The value *b* is measured as the ratio of average improvements of the positive interactions divided by the average reduction of the negative interactions. The result of this function is the percentage of the useful side information available in a noisy channel.

In Table 8 we have an example for the Kelly criterion calculation.

**Table 8:** Example calculation for the Kelly criterion for P (1) = 90, 50 and 10 and net-odds b = 1.

P_W_ (Win/Cost Decrease)	P_L_ (Lose/Cost Increase)	Expected (Averaged) Cost Gain (Quality Improvement)	Expected (Averaged) Cost Loss (Quality Worsening)	Success/Failure Ratio b	(Output) Kelly Percentage f*
90	10	+100	-100	+100/|-100| = 1	0.8
50	50	+100	-100	+100/|-100| = 1	0
10	90	+100	-100	+100/|-100| = 1	-0.8

The decision criterion 1 is implemented as an evaluation of the IF logical statement for the Simulated Kelly Criterion and the Uniform Random Number output value.

### Logical Statement for Method Constraint 1

For each interaction in the program

$$\begin{array}{l} \begin{array}{l} \text{Produce}\;\text{uniform}\;\text{random}\;\text{number}\;u\in \left(0,1\right]\\ \text{Calculate}\;\text{the}\;\text{Kelly}\;\text{fraction}\;f*=\left(P_{W}\left(b+1\right)–\;1\right)/\;b\\ \text{Ratio}\;\text{of}\;\text{solution}\;\text{quality}\;\text{improvement}\;b=Success\;/\;Failure\\ \text{Conditional}\;\text{Logical}\;\text{Statement}\;A:=u\;\leq \;f* \end{array}\\ \text{Decrease}\;P_{W}\;\text{by}\;\text{a}\;\text{constant}\;c \end{array}$$ Eq 15

In Fig 21 we have a circuit representation of the first constraint and the representation for a sequential circuit with input and output values with a clock.

#### Method Constraint 2: Discrete computation distribution of a random binary variable

**Bernoulli Process.** The second constraint is defined by a Bernoulli process as a finite sequence of independent and random Bernoulli variables. This module will return as output the value “True” at 1% of the time for 100 interactions (N = 100) and it will accept unlikely (risky) solutions with negative gain to eventually provide improvements bets for the solution quality, under some degree of freedom. The process is defined as a trial with two binary states either “True/Success” (1) or “False/Failure” (0) with domain p ∈ [0,1]

$$\begin{array}{l} \begin{array}{l} P\;\left(X_{i}=1\right)=p\\ P\;\left(X_{i}=0\right)=1–p\\ E\left(X\right)=p \end{array}\\ Variance\;Var\;\left(X\right)=p–p^{2} \end{array}$$ Eq 16

**Fig 22:**
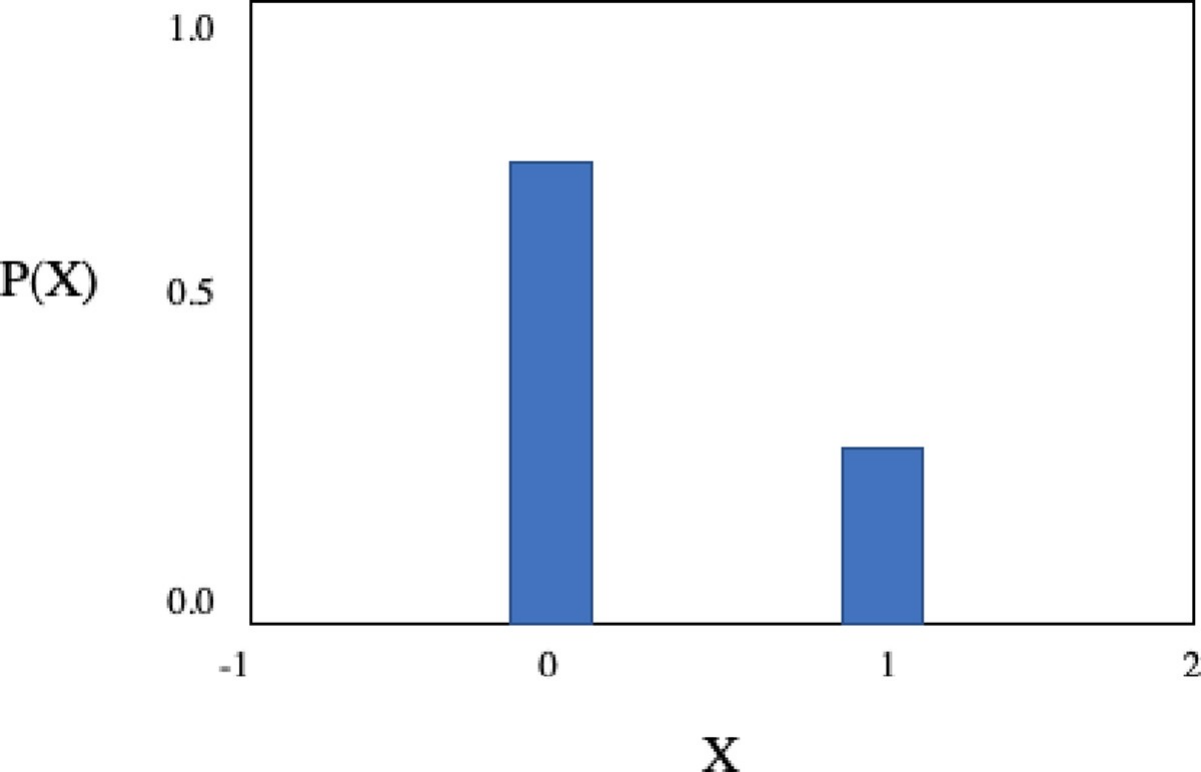
P(X) for the Bernoulli distribution with P(1) = 0.2 and P(0) = 1—P(1) = 0.8.

In Fig 22 we can see a Bernoulli distribution with P (0) = 80% probability of output a Failure state

$$\begin{array}{l} \begin{array}{l} P\left(X=0\right)=0.8\\ P\left(X=1\right)=0.2 \end{array}\\ Otherwise\;P\left(X\right)=0 \end{array}$$ Eq 17

Examples of Bernoulli trials are show in Table 9.

**Table 9:** Examples of random events and the respective binary outcome.

Event	Outcome
Play a game	Win/Lose
Coin toss	Head/Tail
Processing a request	On time/Late
Defect in equipment’s	Good/Defect
Buy-Sell an asset	Profit/Deficit
Optimizing traveling cost	Reduction/Increase

The decision criterion 2 is implemented as a OR gate using the input Bernoulli Distribution Trial B and the output A of the IF statement from the Simulated Kelly Criterion and the Uniform Random Number output value.

### Logical Statement for Method Constraint 2

For each interaction in the program

$$\begin{array}{l} Bernoulli\;Trial\;B\;with\\ P_{1}\left(X\right)=P_{1}\left(1/100\right)=True=Success=1\\ P_{0}\left(X\right)=1–P_{1}=False=Failure=0\\ \text{Conditional}\;\text{Logical}\;\text{Statement}\;B\Leftrightarrow 1\\ \text{Conditional}\;\text{Logical}\;\text{Statement}\;A⊕B \end{array}$$ Eq 18

**Fig 23:**
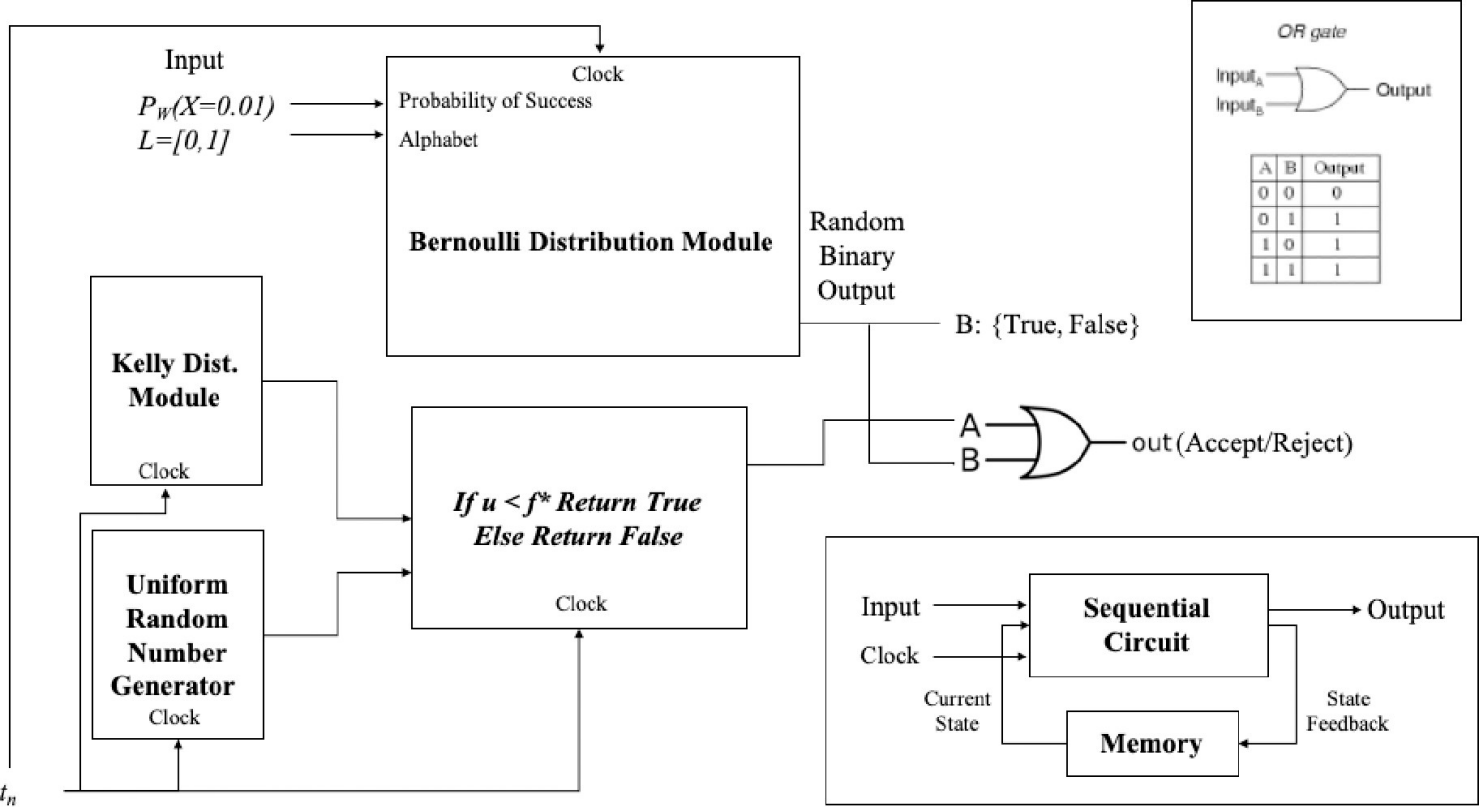
Decision Criterion 2 is implemented as a OR gate using the input bernoulli distribution Trial B and The output A of the IF statement from the Simulated Kelly Criterion and the uniform random number output value.

In Fig 23 we have the circuit representation of the second constraint and the memory feedback loop for the program.

### Algorithm analysis

This paper tries to address a few algorithm`s design questions to solve the TSP:

1. What is the shortest code that produces the optimal solution for the problem?
  - Objective: What is the minimal binary encoding, the self-information or the basic quantity required to represent the Bernoulli process
2. What is the path with less risk “of ruin” for the Salesman while also maximizing the solution quality?
  - Objective: Find the shortest Euclidian distance with less uncertainty
3. How to minimize running time requirements?
  - Objective: Optimize computational complexity

**Fig 24:**
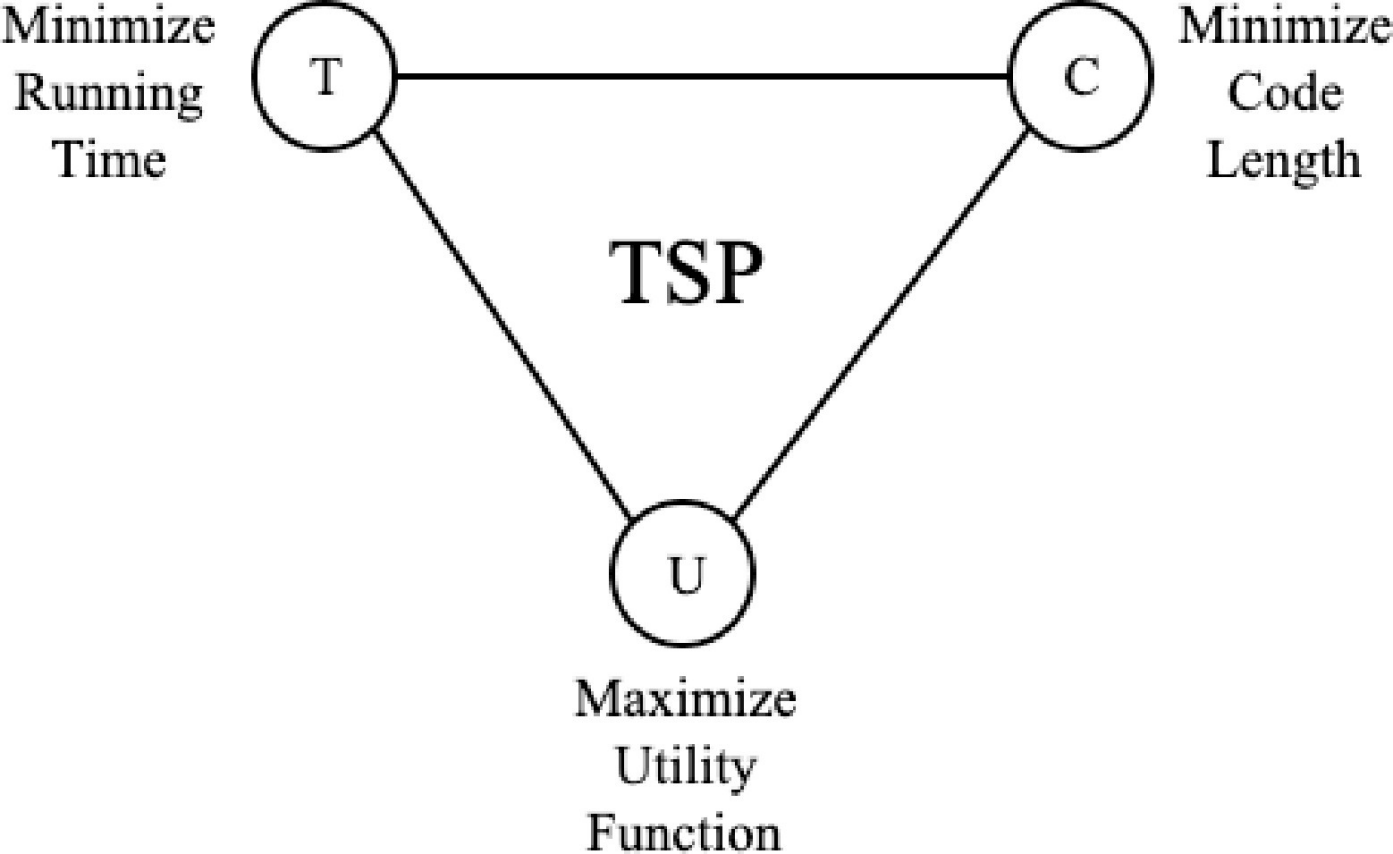
TSP target objectives.

Fig 24 illustrate the TSP goal to optimize solution quality and the factors involved: Minimize Running Time; Maximize Utility Function and Minimize Code Length

#### Shortest distance and shortest encoding

The computational complexity problem in Graph Theory such as the TSP and NP-Hard Problems is reduced to a combinatorial problem, using an entropy function optimization method from a Bernoulli variable defined by a Log-Normal distribution.

The method is based on the amount of self-information available in a random variable and this is the minimum encoding required to represent the problem in a Turing Machine, while producing as output the near-optimal solution found before halting. By the Kolmogorov Complexity this is the optimal program with the minimum amount of encoded information.

#### Mathematical definition

Assuming the Euclidian distance between nodes is the utility function for the problem. The proposed algorithm instead of trying to find the best solution directedly, using an arbitrary stochastic process, and interactively improving the string path, alternatively, the QA algorithm assumes the symbols from output Y is produced by processing the input from Bernoulli variable X, that is defined by a log-normal distribution and variables X and Y share Mutual Information. This is the amount of information X has about Y and is equal to the information Y know about X.

The lemmas for the proposed model are defined in Table 10.

**Table 10:** Auxiliary theorems for the proposed model based in information theory.

1. The Bernoulli process *B(p)* is used to generate strings with length *n* and a pattern-code schema encoded as a Hamilton graph with probability *p* 2 The strings *S* are a Bernoulli sequence *B* formed with a random variable *X* from a finite alphabet L 3. The utility function U*(S=B(X)) = Y* is defined as the Euclidian Distance between nodes in a 2D plane 4. Bernoulli variables *X* and *Y* have Mutual Information 5. The Entropy Function for *H(X)*, *H(Y)* and *H(X|Y)* can be calculated 6. *E(X)* is the Expected Value for *X* with mean ${\overset{¯}{x}}_{1}$ and standard deviation *σ*_*1*_ 7. *E(Y)* is the Expected Value for Y with mean ${\overset{¯}{x}}_{2}$ and standard deviation *σ*_*2*_ 8. f*(Y) is the optimal encoding defined by the Total Expected Value (“Edge”) divided by the Expected Solution Quality Increase if Successful (“Odds”), for Sequence Y *a*. f*(Y) = Edge/Odds

#### Logarithm utility model

**Fig 25:**
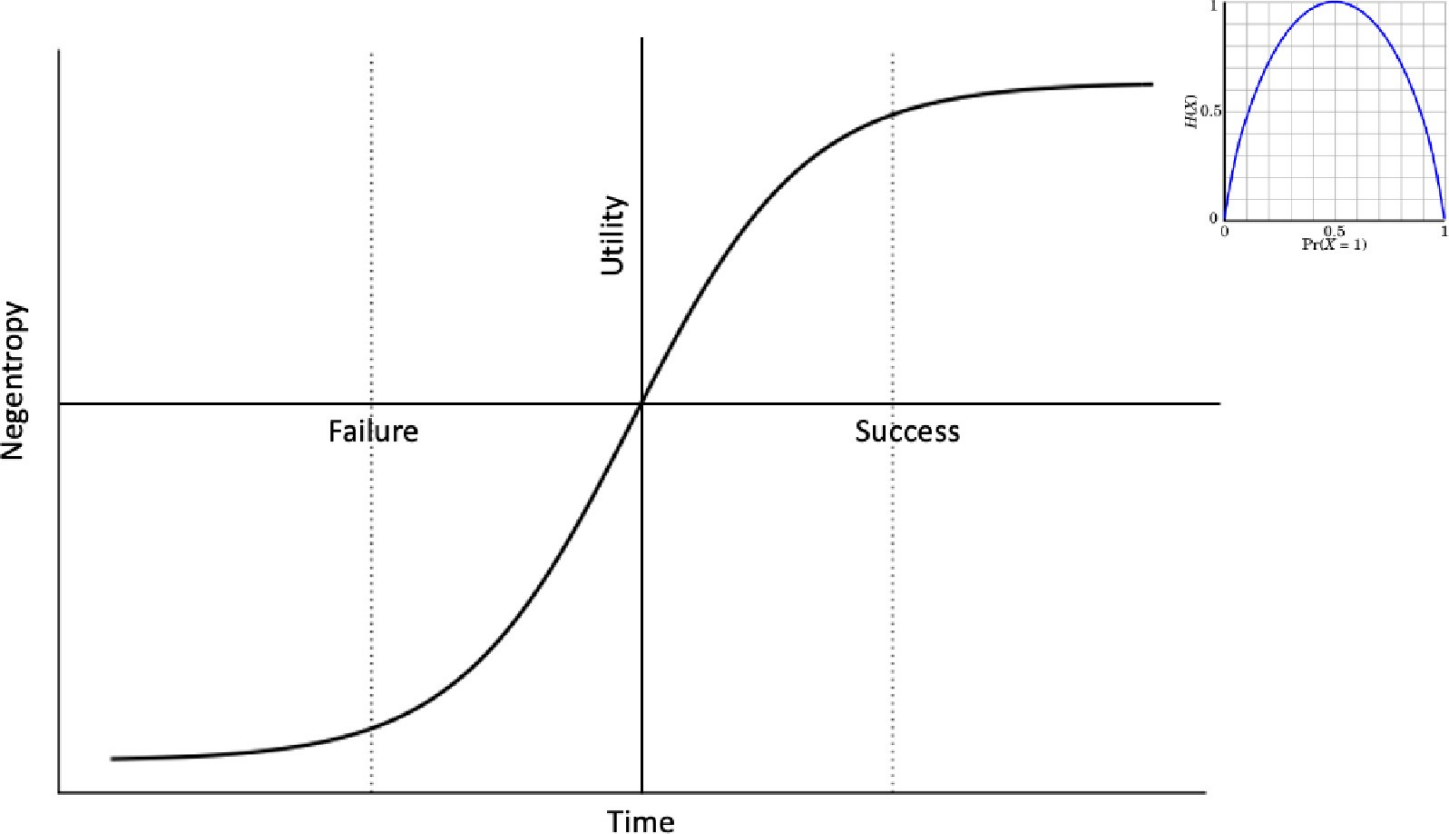
Utility function.

The method provides an alternative narrative for the Traveling Salesman Problem as it asks, *“what is the best path between cities that simultaneously minimizes cost losses in the long run while also provide the best rate of improvement for each interaction”*. The salesman wants to avoid solutions with local minima. There are several possible paths the salesman can choose, and he must guess which choice will lead to the best route over a set of candidate solutions. The uncertainty between states outcomes indicates the level of entropy (i.e. more entropy means less knowledge about the state values distribution). The reverse of entropy is the negentropy, which is defined as a temporary state condition in which a certain state distribution is more probable and more organized and thus there is less uncertainty about the state distribution for a given time frame. The utility function is demonstrated in Fig 25.

This approach is a model for risk measurement over many possible outcomes and is based in the work of John Kelly, Edward O. Thorp, von Neumann and Claude Shannon in logarithmic information utility, with applications in the financial markets and game theory such as the optimization in wealth growth in the long run and diversification in investment strategies in portfolio management.

#### Random path construction

**Fig 26:**
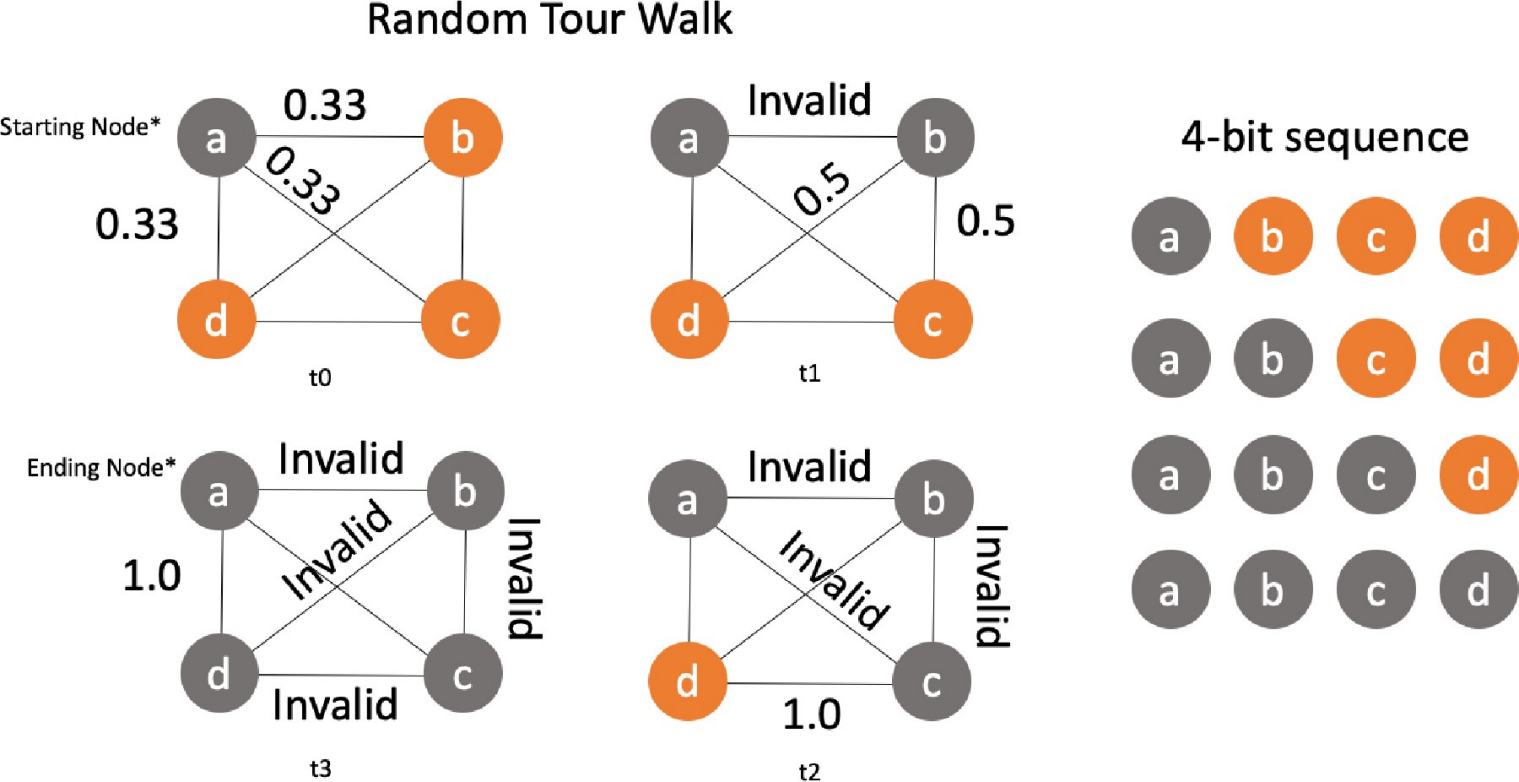
Tour construction for a weighted graph with a probability function p ∈ [0,1].

In Fig 26 we have a representation of the possible path choices to produce a candidate solution tour for TSP. The example has an alphabet *L* with 4 elements (*n = 4*). At each interaction the Salesman’s needs to choose the next node in the route. In the begging at *T = t0*, starting in the arbitrary node *L*: *{a}*, it has several possibilities for the next move with *L’*: *{b*,*c*,*d}*. Each element (or symbol) has a 1/3 probability in t0. In the second iteration t1, the probability for the remaining possible symbol increase to 0.5, however the probability for the nodes already chosen drops to zero, and the possible choice outcome for the next state move is reduced to *L’*: *{c*,*d}*.

From initial solution in time *T = t0* there are a few valid sequences that can be chosen. There is no universal function to guide the Salesman with certainty on what is the best choice. This statement is supported by the Kolmogorov complexity of the string. The Salesman must then outguess this problem using the available side-information.

**Fig 27:**
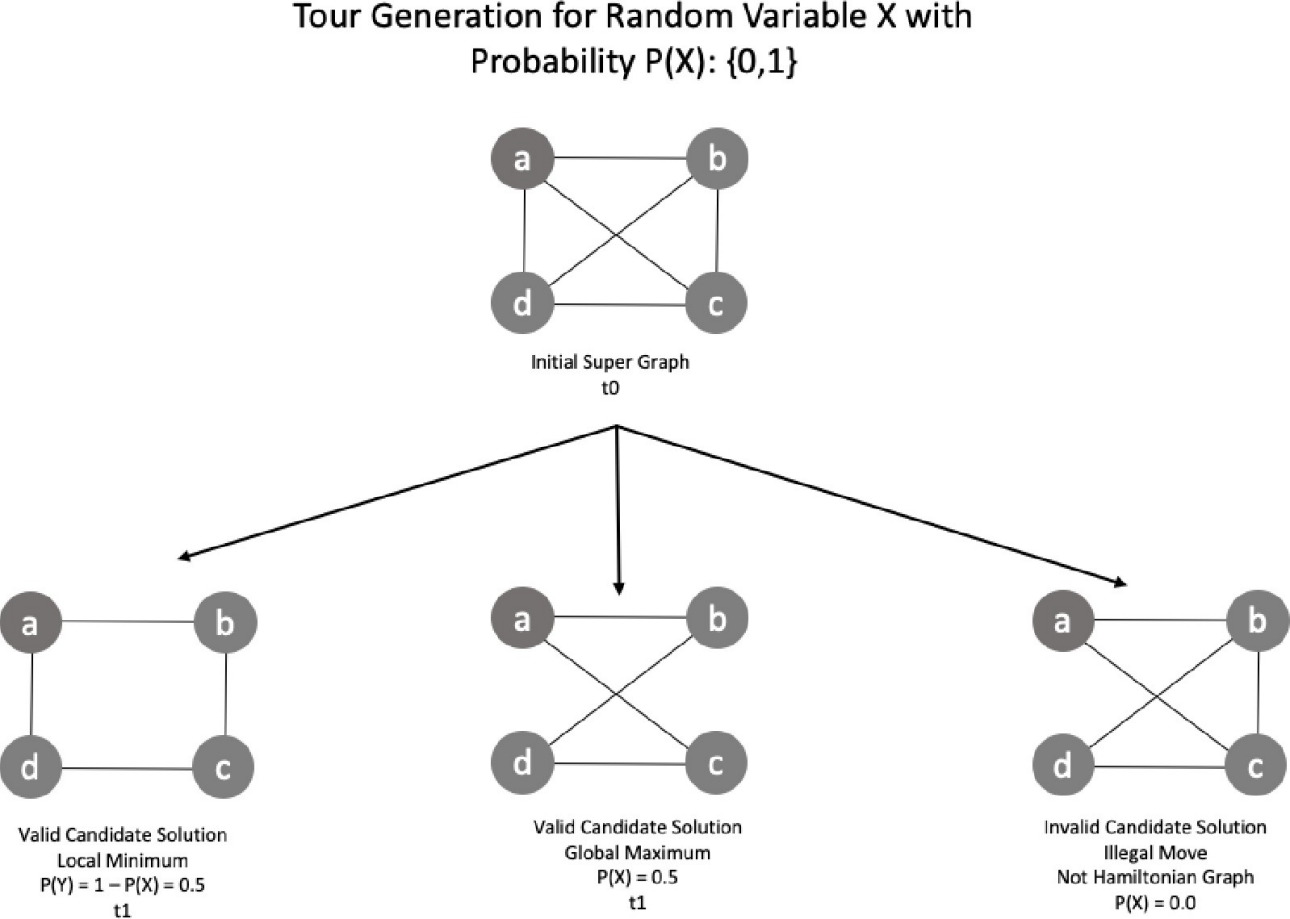
Candidate solution production and path decision.

The side-information can be defined by the probability density function of the Euclidian distance distribution, formed by a set of valid candidate solution strings. The condition for a candidate to be valid is to be a Hamiltonian graph. Invalid solutions have zero probability of being chosen. In Fig 27 we have a set of candidate solutions with examples of valid and invalid sequences.

**Fig 28:**
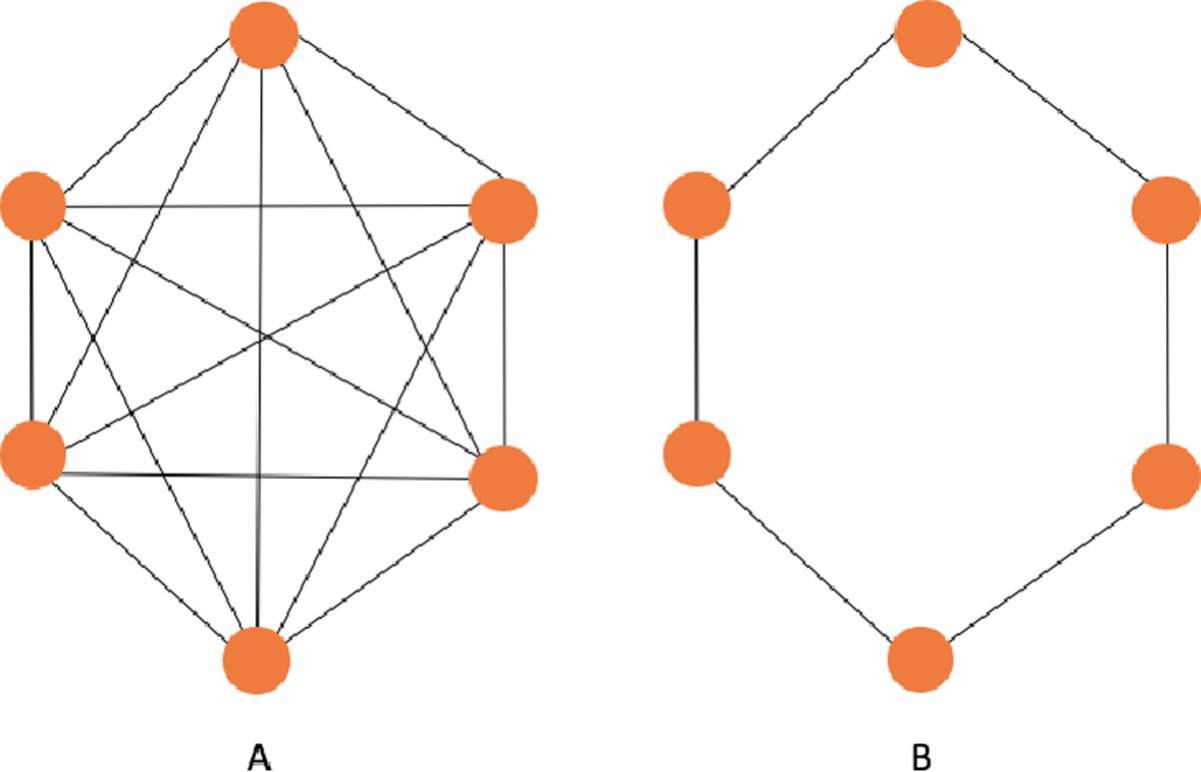
Edge crossing between nodes.

This feature reduces the search space to only Hamiltonian circuits. From observation, we know that there is a greater probability that edges that crosses generally produce a total path distance larger than those nodes that do not have edges that overlap. The Fig 28 demonstrate a crossing of edges in a graph.

Algorithms such as 2opt takes advantage of this cluster aggrupation of edges and uses a strategy to remove the crossing between a group of nodes until no further improvement is found.

#### Solution quality and time to convergence

The method proposed is not an exact method but is guaranteed to produce the best quality in the long run, eventually, with probability 1 and smaller time to convergence, in average. This is a method that almost surely converges to a better solution quality if compared to any other heuristics when approaching the limit, as the number of algorithm iteration goes to infinity, and without needing complex string and array manipulations. Therefore, the method has a better computational cost optimization in average.

The proposed algorithm also brings more simplicity of implementation with lower encoding overhead, when compared to other algorithms such as Genetic Algorithm, Neural Networks and Ant Colony.

#### Computational requirements and cost effectiveness

The time constraining in QA heuristic is not a problem because the only factor to decide the running time is the degree of freedom allowed to the program. If the algorithm is expected to be more precise and with less errors, the time parameters can be adjusted with time complexity *O(n)* and with each interaction the entropy about the solution states is reduced, as the knowledge about the best solution increases (i.e. Smaller Euclidian distance). Therefore, the error rate can be adjusted to any level desired.

#### Relation with other heuristic methods

This is similar to the approach proposed by the Cross-Entropy Method, but the Quantitative Algorithm method does not resample or update the probability density function at each interaction, but instead embraces randomness and accepts candidate solution according to a simulated decreasing entropy function, with a binary random switch attached.

This mechanism is similar to the Manhattan Rule used by the Simulated Annealing algorithm. The proposed method use the simulated Kelly function ratio value α = f* to compare against a random control value *u*, than the output is evaluated again using a binary operation switch *B* which returns True or False, following a dependable probability function, that is decremented by a constant rate *c* from P(X) = 100 until P(X) = 1/100, at each interaction *t*_*N*_. This schema is used to provide alternatives paths and solve the hill-climbing limitation that is also present in many solvers of the TSP.

**Fig 29:**
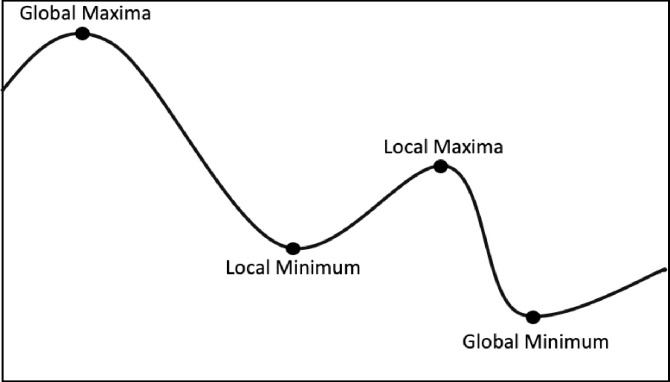
Hill-Climbing problem.

In Fig 29 we have a demonstration for the hill-climbing with examples of Global and Local points with the respective Maximum and Minimum positions.

#### Running costs

The running cost for the Quantitative Algorithm is the defined by the computational effort to generate a random candidate tour string and to evaluated with the current registered best-known solution, encoded by a Hamiltonian graph pattern, in *O(N)* time.

For each iteration, it has the computing cost to calculate the simulated entropy heuristic function and compare against the control values. These values can be calculated in constant time *T(n)*. The memory required is defined by an array M of candidate solutions elements with size *O(M) = 2*. The array is a buffer to store the best candidate solution and the alternative solution.

#### Disadvantages and Limitations

Because of this design, the running time to converge is worst for the QA algorithm when compared to other methods, such as 2opt and greedy, at small instances of the problem with less than a few nodes (n<15). If compared to heuristics such as Simulated Annealing, Genetic Algorithm or Ant Colony the execution time is similar but requires less time in average. This behavior is observed in our computer simulation.

This is explained by the overhead in calculating the probabilistic temperature-function in Simulated Annealing (or the mutate-reproduce-selection operations in Genetic Algorithms) that are used as the acceptance criteria for the random candidate solutions and therefore the proposed entropy derived function in QA has a smaller running time overhead.

The running time cost for the proposed algorithm scales linearly to input with length *n* and the solution can be found in polynomial time in any Turing Machine. In Table 11 we have a comparison between classes of computational time complexity.

**Table 11:** Classes of computational complexity.

*Runtime Complexity*	Computational Class	Time to Convergence (1/8)	Solution (Search)Space Volume	Example Algorithm
*O(1)*	Constant	0000000000	0000000000	
*O(log n)*	Logarithmic	0000000001	0000000001	Binary Search
*O(n)*	Linear	0000000011	0000000011	Linear Search
*O(n log n)*	Linearithmic time	0000000111	0000000111	k-opt method
*O(n*^*2*^*)*	Quadratic	0000001111	0000001111	Nearest neighbor (NN)
*O(n*^*3*^*)*	Cubic	0000011111	0000011111	Christofides and Serdyukov
*O(n*^*k*^*)*	Polynomial	0000111111	0000111111	A*
*O(2*^*n*^*)*	Exponential	0001111111	0001111111	
*O(n*!*)*	Factorial	0011111111	0011111111	Brute-Force
*O(inf)*	Infinite Time	0111111111	0111111111	

The proposed method has limitations. It needs a larger running time to converge for small instances if compared to heuristics such as 2opt and Greedy Algorithm, as it must run until the predefined max number of interactions parameter value is reached.

We have also observed that the local search methods such as 2opt, converge with less running time when the input is pre-sorted and thus help avoiding the algorithm to being lock down in local minima solutions. However, this implies that a predefined prefix schema must be directly encoded in the input string values, with the goal to improve running time performance. The pre-processing of the input string works as a prefix set for filtering “above average” solutions. This is explained by the Kolmogorov randomness of a string.

Alternatively, the proposed method does not need such implementation bias. This behavior can be explained by Algorithm Information Theory. The 2opt method for example is sensitive to the initial solution set sequence and if provided with an initial state with more noise than the running time also increases.

This means there are more elements to verify in the search space and thus more time is needed until converge. In the other hand, if there is less noise in the initial state, than the sub-optimal end state is found quickly. In comparison the proposed model is resistant to variations in the initial path sequence positions.

## Computational results

The research evaluates the performance of the proposed algorithm through a series of test cases and statistical analysis. The new method is tested against the traditional method Simulated Annealing (Benchmark). Each test case was run for a trial with population length of N = 60. In the statistical analysis section, the t-test was used to compare the means between the sample groups. The null hypothesis is that there is no difference between the means of the two populations.

The input parameters where tested with different values in order to verify if the method was sensitive to changes in the controlling parameters. In the numerical simulation we have implemented 3 variations for graphs with 20, 30 and 50 nodes. Each 2D graph was used as input for the Quantitative Algorithm (QA) and the benchmark heuristic algorithm Simulated Annealing (SA). The maximum number of interactions was set to be the same with 40,000 interactions.

Similarly, to heuristics such as GA, SA and AC the method runs until it reaches a maximum number of iterations. We have set this parameter equality between the proposed and benchmark method to a fixed value of *i = 40*,*000* iterations. The constant decay rate for the simulated entropy probability was set to *c = 0*.*0001*, the starting probability was set to *p = 0*.*999* and variable net-odds *b* was defined by a random uniform value between (0.01, 2]. These parameters can be adjusted to improve the quality according to the number of input nodes, but the simulation results support the thesis the proposed method is resistant to variations in the input parameters under a given degree of freedom.

The sample trial length was also set equality to both samples with N = 60. We have also performed the Shapiro-Wilk test for normality to guarantee the input sequences were not skewed or distorted to any side. The T-Test was used to than verify if the results where statically significant in regards of the final best cost distance and the running time variables, with a confidence level of 95%.

We have also taken additional precautious to compare the performance in the numerical simulation. To achieve this all starting paths are randomly flushed—with a function in Python programing language—before execution begin, in order to guarantee the fairness when running and comparing all algorithms.

### Computational statistical analysis

To demonstrate the significance and accuracy of the results we have performed a series of statistical tests to guarantee they are fair. For the numerical simulations we have created 3 test cases with 2D graphs n = {20, 30 50} nodes. Each trial has a sample with length N = 60. There are three variables recorded:

- Initial Random Tour Cost: Starting total Euclidian cost for the initial path
- Best Tour Cost Found: Distance for the best candidate solution found
- Total Execution Time: Running Time until convergence.

### The method has a few requirements:

- The cost distance X can be calculated by a logarithmic utility function U
- X is a random variable defined by U(X) = Y
- Variables X and Y have Mutual Information
- Variable X is encoded by a log-normal distribution
- A path is a random sequence produced by a Bernoulli process B with probability *p*
- The paths are Hamiltonian
- The estimated value for Y is the expected solution quality

We will use a subset with N = 10 from trial with n = 50 nodes and length N = 60 to demonstrate the simulation results in Table 12.

**Table 12:** Subset of the simulation results for 50 nodes with trial length of N = 60.

50-node						
Trial N = 60	Initial Random Tour Cost		Best Tour Cost Found		Total Execution Time	
0	QA	SA	QA	SA	QA	SA
1	4584.994704	4350.530615	783.6135781	1013.45381	91.94147284	127.121441
2	4931.04388	3829.72755	1002.266343	856.714194	79.49352819	93.1328427
3	4459.545641	4285.493079	892.4679561	878.335327	67.90769484	73.4395111
4	5189.508014	4873.122362	825.1186081	986.814761	65.57152077	74.8066559
5	5303.265261	5389.224823	846.448402	1085.77975	65.92321619	72.4844698
6	4792.633045	4595.016823	1036.163983	855.195737	71.40535116	70.3046693
7	4951.587728	4711.457297	735.82339	1110.36984	61.43196151	78.8009844
8	5127.650419	4851.865628	1028.714485	974.535522	67.16026587	80.7510912
9	4213.123791	4227.131367	1006.163651	693.015657	61.80173641	75.0614487
10	4388.805181	5361.415343	887.0345646	813.130685	68.36587932	73.5915055

## Fair run and descriptive statistics

We have implemented the proposed quantitative algorithm (QA) and the Simulated Annealing (SA) heuristic in a set of trials with n = {20, 30, 50} nodes and a sample with length N = 60. To guarantee the results are fair we have analyzed the distribution with the Shapiro-Wilk test to demonstrate the sample is normally distributed. The QA and SA tests for n = 50 nodes is described below:

## Results from QA simulation: Initial random tour cost variable

The statistical description for the QA method distribution with n = 50 nodes and length N = 60 is described as follow

- Nodes in the graph (n): 50
- Sample size (N): 60
- Average ($\overset{¯}{x}$): 4679.279218
- Median: 4633.623941
- Sample Standard Deviation (σ): 348.057997
- Sum of Squares: 7147517.782
- b: 2640.163152
- Skewness: 0.543901
- Skewness Shape: Potentially Symmetrical (pval = 0.078)
- Excess kurtosis: 0.152926
- Tails Shape: Potentially Mesokurtic, normal like tails (pval = 0.802)
- P-value: 0.260661
- Outliers: 5651.239742

**Fig 30:**
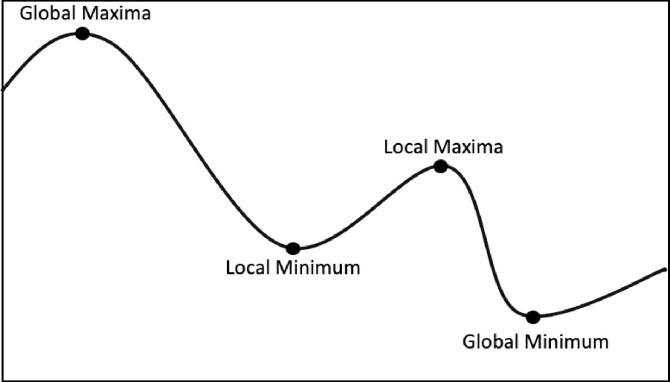
QA sample: Normal distribution for n = 50 and N = 60.

**Fig 31:**
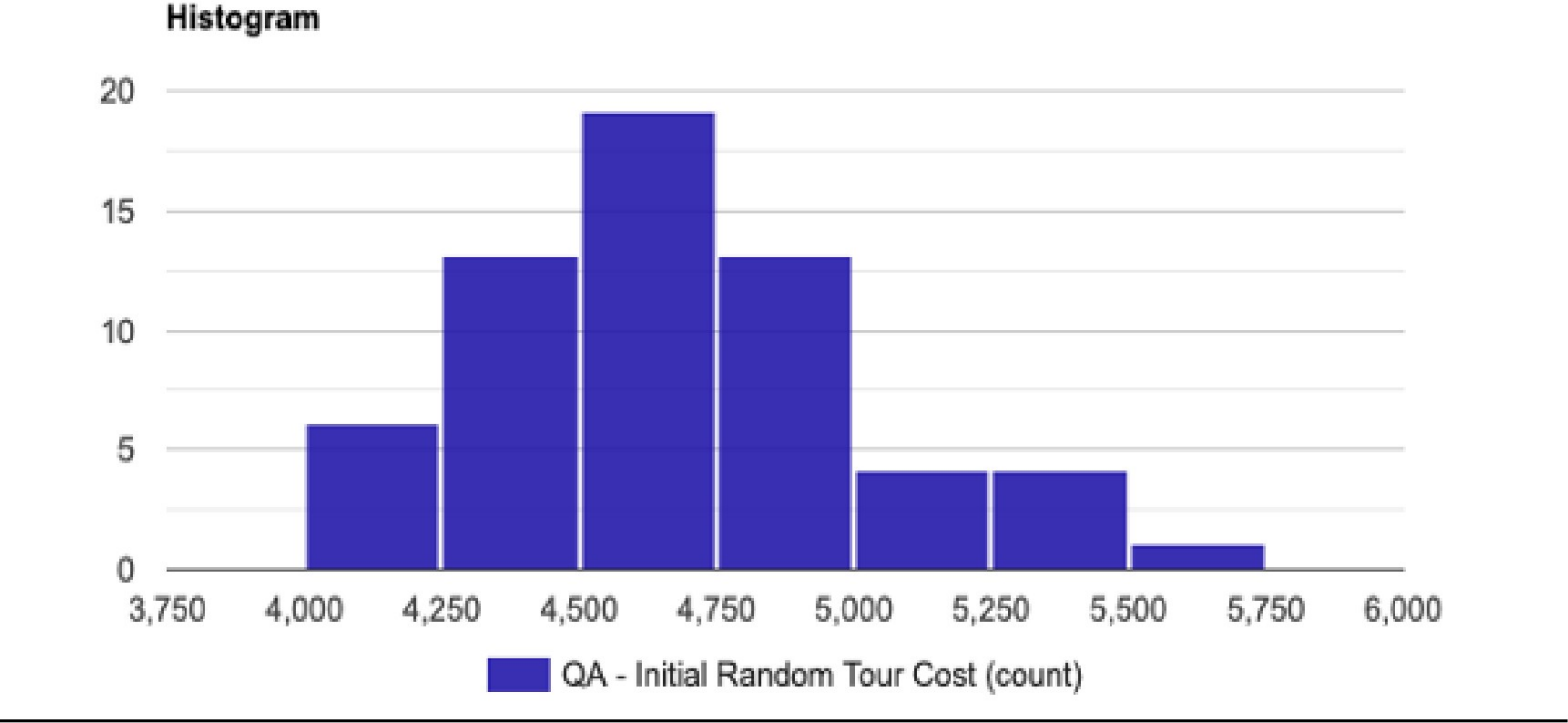
Histogram for the initial tour cost in the QA method.

**Fig 32:**
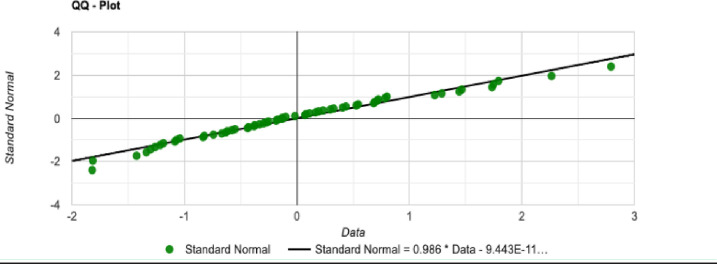
QQ-Plot for the initial tour cost in the QA method.

The normal distribution with average $\overset{¯}{x}$ = 4679.279 and standard deviation σ = 348.057 with Significance level (α): 0.05 is shown in Fig 30. The Histogram and QQ-Plot are represented in Figs 31 and 32, respectively.

### Shapiro-Wilk test, using a right-tailed normal distribution

1. H_0_ hypothesis
  Since p-value > α, we accept the H_0_. It is assumed that the data is normally distributed. In other words, the difference between the data sample and the normal distribution is not big enough to be statistically significant.
2. P-value
  p-value is 0.260661, hence, if we would reject H_0_, the chance of type1 error (rejecting a correct H_0_) would be too high: 0.2607 (26.07%). The larger the p-value, the more it supports H_0_
3. The statistics
  W is 0.975228. It is in the 95% critical value accepted range: [0.9605: 1.0000]

## Results from SA simulation: Initial random tour cost variable

The statistical description for the SA method distribution with n = 50 nodes and length N = 60 is described as follow

**Fig 33:**
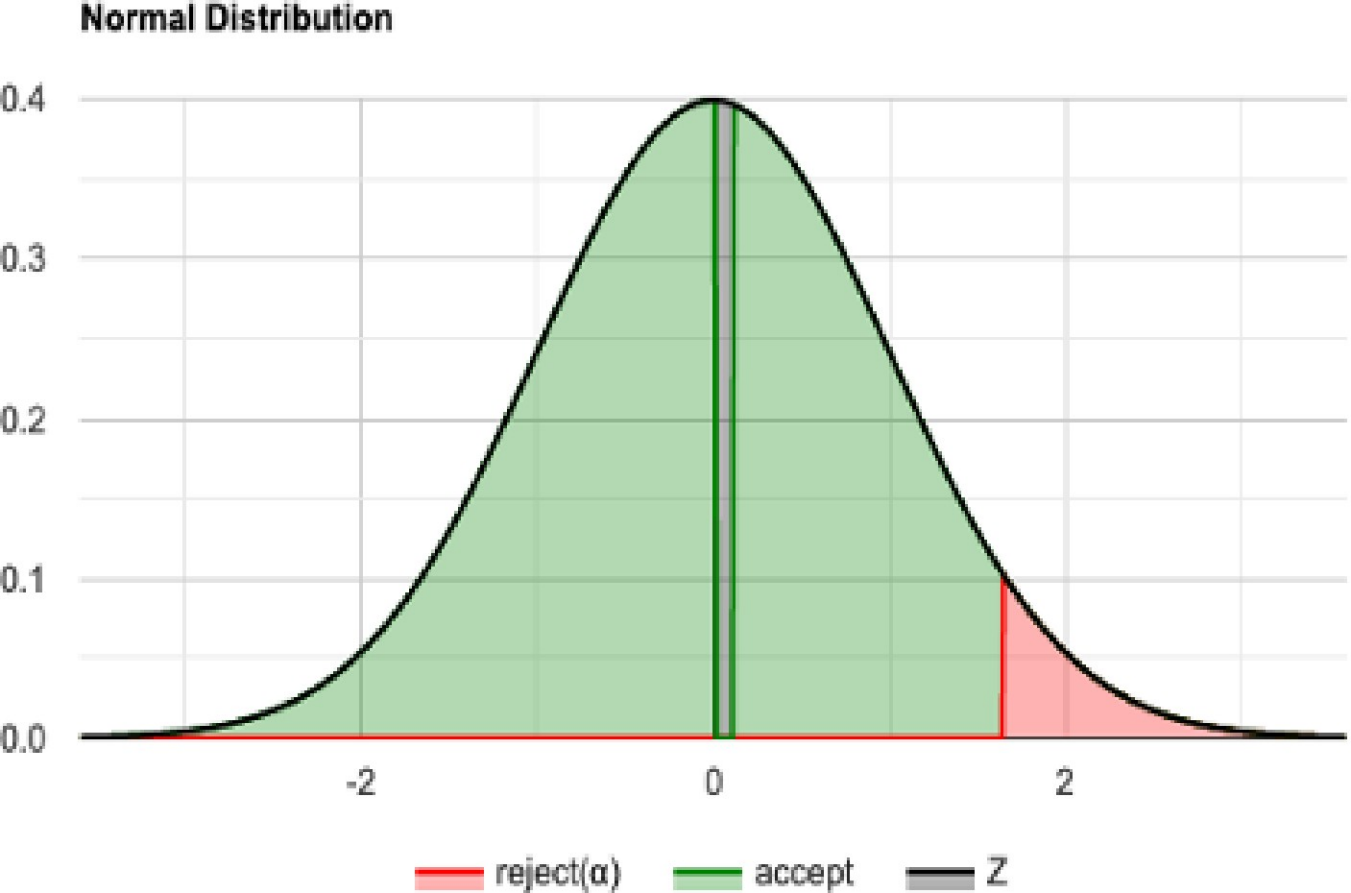
SA Sample: Normal distribution for n = 50 and N = 60.

**Fig 34:**
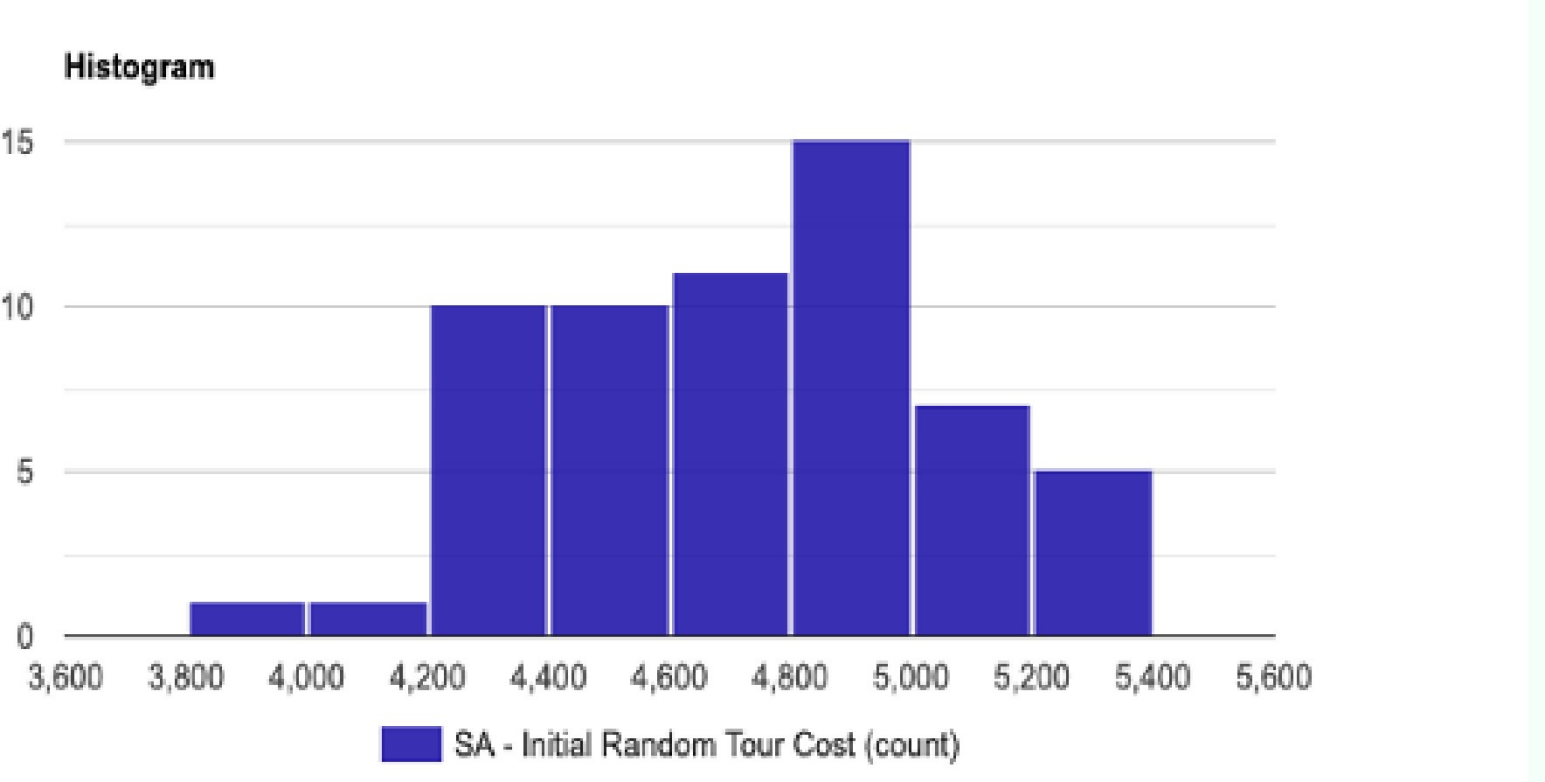
Histogram for the initial tour cost in the SA method.

**Fig 35:**
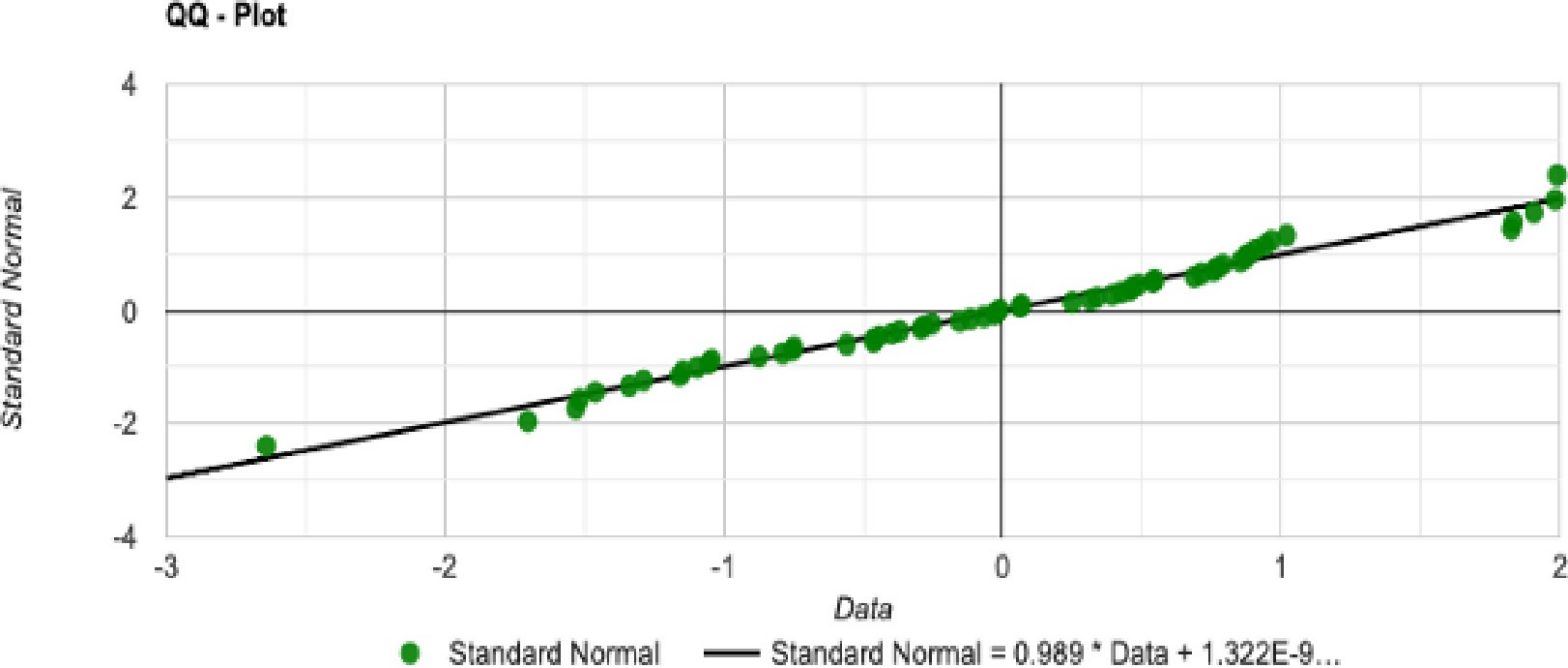
QQ-Plot for the initial tour cost in the SA method.

- Nodes in the graph (n): 50
- Sample size (N): 60
- Average ($\overset{¯}{x}$): 4719.187703
- Median: 4713.7218465000005
- Sample Standard Deviation (σ): 336.776436
- Sum of Squares: 6691683.709
- b: 2562.233720
- Skewness: -0.0726740
- Skewness Shape: Potentially Symmetrical (pval = 1.186)
- Excess kurtosis: -0.128238
- Tails Shape: Potentially Mesokurtic, normal like tails (pval = 1.167)
- P-value: 0.475660
- Outliers:

The normal distribution with average $\overset{¯}{x}$ = 4719.187 and standard deviation σ = 336.776436 with Significance level (α): 0.05 is shown in *Fig 33*. The Histogram and QQ-Plot are represented in Figs 34 and 35, respectively.

### Shapiro-Wilk test, using a right-tailed normal distribution

1. H0 hypothesis
  Since p-value > α, we accept the H0. It is assumed that the data is normally distributed. In other words, the difference between the data sample and the normal distribution is not big enough to be statistically significant.
2. P-value
  p-value is 0.475660, hence, if we would reject H0, the chance of type1 error (rejecting a correct H0) would be too high: 0.4757 (47.57%). The larger the p-value, the more it supports H0
3. The statistics
  W is 0.981075. It is in the 95% critical value accepted range: [0.9605: 1.0000]

Next we have created the boxplot graph to represent the median, average and the quartiles for QA and SA sample distributions.

### Results from QA simulation: Best tour cost found

The statistical description for the QA method distribution with n = 50 nodes and length N = 60 is described as follow

- Sample size: 60
- Median: 897.3196982
- Minimum: 700.0706523
- Maximum: 1123.628464
- First quartile: 829.287859975
- Third quartile: 971.0301233
- Interquartile Range: 141.742263325
- Outliers: none

**Fig 36:**
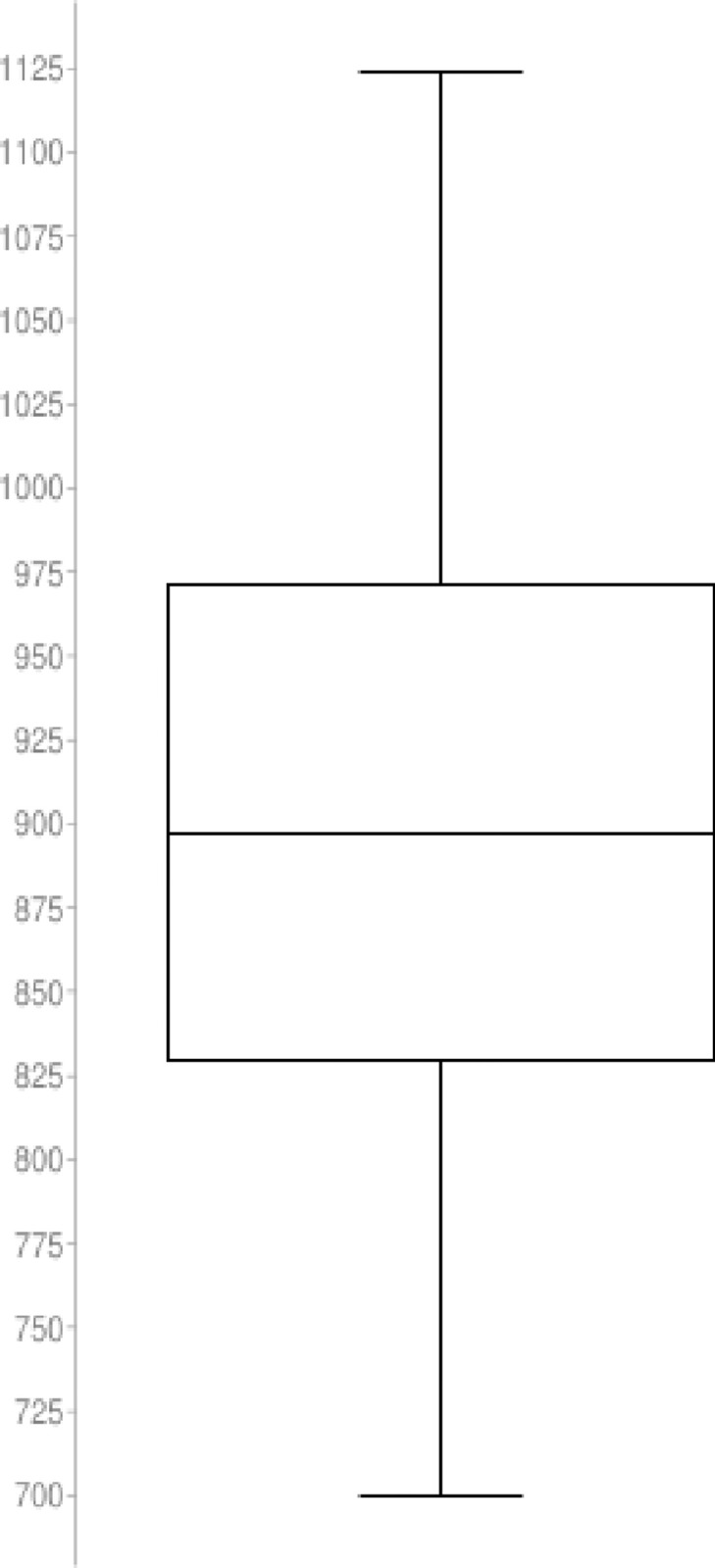
Boxplot for the QA simulation.

The boxplot for the QA distribution is illustrated in Fig 36

#### Results from SA simulation: Best tour cost found

The statistical description for the SA method distribution with n = 50 nodes and length N = 60 is described as follow

- Sample size: 60
- Median: 961.0134322
- Minimum: 679.4900154
- Maximum: 1358.45442
- First quartile: 865.4905371
- Third quartile: 1057.63755725
- Interquartile Range: 192.14702015
- Outlier: 1358.45442

**Fig 37:**
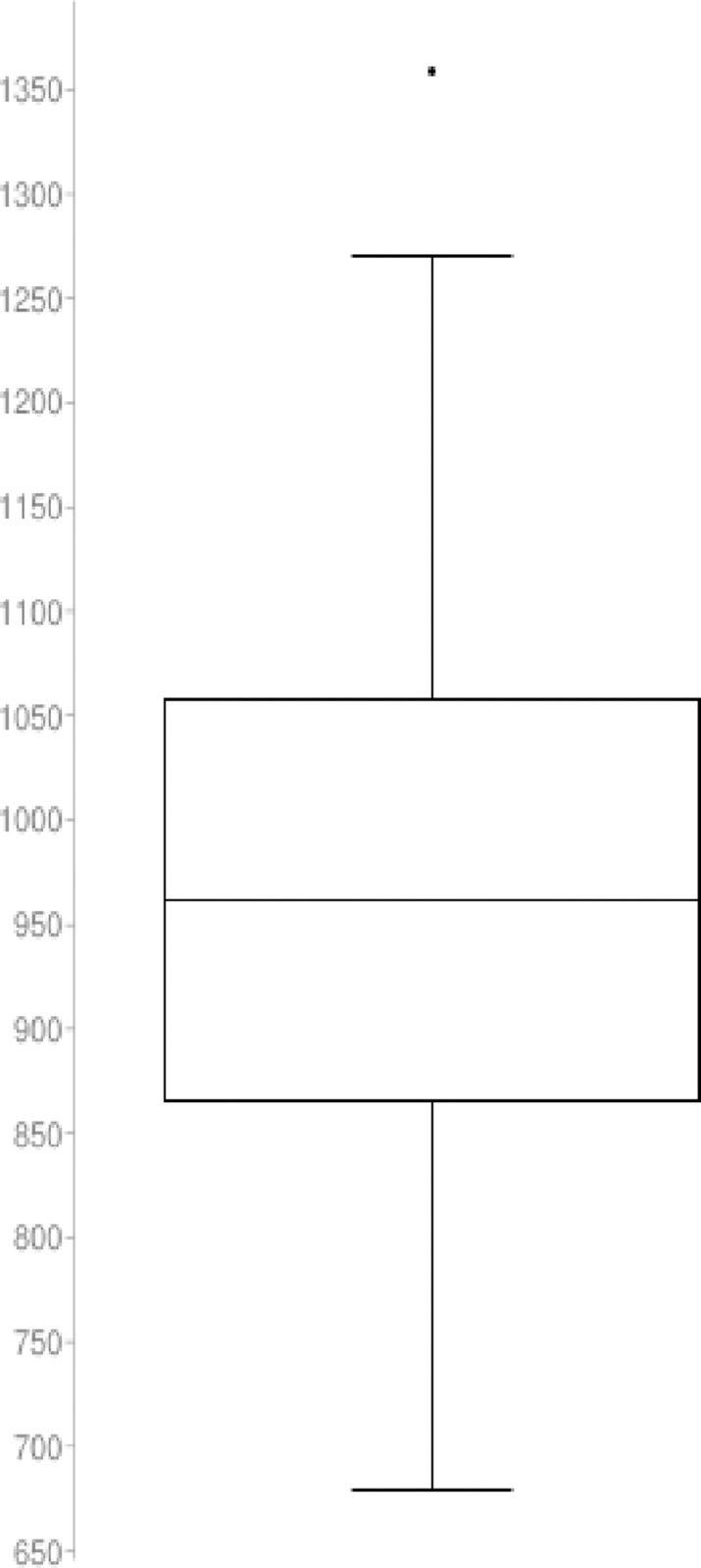
Boxplot for the SA simulation.

The boxplot for the SA distribution is illustrated in Fig 37

### Test cases and computer simulation

The test cases are a set with 20, 30 and 50 nodes. The network latency between each node is bounded by the geographic distances between the machines, made available by the computing service provider. It’s the time required to send a message over a noisy communication channel. The proposed algorithm was used to find the optimal network route to deploy a process in a cluster of machines. In *Tables 13*–*15* we present the Euclidian coordinates used to generate the candidate solutions and the cost matrix. We have provided a sample to demonstrate the results between QA and SA from the 180 trials (N = 60 for each sample). The data shows the first initial tour and the optimal solution found for each algorithm (the example data was extracted from the results produced by the simulations).

List of nodes with length N = 20 with coordinates (X, Y) in a 2D plan.

**Table 13:** Node coordinates with size n = 20.

Node	X	Y
1	288	149
2	288	129
3	270	133
4	256	141
5	256	157
6	246	157
7	236	169
8	228	169
9	228	161
10	220	169
11	212	169
12	204	169
13	196	169
14	188	169
15	196	161
16	188	145
17	172	145
18	164	145
19	156	145
20	148	145

The QA simulation results are described with four variables

- First solution path: ['1', '20', '8', '7', '17', '13', '2', '3', '16', '9', '15', '11', '14', '10', '6', '19', '4', '5', '18', '12']
- Total distance cost from the first(starting) solution: 1145.7617186058662
- Best solution Path: ['6', '5', '1', '2', '3', '4', '9', '15', '16', '17', '18', '19', '20', '14', '13', '12', '11', '10', '8', '7']
- Total distance cost for the best solution found: 332.1144088148687

List of nodes with length N = 30 with coordinates (X, Y) in a 2D plan

**Table 14:** Node coordinates with size n = 30.

Node	X	Y
1	288	149
2	288	129
3	270	133
4	256	141
5	256	157
6	246	157
7	236	169
8	228	169
9	228	161
10	220	169
11	212	169
12	204	169
13	196	169
14	188	169
15	196	161
16	188	145
17	172	145
18	164	145
19	156	145
20	148	145
21	140	145
22	148	169
23	164	169
24	172	169
25	156	169
26	140	169
27	132	169
28	124	169
29	116	161
30	104	153

The QA simulation results are described with four variables

- First solution path: ['20', '24', '23', '9', '2', '6', '21', '15', '27', '1', '11', '14', '29', '25', '5', '28', '13', '7', '18', '22', '26', '4', '19', '12', '3', '17', '10', '30', '8', '16']
- Total distance cost from the first(starting) solution: 2114.8616643887417
- Best solution Path: ['4', '9', '12', '13', '14', '24', '23', '25', '22', '26', '27', '28', '29', '30', '21', '20', '19', '18', '17', '16', '15', '11', '10', '8', '7', '6', '5', '1', '2', '3']
- Total distance cost for the best solution found: 423.26765018746386

List of nodes with length N = 50 with coordinates (X, Y) in a 2D plan

**Table 15:** Node coordinates with size n = 50.

Node	X	Y
1	288	149
2	288	129
3	270	133
4	256	141
5	256	157
6	246	157
7	236	169
8	228	169
9	228	161
10	220	169
11	212	169
12	204	169
13	196	169
14	188	169
15	196	161
16	188	145
17	172	145
18	164	145
19	156	145
20	148	145
21	140	145
22	148	169
23	164	169
24	172	169
25	156	169
26	140	169
27	132	169
28	124	169
29	116	161
30	104	153
31	104	161
32	104	169
33	90	165
34	80	157
35	64	157
36	64	165
37	56	169
38	56	161
39	56	153
40	56	145
41	56	137
42	56	129
43	56	121
44	40	121
45	40	129
46	40	137
47	40	145
48	40	153
49	40	161
50	40	169

The QA simulation results are described with four variables

- First solution path: ['38', '48', '39', '40', '9', '26', '28', '29', '33', '43', '41', '14', '12', '23', '1', '17', '20', '5', '49', '7', '35', '22', '18', '31', '6', '19', '13', '36', '2', '16', '42', '15', '4', '32', '25', '46', '8', '47', '50', '27', '37', '24', '34', '3', '10', '30', '45', '21', '44', '11']
- Total distance cost from the first(starting) solution: 4919.680538441
- Best solution Path: ['49', '48', '47', '41', '42', '43', '44', '45', '46', '40', '39', '38', '37', '36', '35', '34', '33', '32', '31', '30', '29', '21', '20', '19', '18', '17', '16', '9', '5', '1', '2', '3', '4', '6', '7', '8', '10', '11', '12', '15', '13', '14', '24', '23', '25', '22', '26', '27', '28', '50']

Total distance cost for the best solution found: 700.0706523445637

**Fig 38:**
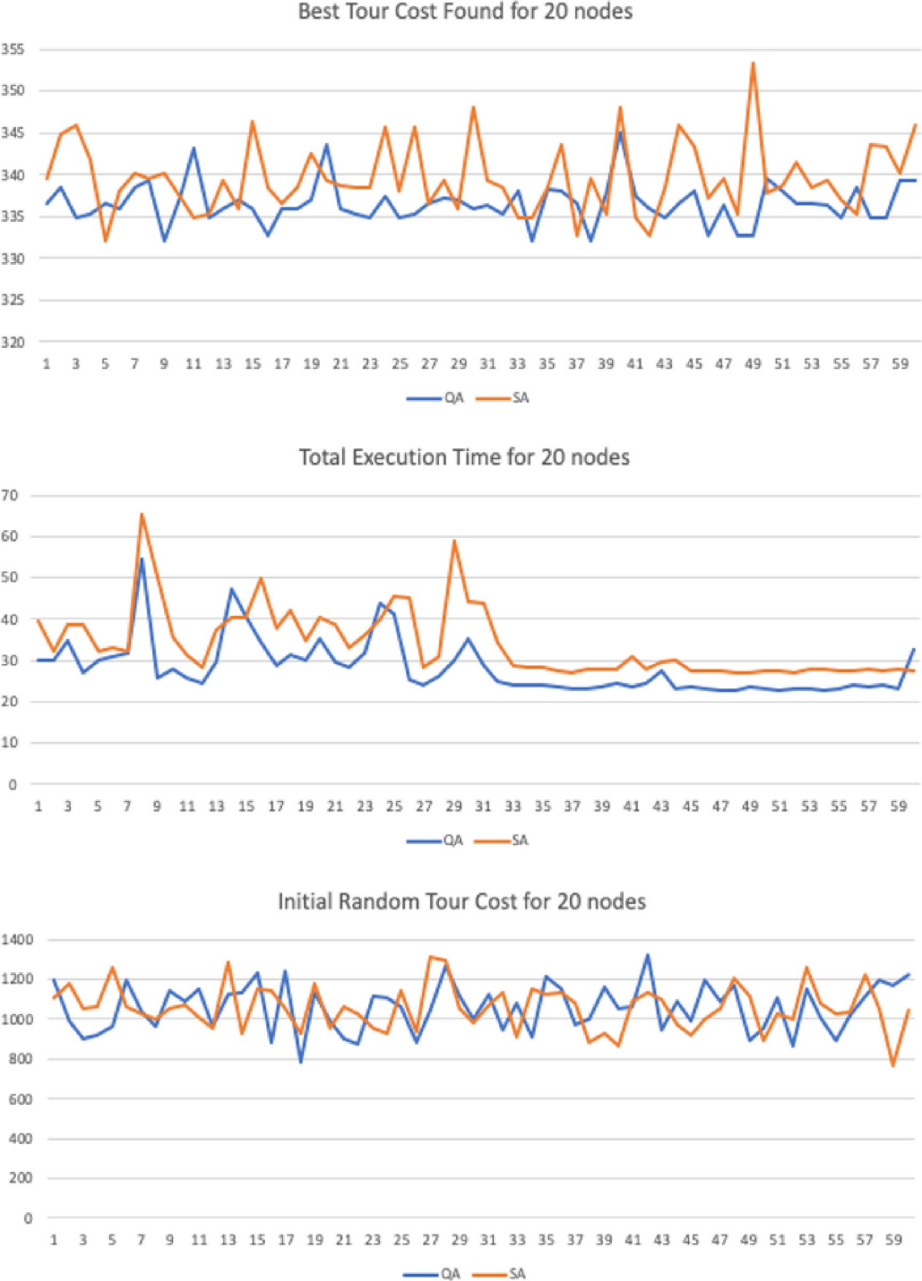
Chart with results for test cases with n = 20 nodes with sample size N = 60.

**Fig 39:**
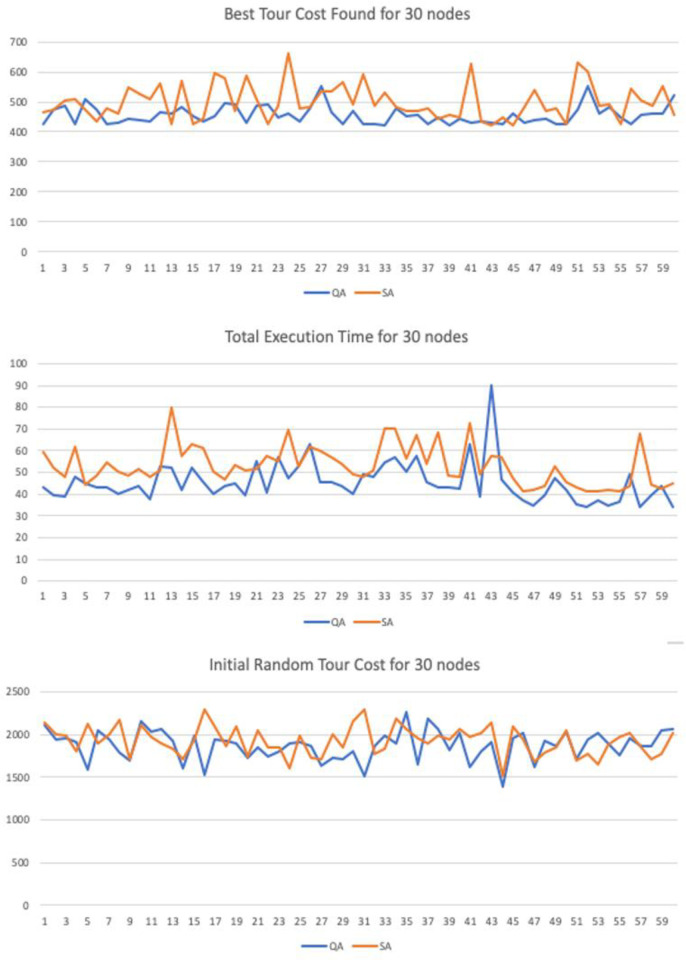
Chart with results for test cases with n = 30 nodes with sample size N = 60.

**Fig 40:**
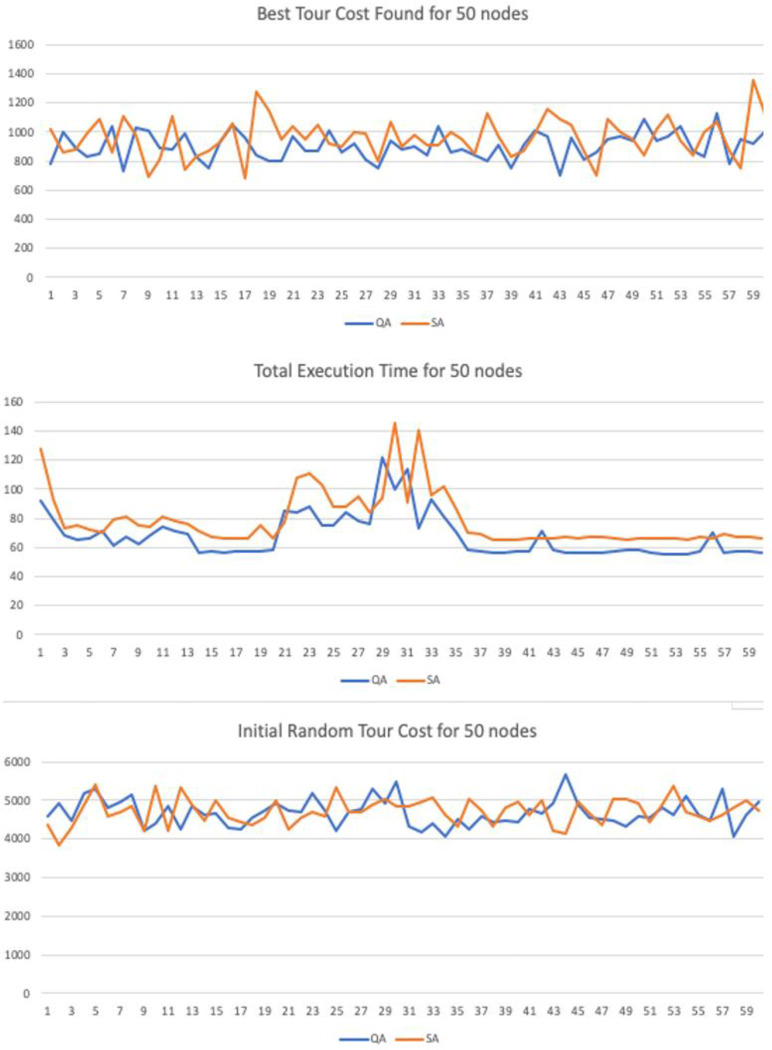
Chart with results for test cases with n = 50 nodes with sample size N = 60.

In Figs 38, 39 and 40 we have the line charts with the results for each test case. The figures demonstrate that the QA algorithms produces expected results with smaller cost and best quality in average than the benchmark SA method with smaller time requirements. In all cases the initial path loaded from the input files was randomly flushed before the algorithm execution to guarantee the results were not biased.

*Tables 16*–*21* compares the results between the proposed algorithm and the heurists methods. They demonstrate that the result generated by the QA algorithm achieves solutions with better quality than the benchmark SA heuristic algorithm, by finding optimal solutions with smaller total cost and lesser running time. The QA algorithm converges more quickly than the 2opt algorithm for large instances of the problem. The results suggest the proposed Quantitative Algorithm is likely to perform better and generate consistent results as other traditional heuristics methods applied to the TSP. Additionally it provides an Information Theory modelling for Computationally Complex domains such as the NP class of problems in Turing Machines.

**Table 16:** T-test for n = 20 nodes and N = 60 trials–total distance cost variable.

t-Test: Two-Sample Assuming Unequal Variances		
Cost Variable		
	*QA*	*SA*
Mean	336.552862	339.645729
Variance	6.35307736	18.1161823
Observations	60	60
Hypothesized Mean Difference	0	
Df	96	
t Stat	-4.8431338	
P(T< = t) one-tail	2.4468E-06	
t Critical one-tail	1.66088144	
P(T< = t) two-tail	4.8935E-06	
t Critical two-tail	1.98498431	

The result is significant at *p* < .05.

The 60 iterations who executed the QA algorithm (*M* = 336.552, *Var* = 6.353) compared to the 60 iterations in the SA algorithm (*M* = 339.645, *Var* = 18.116) demonstrated significantly better cost reduction, with value *p* = 4.8935E-06.

**Table 17:** T-test for n = 20 nodes and N = 60 trials—execution time variable.

t-Test: Two-Sample Assuming Unequal Variances		
Execution Time Variable		
	*QA*	*SA*
Mean	28.1821012	34.06591929
Variance	43.5437993	69.06678708
Observations	60	60
Hypothesized Mean Difference	0	
df	112	
t Stat	-4.2948228	
P(T< = t) one-tail	1.8689E-05	
t Critical one-tail	1.65857263	
P(T< = t) two-tail	3.7379E-05	
t Critical two-tail	1.98137181	

The result is significant at *p* < .05.

The 60 iterations who executed the QA algorithm (*M* = 28.182, *Var* = 43.543) compared to the 60 iterations in the SA algorithm (*M* = 34.065, *Var* = 69.066) demonstrated significantly better time to complete execution, with value *p* = 3.7379E-05.

**Table 18:** T-test for n = 30 nodes and N = 60 trials–total distance cost variable.

t-Test: Two-Sample Assuming Unequal Variances		
Cost Variable		
	*QA*	*SA*
Mean	455.9177842	500.1505
Variance	917.1905283	3396.48698
Observations	60	60
Hypothesized Mean Difference	0	
df	89	
t Stat	-5.216694386	
P(T< = t) one-tail	5.88396E-07	
t Critical one-tail	1.662155326	
P(T< = t) two-tail	1.17679E-06	
t Critical two-tail	1.9869787	

The result is significant at *p* < .05.

The 60 iterations who executed the QA algorithm (*M* = 455.917, *Var* = 917.190) compared to the 60 iterations in the SA algorithm (*M* = 500.150, *Var* = 3396.486) demonstrated significantly better cost reduction, with value *p* = 1.17679E-06.

**Table 19:** T-test for n = 30 nodes and N = 60 trials—execution time variable.

t-Test: Two-Sample Assuming Unequal Variances		
Execution Time Variable		
	*QA*	*SA*
Mean	45.3019303	53.1616159
Variance	83.6019137	82.7521257
Observations	60	60
Hypothesized Mean Difference	0	
df	118	
t Stat	-4.7202404	
P(T< = t) one-tail	3.2683E-06	
t Critical one-tail	1.65786952	
P(T< = t) two-tail	6.5365E-06	
t Critical two-tail	1.98027225	

The result is significant at *p* < .05.

The 60 iterations who executed the QA algorithm (*M* = 45.301, *Var* = 83.601) compared to the 60 iterations in the SA algorithm (*M* = 53.161, *Var* = 82.752) demonstrated significantly better time to complete execution, with value *p* = 6.5365E-06.

**Table 20:** T-test for n = 50 nodes and N = 60 trials–total distance cost variable.

t-Test: Two-Sample Assuming Unequal Variances		
Cost Variable		
	*QA*	*SA*
Mean	901.7845936	960.3116942
Variance	8917.028193	18189.17941
Observations	60	60
Hypothesized Mean Difference	0	
df	106	
t Stat	-2.753583529	
P(T< = t) one-tail	0.003468798	
t Critical one-tail	1.659356034	
P(T< = t) two-tail	0.006937597	
t Critical two-tail	1.982597262	

The result is significant at *p* < .05.

The 60 iterations who executed the QA algorithm (*M* = 901.784, *Var* = 8917.028) compared to the 60 iterations in the SA algorithm (*M* = 960.311, *Var* = 18189.179) demonstrated significantly better cost reduction, with value *p* = 0.006937597.

**Table 21:** T-test for n = 50 nodes and N = 60 trials—execution time variable.

t-Test: Two-Sample Assuming Unequal Variances		
Execution Time Variable		
	*QA*	*SA*
Mean	67.6962824	78.4087974
Variance	215.812669	334.607097
Observations	60	60
Hypothesized Mean Difference	0	
df	113	
t Stat	-3.5368778	
P(T< = t) one-tail	0.00029419	
t Critical one-tail	1.65845022	
P(T< = t) two-tail	0.00058839	
t Critical two-tail	1.98118036	

The result is significant at *p* < .05.

The 60 iterations who executed the QA algorithm (*M* = 67.696, *Var* = 215.812) compared to the 60 iterations in the SA algorithm (*M* = 78.408, *Var* = 334.607) demonstrated significantly better time to complete execution, with value *p* = 0.00058839.

In the Appendix sections we have the Tables 22, 23 and 24 for the 3 test cases with nodes n = {20, 30, 50}, with the trial input variables for best final tour cost, total execution time and the initial tour cost for a sample length of N = 60. In Tables 19–21 we have the t-test p-value for each test case for the cost and execution time variable. *Tables 16 and 17* demonstrate the T-test calculation for 2 independent means for n = 20.

**Table 22:** Execution results for 20 nodes (n = 20) with samples size N = 60.

20-node						
Trial N = 60	Initial Random Tour Cost		Best Tour Cost Found		Total Execution Time	
0	QA	SA	QA	SA	QA	SA
1	1195.634925	1108.393775	336.620892	339.439482	30.12948538	39.6077362
2	986.9352715	1177.732299	338.5615264	344.758321	30.26399596	32.3809037
3	900.0458995	1054.1641	334.8573413	345.966652	34.91276873	38.7632731
4	916.5463351	1057.927051	335.3011739	341.855037	27.23480984	38.4763941
5	966.1553232	1253.494224	336.620892	332.114409	29.91621914	32.1631082
6	1194.232179	1064.387485	335.8914965	338.044106	31.12498264	33.1915936
7	1036.191977	1029.253756	338.5615264	340.135251	31.81935215	32.060559
8	963.2410995	999.9887981	339.3638244	339.439482	54.49795967	65.4112282
9	1145.761719	1050.78347	332.1144088	340.059594	25.79352437	50.170809
10	1085.090655	1073.381069	337.2219503	337.316661	27.91745513	35.5128518
11	1147.417441	1012.068389	343.0294931	334.857341	25.94175636	31.4301689
12	965.8459042	958.2459652	334.8573413	335.301174	24.29773869	28.3900081
13	1128.489942	1283.781151	335.9022323	339.231552	29.54066219	37.4386245
14	1131.818667	928.6586146	337.0140207	335.902232	47.45874454	40.282868
15	1229.658241	1153.404679	335.9022323	346.41498	40.86180719	40.4422965
16	884.1357153	1138.13307	332.7154672	338.561526	34.48849958	49.9608527
17	1235.918025	1052.377384	335.9022323	336.620892	28.82108637	37.7635394
18	783.6900544	929.1934876	335.8914965	338.561526	31.23878077	42.0187519
19	1130.320419	1174.181378	337.0140207	342.439171	30.00009195	34.7658014
20	989.3865301	951.2563694	343.5134562	339.363824	35.36053606	40.5407622
21	904.2377617	1063.30281	335.9022323	338.741826	29.65111817	38.7390394
22	869.7243065	1029.595623	335.3011739	338.561526	28.38350387	32.86863
23	1116.176649	953.0821731	334.8573413	338.561526	31.87757207	36.2553472
24	1110.200886	931.6046509	337.3166611	345.685585	43.94790548	40.1406831
25	1064.184077	1139.258709	334.8573413	338.119763	41.20329418	45.7096829
26	887.1252702	940.9695223	335.3011739	345.685585	25.25219875	45.1240136
27	1040.447342	1308.425282	336.4925548	336.620892	23.90627663	28.4887706
28	1263.036989	1297.030551	337.2219503	339.231552	26.12993897	31.0484482
29	1115.022094	1055.582523	337.0140207	335.902232	29.95108272	58.9338429
30	998.9318306	981.3689839	335.9022323	348.044562	35.33063453	44.3207047
31	1127.712215	1066.313009	336.4129623	339.342884	28.56060964	43.9690071
32	945.3399049	1133.277072	335.3011739	338.561526	25.08187038	34.241621
33	1079.60359	913.4123438	338.0441064	334.857341	23.84379763	28.6076721
34	913.3758632	1151.462443	332.1144088	334.932998	24.00100185	28.4874175
35	1210.249512	1120.714135	338.3112039	338.561526	24.2204857	28.3244191
36	1147.723302	1130.778705	338.0441064	343.649497	23.43391117	27.3482798
37	971.3903556	1076.686391	336.620892	332.715467	23.04826988	27.2579731
38	998.2103312	887.1398159	332.1144088	339.439482	23.30005102	27.9801567
39	1162.280089	924.1853956	337.9177195	335.301174	23.55994665	28.0173068
40	1054.008142	869.5783019	345.095262	348.044562	24.4069206	28.0580326
41	1062.958346	1092.610068	337.3166611	334.857341	23.58406409	30.9849236
42	1316.550471	1132.915517	335.9022323	332.715467	24.49907442	27.7148466
43	944.5629418	1096.900034	334.8573413	338.561526	27.42394148	29.689707
44	1090.841136	973.9952212	336.620892	345.879822	23.22344759	30.1561858
45	985.9883167	915.8312807	338.0441064	343.428117	23.68698814	27.5134631
46	1193.337578	1001.967642	332.7154672	337.22195	23.15058773	27.6503922
47	1085.046538	1056.207841	336.4129623	339.439482	22.89809065	27.6979532
48	1170.592904	1204.896611	332.7154672	335.301174	22.811833	27.1470865
49	888.3716219	1119.310235	332.7154672	353.222205	23.53460078	27.1795784
50	957.744169	896.1779984	339.4394815	337.917719	22.97475352	27.4758247
51	1105.010107	1023.615603	338.0441064	338.710086	22.86918458	27.2797519
52	868.7296417	1001.961386	336.4925548	341.484758	23.09712953	27.1193013
53	1147.110698	1261.361139	336.620892	338.561526	23.20182619	27.8441451
54	1007.496906	1076.417337	336.4129623	339.231552	22.91703718	27.7154546
55	888.3254017	1028.532428	334.8573413	337.014021	23.08894057	27.652091
56	1015.389387	1037.982865	338.5615264	335.301174	24.19610564	27.4061656
57	1118.083211	1218.690552	334.8573413	343.500897	23.50864635	27.9921575
58	1196.871246	1056.936272	334.8573413	343.428117	23.84533973	27.6875045
59	1172.5209	765.331396	339.3638244	340.059594	23.19705539	27.9977026
60	1225.40291	1041.148332	339.3638244	345.962516	32.50677825	27.3577434

**Table 23:** Execution results for 30 nodes (n = 30) with samples size N = 60.

30-node						
Trial N = 60	Initial Random Tour Cost		Best Tour Cost Found		Total Execution Time	
0	QA	SA	QA	SA	QA	SA
1	2114.861664	2138.958342	424.5873682	464.700899	42.98706438	59.0712779
2	1941.575116	2007.049171	475.6796776	472.590934	39.46565883	52.120306
3	1950.142483	1989.063029	488.989852	504.453757	38.60825704	47.8920127
4	1902.474244	1796.927917	424.3794386	506.801158	48.13539106	61.4913984
5	1580.54924	2121.678062	510.318469	472.980497	44.92054756	44.2866884
6	2040.501524	1887.043741	474.6886065	433.933129	43.33821167	48.5092938
7	1933.189429	2005.284134	426.0105826	478.033092	43.31067901	54.8387858
8	1791.060214	2174.057473	429.9694289	462.536112	40.27118746	50.435126
9	1697.688572	1712.42956	443.4357473	548.033561	41.870426	48.4660826
10	2159.279821	2101.511052	439.9631361	526.162767	43.91304872	51.6482356
11	2037.032939	1964.471053	436.517103	507.74012	37.68801916	48.1148758
12	2057.901751	1892.015046	467.0236214	562.704654	52.46498072	51.1041011
13	1930.750408	1831.163179	460.014352	426.528003	52.34054281	79.7551805
14	1596.34284	1709.164989	484.0191023	571.764133	41.91429676	57.815061
15	1989.991866	1932.746628	453.6891305	428.101727	52.06071529	63.1093559
16	1532.819374	2294.07567	432.8197583	443.083125	45.44243764	61.380042
17	1933.611183	2091.996596	453.529067	595.347046	40.35533853	50.0510586
18	1928.551184	1860.347264	495.9653506	580.368162	43.90844072	46.7043453
19	1892.952203	2098.647235	491.0938606	468.450763	45.20533055	53.5513851
20	1727.389612	1746.213002	428.8730412	587.027872	39.56974309	50.744991
21	1840.460231	2052.640373	486.8539675	510.278944	55.44163731	51.324384
22	1734.505095	1843.716782	491.0156727	427.122371	40.87608984	57.7772876
23	1804.725607	1840.418372	447.2951117	486.01134	57.19451948	55.1076989
24	1900.969989	1597.14205	460.1507211	662.168972	47.51163171	69.8538226
25	1902.159287	1982.433073	433.0617383	477.980165	52.9507134	52.5828033
26	1861.379571	1732.215165	478.1275476	481.88559	63.18074776	61.506958
27	1632.125705	1705.527663	551.6573018	534.398358	45.71926259	59.901718
28	1724.804881	2004.34791	464.0476771	533.121258	45.68037277	57.0611448
29	1711.276107	1851.488894	426.6765623	565.016049	43.55173205	53.7347876
30	1798.24361	2147.552334	467.8403795	491.540871	39.89643796	48.881184
31	1505.540858	2286.236266	427.122371	592.196033	48.98996233	48.1386252
32	1868.889925	1772.226931	427.4357473	488.765595	47.68942928	50.6868412
33	1986.199157	1831.914237	423.8687085	530.861945	54.60683943	70.0300942
34	1893.774487	2191.409802	476.6245181	482.210099	56.77093291	69.9386231
35	2254.034678	2057.794281	450.6222049	470.823818	50.39574996	56.2931122
36	1641.972013	1951.754928	456.9229002	470.823818	57.52063273	67.2678736
37	2190.641527	1892.120281	427.1980282	479.691631	45.58968535	54.0853191
38	2059.180754	1991.897847	446.6225652	443.410875	43.33406437	68.1481031
39	1816.734006	1938.160932	423.2676502	457.649743	43.01746808	48.3186617
40	2009.362159	2057.941367	442.9039608	449.980838	42.63856604	48.0714487
41	1619.209653	1977.29523	430.5704872	625.975132	63.20465673	72.4023792
42	1803.072488	2008.969722	436.3291255	434.210099	38.81750628	49.0113726
43	1905.327122	2139.748939	431.6159736	422.823818	90.17018865	57.4507498
44	1396.063861	1510.800171	427.122371	446.937846	46.57496965	56.7508254
45	1961.970303	2085.66334	461.2238488	423.868709	40.88945298	47.0390539
46	2016.906558	1945.01613	431.6159736	482.392057	37.00943786	41.2652409
47	1615.747074	1679.767432	438.5243978	540.930956	34.94234454	41.9965412
48	1919.853209	1791.446699	442.7060685	468.628522	39.62350488	43.5543638
49	1864.485907	1841.077381	427.122371	478.851995	47.30976915	52.8924325
50	2033.030066	2044.268536	427.1980282	427.122371	41.87760103	45.4800351
51	1717.798426	1690.966193	474.7739314	629.184876	35.09427462	42.9087137
52	1943.528232	1778.978193	553.2831374	599.182846	34.25907436	41.3755334
53	2011.609188	1652.077927	460.8365081	489.159339	36.82620546	41.3364099
54	1893.324832	1873.394732	483.4554998	491.107552	34.69045473	41.6829564
55	1761.65708	1971.263332	448.7644393	426.010583	36.35978929	41.3863896
56	1948.645928	2020.716704	428.0260698	542.400253	48.97381794	43.9572916
57	1867.947796	1850.588061	455.8265125	505.167828	34.33525243	67.9172457
58	1858.698459	1717.237261	461.1244452	485.823316	39.20625309	44.2034129
59	2046.855774	1778.212757	461.5046079	554.4155	43.44987886	42.5260492
60	2054.476294	2022.162447	522.5612263	457.556595	34.17459443	44.7598619

**Table 24:** Execution results for 50 nodes (n = 50) with samples size N = 60.

50-node						
Trial N = 60	Initial Random Tour Cost		Best Tour Cost Found		Total Execution Time	
0	QA	SA	QA	SA	QA	SA
1	4584.994704	4350.530615	783.6135781	1013.45381	91.94147284	127.121441
2	4931.04388	3829.72755	1002.266343	856.714194	79.49352819	93.1328427
3	4459.545641	4285.493079	892.4679561	878.335327	67.90769484	73.4395111
4	5189.508014	4873.122362	825.1186081	986.814761	65.57152077	74.8066559
5	5303.265261	5389.224823	846.448402	1085.77975	65.92321619	72.4844698
6	4792.633045	4595.016823	1036.163983	855.195737	71.40535116	70.3046693
7	4951.587728	4711.457297	735.82339	1110.36984	61.43196151	78.8009844
8	5127.650419	4851.865628	1028.714485	974.535522	67.16026587	80.7510912
9	4213.123791	4227.131367	1006.163651	693.015657	61.80173641	75.0614487
10	4388.805181	5361.415343	887.0345646	813.130685	68.36587932	73.5915055
11	4862.966377	4203.513913	877.8178844	1104.47353	73.62834893	81.1984111
12	4265.85426	5334.298362	986.2523487	742.17075	70.93162192	78.2357104
13	4830.792434	4901.318711	826.6711289	827.602189	68.93372081	76.0991019
14	4619.684576	4464.911989	749.4744233	871.360054	56.4622385	71.1095197
15	4672.945496	5012.768994	930.1932444	930.647017	57.45928662	66.9842984
16	4301.693121	4563.371791	1048.809952	1059.51057	56.40269137	66.0322041
17	4240.292119	4453.55102	960.9380585	679.490015	57.05427107	66.3409535
18	4546.900924	4368.046961	837.1380532	1269.71181	57.57915765	66.263964
19	4736.784109	4563.713271	803.5690309	1149.69257	57.39472165	75.1020043
20	4927.370618	4985.626756	802.0707333	949.201069	58.56337916	66.5018131
21	4745.962526	4268.210716	971.2161657	1035.84231	84.97886224	77.278123
22	4704.060283	4530.930051	871.0047479	946.415563	83.75003637	107.235943
23	5181.527463	4709.96614	869.6858203	1049.75812	88.00241065	110.964508
24	4718.091802	4570.219583	1003.026828	917.555919	74.77390515	102.335962
25	4226.097192	5336.282205	863.4354307	897.940395	74.88039926	88.0454088
26	4706.978399	4680.602383	919.2626516	999.01329	84.28058593	87.6437221
27	4782.240302	4696.840091	805.5766865	988.048877	77.9414429	94.365899
28	5287.422498	4883.631664	746.8961081	797.72096	76.3188141	84.3477808
29	4916.756496	5036.485112	934.4805999	1063.60839	121.2328974	94.1907275
30	5467.182912	4862.394357	881.0823054	898.962836	99.58652921	145.672337
31	4306.529303	4833.371655	902.1714403	982.659335	113.7672365	90.4546303
32	4183.027842	4974.432819	838.7087408	909.242445	72.86395342	140.711226
33	4392.939578	5062.464887	1037.318628	912.246168	93.25543078	96.0741311
34	4047.530913	4624.516897	855.2813664	997.411612	80.5475141	101.864948
35	4529.583132	4333.230947	877.4782972	948.148568	70.2493321	85.4985131
36	4255.799236	5016.586688	844.0019669	851.668489	58.39587275	69.6642768
37	4591.115727	4742.024106	801.0967517	1122.03704	57.4895984	68.7700118
38	4446.710076	4328.66242	908.2987592	972.825796	56.3946521	65.6056279
39	4463.651251	4803.549729	750.8183686	831.071231	55.89729563	65.1255863
40	4420.655007	4951.243552	909.1278545	866.473154	57.14852512	65.213147
41	4760.124614	4634.478576	1011.834532	987.252055	57.32113831	66.0356184
42	4644.224719	4979.032088	964.2360348	1157.92977	71.29050156	66.2798815
43	4919.680538	4208.101093	700.0706523	1085.31132	57.79070051	66.251104
44	5651.239742	4145.447347	955.4124378	1052.01851	56.53862412	66.9480465
45	4868.697543	4959.720826	813.4887026	872.689612	56.64083376	66.3630462
46	4548.266669	4667.805502	857.9641925	698.448853	56.46022322	67.1403534
47	4526.748229	4364.07524	944.2137708	1083.92177	56.08612165	67.1104689
48	4477.883901	5024.492103	972.2009554	999.856492	57.63071399	66.3520515
49	4317.125097	5044.001873	933.6392304	948.603177	58.23602575	65.5457691
50	4575.872243	4903.155907	1087.920943	839.863471	57.96136195	66.182039
51	4563.047938	4424.909203	934.2368709	1019.90042	56.72733254	65.8799768
52	4820.853628	4875.868205	970.4719961	1112.21415	55.71390442	66.183776
53	4615.052748	5386.727246	1034.389805	939.401344	55.77318577	65.8460782
54	5105.564284	4715.986396	873.7197202	836.813936	55.55562469	65.2542301
55	4633.267827	4585.63924	825.4093226	996.826956	57.06582138	66.729305
56	4487.584383	4467.312127	1123.628464	1071.80932	70.06335915	66.4194826
57	5282.957016	4620.83047	779.0921158	865.162998	56.55935544	69.5257076
58	4045.585905	4825.07628	944.2176677	747.969855	56.93291826	67.0270058
59	4633.980055	5006.414463	918.037855	1358.45442	57.70211316	66.7727485
60	4957.692367	4740.435311	1006.171011	1104.39786	56.55972443	66.2560434

*Tables 18* and *19* demonstrate the T-test calculation for 2 independent means for n = 30. *Tables 20* and *21* demonstrate the T-test calculation for 2 independent means for n = 50.

These findings can be expanded to other complex problems and it scales linearly to the search space sample. The proposed method is also resistant to the time and space constraints and it has a constant number of maximum iterations. In this paper we have introduced a new interpretation for the entropy rate for a binary program that implements a given NP problem.

The results can be used by many real-world applications such as the optimization of routing messages over a network and the orchestration of services across a distributed system such as provided by Cloud Computing environments and micro-services-oriented architectures. Besides it is also a future reference on the subject of Information Theory, Computational Complex Theory and Logarithmic utility in optimization routing, deployment, scheduling and planning. The research demonstrated that the proposed concepts have statistically significant results with better solution quality in tour planning. The model provides a new interpretation of entropy in problems encoded in Turing Machines and has the potential to change the traditional interpretation of the limits of Computing Theory.

The results are statistically significant (with p-value < 0.05), and we can conclude the proposed algorithm has better solution quality with reduced computational requirements and better cost improvement.

### Statistical analysis of the test cases

In order to test the performance in solving the TSP we have created trials with sample length N = 60 for each test case with different number of nodes n = {10, 30, 50} and then compared the results obtained with a benchmark heuristic (SA) and the proposed algorithm (QA).

Table 25 demonstrate the two-tailed t-test for two independent samples of costs and times measurements with Significance Level of 0.05. This is a two-sided test for the null hypothesis with two independent means have the identical expected value. This test measures if the average expected cost and running time value differs significantly across the measured samples. If the p-value is small than the significance level of 0.05 (5%) then we can reject the null hypothesis of equal average means.

**Table 25:** Comparison matrix for the two-tailed t-test independent means p-values for the test cases with nodes with n = 20, 30, 50 and sample size N = 60.

	**20-node**	**30-node**	**50-node**
**Cost variable t-test for N = 60**	**QA-SA.**	**QA-SA.**	**QA-SA.**
**p-value**	4.89351E-06	1.17679E-06	0.0069376
	**20-node**	**30-node**	**50-node**
**Time variable t-test for N = 60**	**QA-SA.**	**QA-SA.**	**QA-SA.**
**p-value**	3.73787E-05	6.53652E-06	0.00058839

The Table 25 shows that results are statistically significant (with p-value < 0.05) for test cases n = {20, 30, 50}, and we can conclude the proposed Quantitative Algorithm (QA) has better solution quality with reduced computational requirements and better cost improvement than benchmark heuristic Simulated Annealing (SA).

#### Simulation review

As with any heuristics and metaheuristics, it is not an exact process defined by a general linear function, but the results from the statistical analysis demonstrate the algorithm’s quality are significant. In this paper we have demonstrated the statistical significance of the results, as the p-value for the t-Test with Unequal Variances between the Simulated Annealing (SA) heuristic (Benchmark) and the proposed Quantitative Algorithm (QA), is less than the significance level for alpha with p< 0.05. The statistical test was performed for graphs with 20, 30 and 50 nodes and all trials have returned a significant result by rejecting the null hypothesis.

This supports the evidence that the schema proposed by heuristics methods such as GA, SA, AC or CE produces results that are, in average, equal or worse than the proposed QA method based in Information Theory and Entropy simulation, running for the same number of finite iterations. The best cost and running time are in average, found in the sample from the QA method, although it was also found in the control group SA, used as our benchmark. This result is expected, as outliers can happen and are expected to be found in random process in the long term.

The results obtain in this research showed a smaller runtime overhead in QA method possibly because of the simpler numerical calculations and instructions set in the code, if compared to the above-mentioned traditional heuristics. In GA, SA, AC and Neural Networks for example there are complex matrix, string and graph manipulations.

The Christofides algorithm also has to keep track and detect short-cuts between nodes in the know state search-space and thus increasing memory requirements. The proposed method does not need a buffer of illegal moves, such as proposed by the Tabu search method used to optimize the k-opt algorithm.

It also doesn’t need to continuously look for short-cuts or swapping ties, it works by generating a random sequence that encode a Hamiltonian graph (i.e. A path that visits all nodes exactly once). After generating the random candidate solution there are only 3 conditions to decide:

1. Have we reached the maximum number of iterations defined in the input parameters? If yes than halt
2. If new path is better than the current best route than updates the current control state.
3. If the candidate solution is worse, than we accept the solution, according to a decaying Bernoulli process B(X) for random variable X and a probability density function estimation with entropy H(X) with dependent utility function Y = g(X).

### Applications for the method

The Traveling Salesman Problem is the foundation for many optimization methods, algorithms, models and process with applications in many areas and fields such as

- Computer science
  ◦ Optimize package routing for sending and receiving messages in a network
  ◦ Task Scheduling for batch processing in a cluster of servers
- Logistics
  ◦ Optimize package and mail delivery between addresses in a city
  ◦ Vehicle route optimization for GPS systems
  ◦ Job shop problem to assign activities to a pool of resources
  ◦ Minimize the total execution length schedule of a set of tasks (makespan)

**Fig 41:**
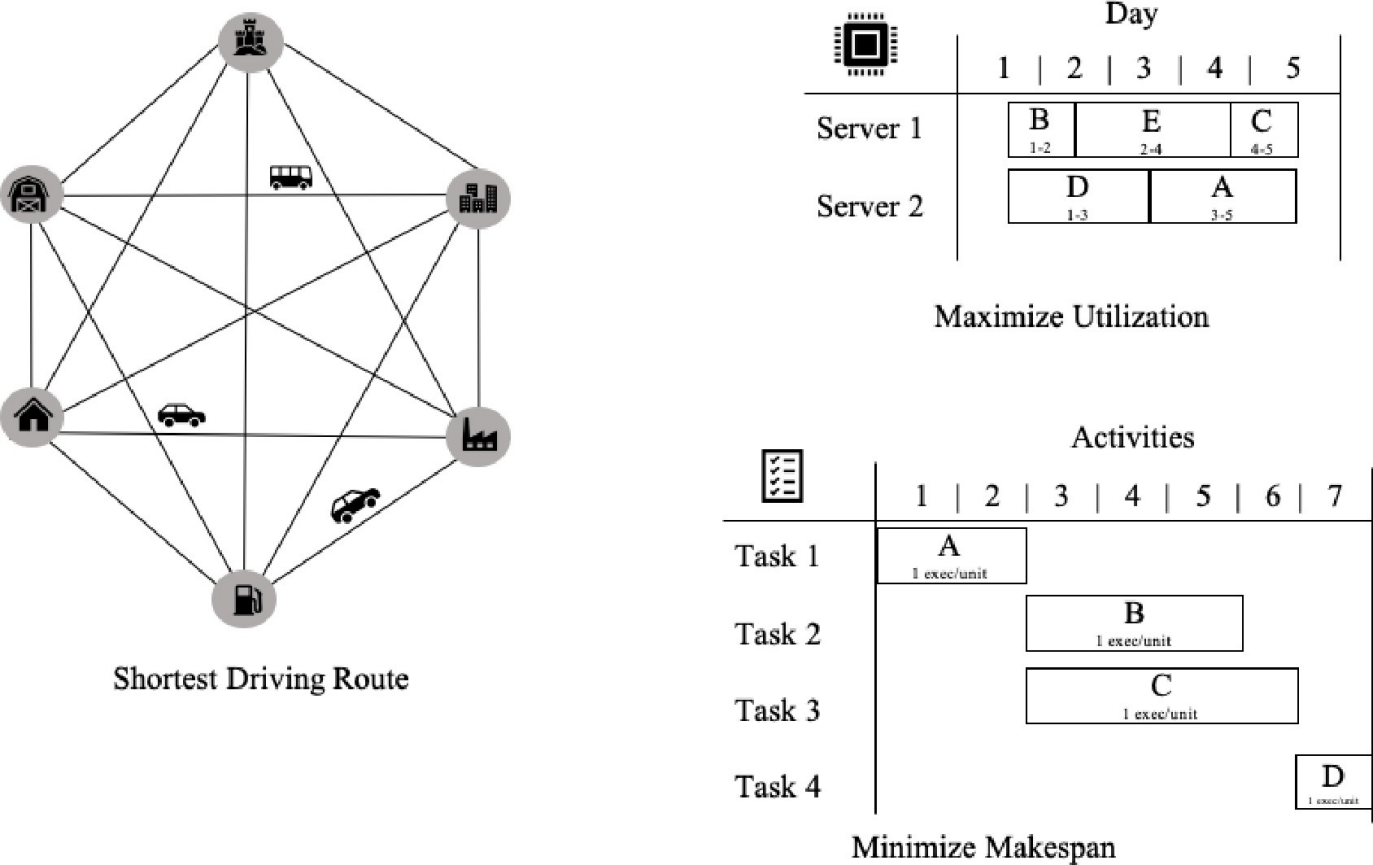
Example of TSP applications.

Fig 41 illustrates some applications of the TSP for shortest driving route, maximize server utilization and minimize makespan.

## Conclusion

Service scheduling and network routing has many applications and is related to the optimization problem modeled by the Traveling Salesman Problem. It’s possible to improve performance by reducing the cost of transmission of information across many distributed systems locations. A new interpretation and verified optimization algorithm and statistical model based in Information Theory is presented in this paper and it demonstrated how it can be used to solve the TSP. The results support the idea that the proposed method can be used reliably to generate solution under a given degree of freedom. The algorithm can be expanded to large scale problems without the high requirements of computational resources utilization imposed by the brute-force and traditional heuristics methods such as Simulated Annealing, Ant Colony, Neural Networks and Genetic Algorithms. The algorithm can be adapted to any case of the routing problem. The research can be used as a framework for future works and extend the implications of Information Theory and Kolmogorov-Complexity in solving TSP and NP Problems in general.

The advantage of this approach is that it is independent of the computer encoding the problem and the time and space complexities are additive up to a limit that is linearly proportional to the input size. Other heuristics methods assume a predefined knowledge about the data structure and thus are biased towards this encoded schema. The implications of this interpretation is that by reducing the NP problems to a matter of modularization of encoded and decoded random signals in an communication noisy channel, we can find near optimal solutions that are statistically significant and are guaranteed to produce the best rate of improvement in the long run over many trials(i.e. simulation iterations) even though the problems itself is computationally complex and the alternative sequential brute force algorithm would require exponential time to solve. This mathematical model can be interpreted as a generalization of heuristics methods.

The findings in this paper unifies critical areas in Computer Science, Mathematics and Statistics that many researchers have not explored and provided a new interpretation that advances the understanding of the role of entropy in decision problems encoded in Turing Machines.

## Supporting information

S1 File (XLSX)
